# The Transposon Registry

**DOI:** 10.1186/s13100-019-0182-3

**Published:** 2019-10-09

**Authors:** Supathep Tansirichaiya, Md. Ajijur Rahman, Adam P. Roberts

**Affiliations:** 1 Department of Tropical Disease Biology, Liverpool School of Tropical Medicine, Pembroke Place, Liverpool, L3 5QA UK; 2 Department of Clinical Dentistry, Faculty of Health Sciences, UiT the Arctic University of Norway, Tromsø, 9037 Norway; 3 Department of Pharmacy, University of Rajshahi, Rajshahi, 6205 Bangladesh

**Keywords:** Transposon, Tn, Registry, Conjugative transposon, Composite transposon, Integrative conjugative transposon, Antimicrobial resistance, Accessory function, Horizontal gene transfer, Plasmid

## Abstract

Transposable elements in prokaryotes are found in many forms and therefore a robust nomenclature system is needed in order to allow researchers to describe and search for them in publications and databases. Here we provide an update on The Transposon Registry which allocates numbers to any prokaryotic transposable element. Additionally, we present the completion of registry records for all transposons assigned Tn numbers from Tn*1* onwards where sequence data or publications exist.

## Introduction and background

A diverse array of transposable elements (TEs) can be found in the genomes of most prokaryotes where their evolutionary strategies for replication and mobility are often inextricably linked with fundamental roles in the evolution and adaptation of their hosts. Defined as “specific DNA segments that can repeatedly insert into one or more sites in one or more genomes” [1] an increasing variety of TEs are being discovered, facilitated by the normalisation of rapid whole genome sequencing and analysis. The opportunity for a researcher to be able to use a number-based, characteristic-free (in terms of the TE genome), nomenclature system to assign a unique name to a new TE means that the sequence of, and associated publications on, the newly described TE will be searchable and the nomenclature system can deal with any manner of genetic variation within TEs and their hosts.

The Transposon Registry is a nomenclature system for the assignment of Tn numbers for bacterial and archaeal autonomous TEs, including unit transposons, composite transposons, conjugative transposons (CTns)/Integrative Conjugative Elements (ICEs), Mobilisable transposons (MTns)/Integrative mobilisable elements (IMEs) and mobile genomic islands. The online registry is well established and has been utilised extensively over the last decade assigning over 700 Tn numbers to researchers to date. It excludes insertion sequences (ISs), which are managed by ISfinder database (www-is.biotoul.fr) and other TEs such as introns and inteins for which other databases already exist, and non-autonomous TEs such as integron cassettes and MITES. It is also worth noting that alternative nomenclature guidelines have been proposed for the ICEs and IMEs which uses “ICE” or “IME” followed by a two or three letter acronym of the host and a sequential number [2]. There is however cross-over between these two nomenclature systems as the “Tn” part of a newly designated Tn number from The Transposon Registry can be written as “CTn” (for conjugative transposon) or “ICE” or indeed “MTn” or “IME” as preferred [1] which enables researchers to cope with findings from metagenomes where host identification may not be possible. In this short review we provide a historical perspective, thoughts on future directions for TE annotation and nomenclature and an update on The Transposon Registry.

## What’s in a name?

Why should researchers contemplate naming a newly identified TE? It is useful to name a mobile genetic element such as a TE so the discoverer and other researchers can specifically refer to it. This is becoming increasingly important as our understanding about the influence of TEs upon their hosts becomes more apparent. Epidemiological and evolutionary studies throughout biology require names in order to refer to any mobile and / or evolving entity, whether this is a macro-scale population or a molecular scale unit such as DNA. This is particularly appropriate when describing mobile genetic elements due to their recombinatorial nature, and their ability to spread prolifically between different hosts, and within their hosts around the planet. Using higher order names such as composite transposon or resistance plasmid does not allow for the fine scale details to be described following comparative analysis within the increasingly large DNA sequence databases and the proliferation of microbial genome sequences.

## A historical perspective on Tn numbers

The nomenclature of transposable elements was first discussed in a meeting on DNA Insertions at Cold Spring Harbor in 1976. A set of rules for the nomenclature were modified based on the proposal from D.E. Berg and W. Szybalski, which was subsequently revised in 1979, due to development of early DNA sequencing techniques [3, 4]. Insertion sequences and transposable elements were named separately by having IS and Tn as a prefix, respectively, followed by a sequential number in italics such as IS*1*, IS*2* and Tn*1*, Tn2, etc. The administration and allocation of numbers were carried out by the late Dr. Esther Lederberg from Stanford University Medical School, CA, USA. The names and locations of registrants for Tn*1* to Tn*4685* were published previously [5, 6]. The allocations were continued up to Tn*5500* and above but were not published as a list and allocation ceased when Dr. Lederberg stopped running the plasmid reference centre.

**Fig. 1:**
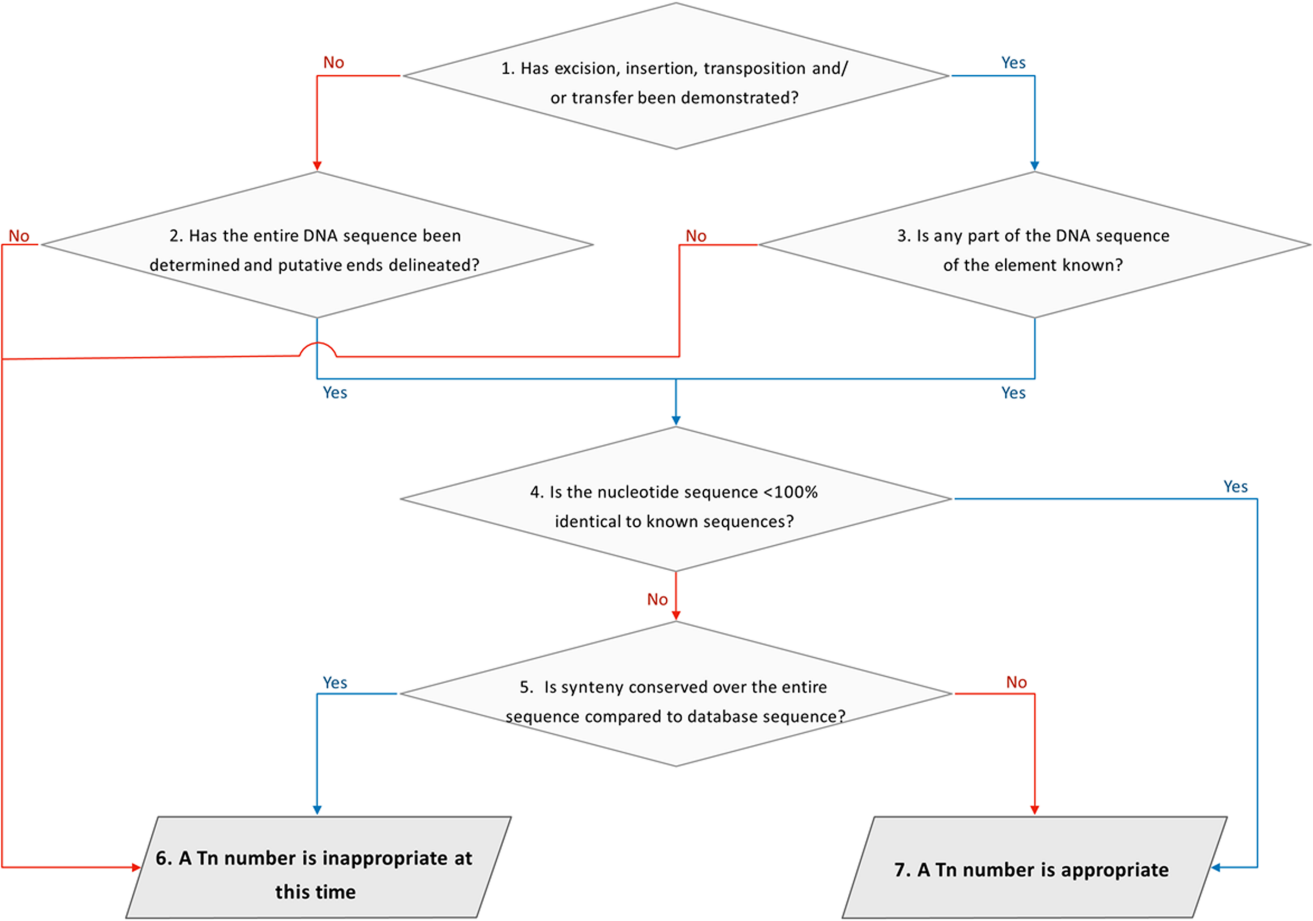
Flow chart for the determining whether a transposon suitable for a new Tn number based on the guidelines published previously [1]

Subsequently, a variety of nomenclature systems were adopted by different research groups, especially for novel types of TE, due to the discontinuation of the sequential numbering system and the need to name mobile genetic elements being studied as outlined above. To fill this gap the “Tn Registry” was launched in 2006, hosted by University College London, London, UK and an accompanying description published in 2008 [1]. It began assigning Tn numbers from Tn*6000* to avoid any duplicative assignations as there are published records within the 5000 range (see Additional file 1: Table S1). A set of criteria, was also proposed to determine whether a new Tn number is appropriate [1], summarized in Fig. 1.

## The Transposon Registry

In 2017 the Tn registry moved to Liverpool School of Tropical Medicine (LSTM), Liverpool, UK, (https://transposon.lstmed.ac.uk/) and was upgraded, updated and renamed “The Transposon Registry”. The registry is now fully searchable and can be updated by users as and when their sequences are deposited and when their publications arise by linking their individual records to accession numbers and digital object identifiers respectively. In order to obtain a Tn number, users simply register and request as many as they need when they need them; there is no reason to request surplus numbers as we are not going to run out. Subsequent to its relocation to LSTM, we have collated and backfilled The Transposon Registry with all published records from Tn*1* to Tn*5999*, where either a publication or a sequence is available. It is worth noting that many records assigned previously do not have a sequence or publication associated with them. This we think is in part because researchers were assigned blocks of numbers by traditional postal methods to use as and when they were needed, something which is no longer necessary due to the online nature of The Transposon Registry. We have also updated the records from Tn*6000* onwards where users have not yet had the opportunity to do so.

**Fig. 2:**
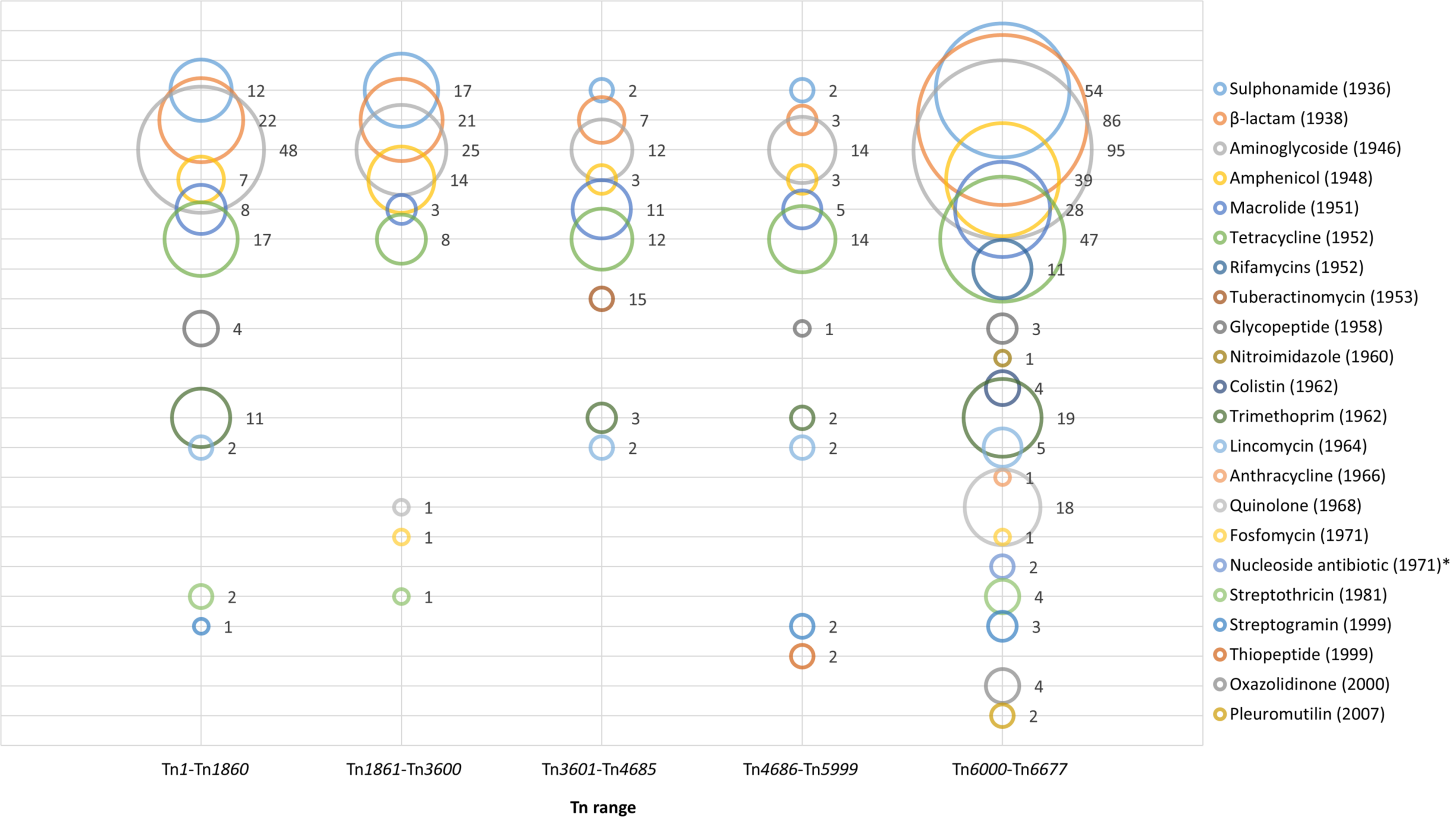
Bubble chart depicting the number of Tn records containing resistance genes against different types of antibiotic classes. Antibiotic classes were sorted according to the year of introduction on the Y-axis. Tn records were grouped into 5 groups on the X-axis according to the previously published lists [3–5], and before and after the allocation by the Tn registry (starting at Tn*6000*). The number of Tn records is represented by the bubble size and also indicated on the right of each bubble. * year of discovery as approval never received

**Fig. 3:**
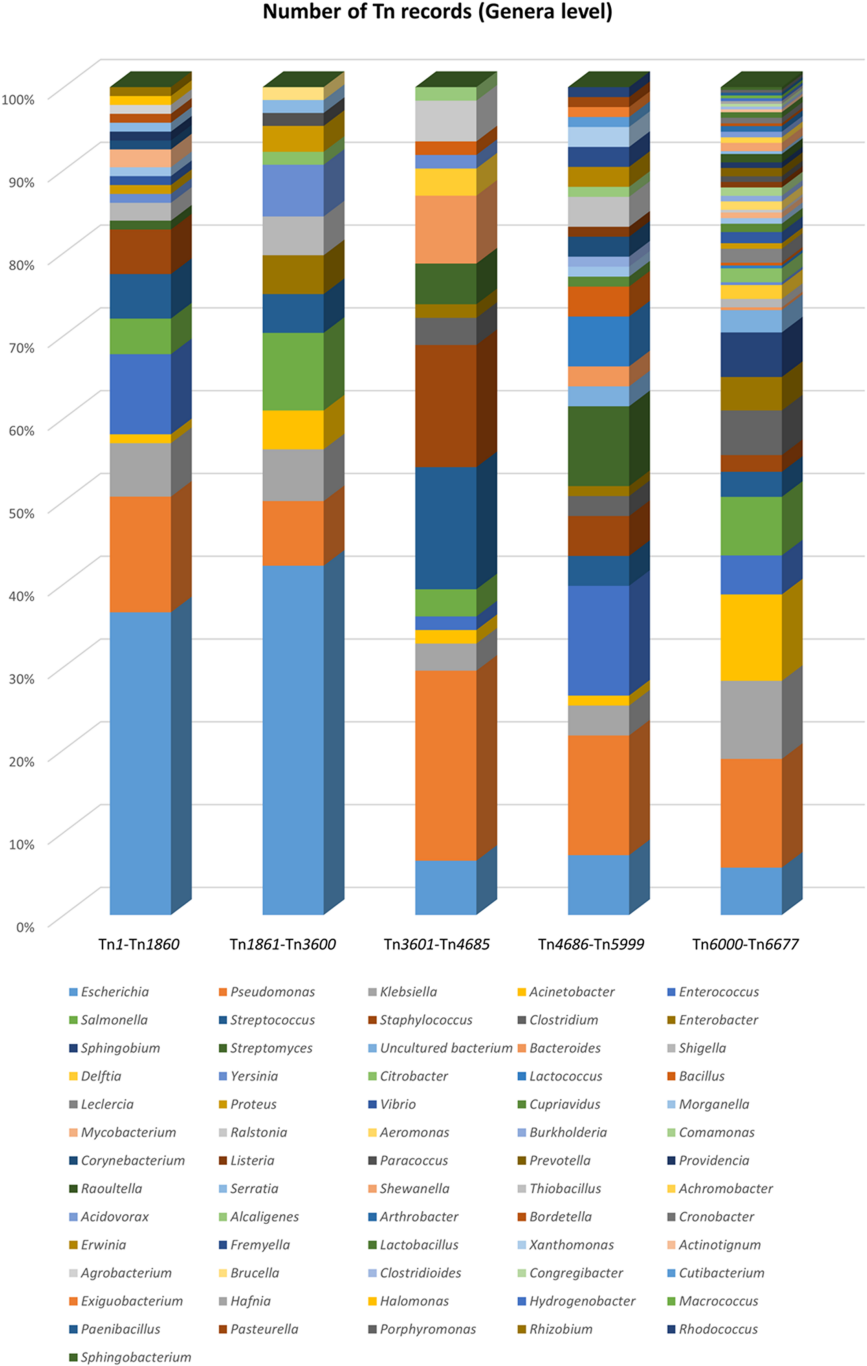
Number of Transposon records displayed by host at genera level. The range within the 5 columns are representative of the previously published lists [3–5] and before and after allocation by the Tn Registry (starting at Tn*6000*)

The current, complete information on all available records in The Transposon Registry is summarized in Table 1, including available metadata on the types, size, original host, accession numbers, accessory functions and references (Table 1). The accessory functions assigned to cargo genes primarily include antimicrobial resistance and show that antibiotic resistance genes against all major antibiotic classes are increasingly found to be associated with transposons and an increase in the numbers of antibiotics to which resistance is conferred by transposon located genes (Fig. 2 and Additional file 2: Table S2). Also noted are antiseptic resistance, heavy metal resistance, efflux functions, metabolic capability and virulence factor and CRISPR functions. It is interesting to note the trends observable with respect to the hosts of transposons being reported (Fig. 3). It is clear that more transposons are being reported in *Acinetobacter* and *Klebsiella* and that the diversity of transposon hosts is increasing, presumably as sequencing becomes more common place*.* As the identification of the host species is not a requirement for the nomenclature system, transposable elements identified from metagenomic studies are also included, e.g. Tn*6032* and Tn*6300*. Details of all the entries within the Transposon Registry are provided in the Additional file 1: Table S1.

**Table 1:** Summary of all available records in The Transposon Registry based on the data in Additional file 1

	Characteristics		Number
Available records in The Transposon Registry (Records)	Tn*1*-Tn*1860* [3]		102
	Tn*1861*-Tn*3600* [5]		64
	Tn*3601*-Tn*4685* [6]		62
	Tn*4686*-Tn*5999*^(Not published)^		83
	Tn*6000*-Tn*6677*^(This work)^		299
	Total		610
Types and Size of transposons	Unit transposons	Number of records	313
		Average (range) in kb	12.4 (2.4–86)
	Composite transposons	Number of records	136
		Average (range) in kb	11.7 (1.5–65)
	Conjugative transposons	Number of records	97
		Average (range) in kb	45.7 (9.6–120)
	Composite transposon and unit transposon	Number of records	23
		Average (range) in kb	36.6 (8.4–111.2)
	Not specified	Number of records	41
	Overall	Number of records	610
		Average (range) in kb	19.1 (1.5–120)
Accessory functions (Records)	AMR		399
	Antiseptic resistance		18
	Efflux functions		31
	Heavy metal resistance		32
	Metabolism		15
	Virulence determinants		127
	CRISPR		1
	Other / not defined		97
Accession numbers (Records)	Available in GenBank		448
	Not available in GenBank		162
Publication (Records)	Tn with available publication		583
	No publication		27

## Future perspectives and conclusions

As research into TE biology continues its migration from primarily experimental biology, where mobility is proven, to a more comparative genomics approach it is likely that the available bioinformatic tools which are used to identify TEs (reviewed in [7] and references therein) will be improved, both in terms of their application for interrogating different host species, and delineating different classes of TE. The complexity of TEs; their variety and biology, continue to be better understood and the nomenclature will need to catch up to cope with this increasing knowledge [7]. It is worth reiterating here, the call for a formal discussion within the international community to fully address the problematic issues of TE nomenclature and come up with an agreeable system which can accommodate all classes of TE we see [7, 8] and be future proof to accommodate those that we as yet can only imagine. Until that time, we will continue to operate and update The Transposon Registry with any missing or inaccurate records highlighted by the community. We welcome inclusion of TEs named using other systems to be included within The Transposon Registry and call on the community to provide these details if they wish for them to be included.

## Supplementary information

**Additional file 1: Table S1.** Details of transposon records available in The Transposon Registry (DOCX 529 kb)

**Additional file 2: Table S2.** List of transposons associated with antibiotic resistance genes (DOCX 24 kb)

## Data Availability

The dataset used to generate Figs. 2 and 3 and all tables is available in the Additional file 1: Table S1 and Additional file 2: Table S2.
