# Supplementary material for: The Transposon Registry
Source: Mob DNA. 2019 Oct 9;10:40. doi: 10.1186/s13100-019-0182-3 (PMC6785933; doi:10.1186/s13100-019-0182-3)
Supplement: Supplementary file 1 — Additional file 1: Table S1. Details of transposon records available in The Transposon Registry (DOCX 529 kb) [file 13100_2019_182_MOESM1_ESM.docx]

**Additional file 1: Table S1 Details of transposon records available in The Transposon Registry**

| Transposon name | Origin | Type | Size (kb) | Accession number | Accessory Functions | References |
| --- | --- | --- | --- | --- | --- | --- |
| Tn*1* | *Escherichia coli* | Unit transposon | 3.6 | L10085.1 | AMR | (1) |
| Tn*2* | *Escherichia coli* | Unit transposon | 5 | HM749967.1 | AMR | (2) |
| Tn*3* | *Escherichia coli* | Unit transposon | 4.96 | V00613.1 | AMR | (3) |
| Tn*4* | *Escherichia coli* | Unit transposon | - | HM749966.1, HQ201416.1 | AMR, Heavy metal resistance | (4) |
| Tn*5* | *Escherichia coli* | Composite transposon | 5.8 | U00004.1 | AMR | (5) |
| Tn*6* | *Escherichia coli* | Composite transposon | 5 | - | AMR | (6) |
| Tn*7* | *Escherichia coli* | Unit transposon | 14 | KX159450.1 | AMR | (7) |
| Tn*9* | *Escherichia coli* | Composite transposon | 2.6 | V00622.1 | AMR | (8) |
| Tn*10* | *Shigella flexneri* | Composite transposon | 9.3 | AF162223.1 | AMR | (9) |
| Tn*21* | *Escherichia coli* | Unit transposon | 19.7 | AF071413.3 | AMR, Heavy metal resistance | (10) |
| Tn*45* | *Corynebacterium glutamicum* | Unit transposon | 2.4 | U85507.1 | AMR | (11) |
| Tn*55* | *-* | Unit transposon | - | - | AMR | (12) |
| Tn*71* | *Escherichia coli* | Unit transposon |  | AP002527.1 | AMR | (13) |
| Tn*72* | *-* | Unit transposon | - | - | AMR | (13) |
| Tn*76* | *Klebsiella aerogenes* | Unit transposon |  | - | AMR | (14) |
| Tn*125* | *Acinetobacter baumannii* | Composite transposon | 10 | JN872328.1 | AMR | (15) |
| Tn*163* | *Rhizobium leguminosarum* | Unit transposon | 4.6 | L14931.1 | - | (16) |
| Tn*501* | *Pseudomonas aeruginosa* | Unit transposon | 8.3 | Z00027.1 | Heavy metal resistance | (17) |
| Tn*502* | *Pseudomonas aeruginosa* | Unit transposon | 9.6 | EU306743.1 | Heavy metal resistance | (18) |
| Tn*510* | *Bordetella bronchiseptica* | Unit transposon | 13.3 | DQ471307.1 | AMR, Heavy metal resistance | (19) |
| Tn*511* | *Proteus mirabilis* | Unit transposon | 19 | EU287476.1 | AMR, Heavy metal resistance | (19) |
| Tn*512* | *Pseudomonas sp* | Unit transposon | 8.4 | EU306744.1 | Heavy metal resistance | (20) |
| Tn*536* | *Shigella flexneri* | Not specified | 16 | - | AMR, Heavy metal resistance | (21) |
| Tn*551* | *Staphylococcus aureus* | Unit transposon | 5.2 | Y13600.1 | AMR | (22) |
| Tn*552* | *Staphylococcus aureus* | Unit transposon | 6.5 | X52734.1 | AMR | (23) |
| Tn*554* | *Staphylococcus aureus* | Unit transposon | 6.7 | X03216.1 | AMR | (24) |
| Tn*558* | *Staphylococcus lentus* | Unit transposon | 6.6 | AJ715531.1 | AMR | (25) |
| Tn*559* | *Staphylococcus aureus* | Unit transposon | 4.2 | FN677369.2 | AMR | (26) |
| Tn*601* | *Escherichia coli* | Composite transposon | - | - | AMR | (27) |
| Tn*602* | *Escherichia coli* | Composite transposon | 3 | AH000951.1 | AMR | (28) |
| Tn*610* | *Mycobacterium smegmatis* | Composite transposon | 3.97 | X53635.1 | AMR | (29) |
| Tn*611* | *Mycobacterium smegmatis* | Composite transposon | - | - | AMR | (30) |
| Tn*654* | *Escherichia coli* | Unit transposon | - | - | AMR | (31) |
| Tn*679* | *Escherichia coli* | Composite transposon | - | - | AMR | (32) |
| Tn*701* | *Morganella morganii* | Unit transposon | 8.5 | - | AMR | (33) |
| Tn*732* | *Klebsiella sp.* | Not specified | - | - | AMR | (34) |
| Tn*733* | *-* | Not specified | - | - | AMR | (35) |
| Tn*734* | *-* | Not specified | - | - | AMR | (35) |
| Tn*735* | *Escherichia coli* | Unit transposon | 15 | - | AMR, Heavy metal resistance | (36) |
| Tn*800* | *Escherichia coli* | Not specified | - | - | AMR | (36) |
| Tn*801* | *Pseudomonas aeruginosa* | Unit transposon | 4.95 | AF080442.1 | AMR | (37) |
| Tn*802* | *Escherichia coli* | Unit transposon | - | - | AMR | (38) |
| Tn*804* | *Escherichia coli* | Not specified | - | - | AMR | (39) |
| Tn*813* | *-* | Unit transposon | - | - | AMR, Heavy metal resistance | (40) |
| Tn*841* | *Klebsiella oxytoca* | Unit transposon | 7 | M88143.1 | AMR | (41) |
| Tn*901* | *Salmonella panama* | Unit transposon | - | - | AMR | (42) |
| Tn*902* | *Escherichia coli* | Unit transposon | - | - | AMR | (43) |
| Tn*903* | *Escherichia coli* | Composite transposon | 3 | V00359.1 | AMR | (44) |
| Tn*904* | *Pseudomonas sp.* | Not specified | 5.2 |  | AMR | (45) |
| Tn*916* | *Enterococcus faecalis* | Conjugative transposon | 18 | KM516885.1 | AMR | (46) |
| Tn*917* | *Enterococcus faecalis* | Unit transposon | 5.2 | M11180.2 | AMR | (47) |
| Tn*918* | *Enterococcus faecalis* | Conjugative transposon | 16.8 | - | AMR | (48) |
| Tn*919* | *Streptococcus sanguis* | Conjugative transposon | - | - | AMR | (49) |
| Tn*924* | *Enterococcus faecalis* | Composite transposon | 27 | - | AMR | (50) |
| Tn*925* | *Enterococcus faecalis* | Conjugative transposon | 18 | AY855841.2 | AMR | (51) |
| Tn*950* | *Enterococcus faecium* | Conjugative transposon | 47 | - | AMR | (52) |
| Tn*951* | *Yersinia enterocolitica* | Unit transposon | 16.6 | - | Metabolism | (53) |
| Tn*981* | *Escherichia coli* | Composite transposon | 2.3 | - | AMR | (54) |
| Tn*1000* | *Escherichia coli* | Unit transposon | 5.9 | X60200.1 | - | (55) |
| Tn*1013* | *Pseudomonas aeruginosa* | Unit transposon | 8.2 | AM261760.1 | Efflux function | (56) |
| Tn*1021* | *Escherichia coli* | Not specified | 6 | - | AMR | (57) |
| Tn*1022* | *Escherichia coli* | Not specified | 10 | - | AMR | (57) |
| Tn*1116* | *Streptococcus pyogenes* | Conjugative transposon | 23 | AM411377.1 | AMR | (58) |
| Tn*1213* | *Pseudomonas aeruginosa* | Unit transposon | 4.2 | AY779042.1 | AMR | (59) |
| Tn*1207.1* | *Streptococcus pneumoniae* | Composite transposon | 7.2 | AF227520.1 | AMR, Efflux function | (60) |
| Tn*1331* | *Klebsiella pneumoniae* | Unit transposon | 8 | NC_003486.1 | AMR | (61) |
| Tn*1332* | *Pseudomonas putida* | Unit transposon | 11 | DQ174113.1 | AMR | (62) |
| Tn*1401* | *Salmonella typhimurium* | Unit transposon | 15.9 | - | AMR, Heavy metal resistance | (63) |
| Tn*1402* | *-* | Unit transposon | - | - | AMR, Heavy metal resistance | (63) |
| Tn*1403* | *Pseudomonas aeruginosa* | Unit transposon | 19.6 | AF313472 | AMR | (64) |
| Tn*1404* | *Pseudomonas sp.* | Unit transposon | 19 | AH008062.3 | AMR | (65) |
| Tn*1405* | *Pseudomonas aeruginosa* | Not specified | 8 | - | AMR | (66) |
| Tn*1523* | *Escherichia coli* | Not specified | 10.8 | - | AMR | (67) |
| Tn*1525* | *Salmonella panama* | Composite transposon | 4.44 | - | AMR | (68) |
| Tn*1527* | *Vibrio cholerae* | Not specified | 14 | - | AMR | (69) |
| Tn*1528* | *Providencia stuartii* | Composite transposon | 5.2 | - | AMR | (70) |
| Tn*1545* | *Streptococcus pneumoniae* | Conjugative transposon | 25.3 | X61025.1 | AMR | (71) |
| Tn*1546* | *Enterococcus faecium* | Unit transposon | 10.8 | M97297.1 | AMR | (72) |
| Tn*1547* | *Enterococcus faecalis* | Composite transposon | 64 | - | AMR | (73) |
| Tn*1548* | *Klebsiella pneumoniae* | Composite transposon | 16.6 | - | AMR | (74) |
| Tn*1549* | *Enterococcus faecalis* | Conjugative transposon | 33.8 | AF192329.1 | AMR | (75) |
| Tn*1681* | *Escherichia coli* | Composite transposon | 25 | L36547.1 | Virulence determinants | (76) |
| Tn*1696* | *Pseudomonas aeruginosa* | Unit transposon | 13.6 | U12338.3 | AMR, Heavy metal resistance | (77) |
| Tn*1699* | *Klebsiella pneumoniae* | Not specified | 9.3 | - | AMR | (78) |
| Tn*1700* | *Serratia marcescens* | Not specified | 12 | - | AMR | (78) |
| Tn*1701* | *Salmonella typhimurium* | Not specified | - | - | AMR | (79) |
| Tn*1720* | *Pseudomonas sp.* | Unit transposon | 9.3 | AH008063.2 | AMR | (65) |
| Tn*1721* | *Escherichia coli* | Unit transposon | 11.1 | X61367.1 | AMR | (80) |
| Tn*1722* | *Escherichia coli* | Unit transposon | 5.5 | - | - | (80) |
| Tn*1723* | *Escherichia coli* | Unit transposon | 3.9 | - | AMR | (81) |
| Tn*1725* | *Escherichia coli* | Unit transposon | 8.3 | - | AMR | (82) |
| Tn*1732* | *Halomonas elongata* | Unit transposon | - | - | AMR | (83) |
| Tn*1755* | *Escherichia coli* | Unit transposon | - | - | AMR | (84) |
| Tn*1756* | *Escherichia coli* | Unit transposon | - | - | AMR | (85) |
| Tn*1771* | *Escherichia coli* | Not specified | - | - | AMR | (86) |
| Tn*1792* | *Streptomyces lividans* | Composite transposon | 2.8 | AJ006518.1 | AMR | (87) |
| Tn*1806* | *Streptococcus pneumoniae* | Conjugative transposon | 52.4 | CP002121.1 | AMR | (88) |
| Tn*1822* | *-* | Unit transposon | - | - | AMR | (89) |
| Tn*1824* | *-* | Unit transposon | - | - | AMR | (89) |
| Tn*1825* | *Escherichia coli* | Unit transposon | 13.2 | - | AMR | (90) |
| Tn*1826* | *-* | Unit transposon | - | - | AMR | (91) |
| Tn*1831* | *Agrobacterium tumefaciens* | Not specified | - | - | AMR, Heavy metal resistance | (92) |
| Tn*1935* | *Salmonella wien* | Unit transposon | 23.5 | - | AMR, Heavy metal resistance | (93) |
| Tn*1999* | *Klebsiella pneumoniae* | Composite transposon | 4.9 | AY236073.2 | AMR | (94) |
| Tn*2000* | *Escherichia coli* | Composite transposon | 12 | AF205943.1 | AMR, Antiseptic resistance | (95) |
| Tn*2001* | *Pseudomonas aeruginosa* | Not specified | - | - | AMR | (96) |
| Tn*2003* | *Enterobacter cloacae* | Composite transposon | 9.5 | AY532647.1 | AMR | (97) |
| Tn*2006* | *Acinetobacter baumannii* | Composite transposon | 4.8 | EF127491.1 | AMR | (98) |
| Tn*2007* | *Acinetobacter baumannii* | Composite transposon | 2.5 | EF059914.1 | AMR | (98) |
| Tn*2008* | *Acinetobacter baumannii* | Unit transposon | 4 | GQ861438.1 | AMR | (99) |
| Tn*2009* | *Streptococcus pneumoniae* | Conjugative transposon | 23 | AF376746.2 | AMR | (100) |
| Tn*2010* | *Streptococcus pneumoniae* | Conjugative transposon | 26.4 | AB426620.1 | AMR | (101) |
| Tn*2011* | *Escherichia coli* | Not specified | - | - | AMR, Heavy metal resistance | (102) |
| Tn*2012* | *Escherichia coli* | Unit transposon | 2.7 | EU523120.1 | AMR | (103) |
| Tn*2016* | *Klebsiella pneumoniae* | Unit transposon | 4.4 | JQ809466.1 | AMR | (104) |
| Tn*2017* | *Streptococcus pneumoniae* | Conjugative transposon | 28.5 | FJ208941.1 | AMR | (105) |
| Tn*2020* | *Brucella abortus* | Composite transposon | 4.1 | AF118548.1 | - | (106) |
| Tn*2101* | *Enterobacter cloacae* | Not specified | - | - | AMR, Heavy metal resistance | (107) |
| Tn*2301* | *Escherichia coli* | Not specified | . | - | AMR | (108) |
| Tn*2350* | *Escherichia coli* | Composite transposon | 10.4 | - | AMR | (109) |
| Tn*2353* | *Escherichia coli* | Not specified | 13 | - | AMR, Heavy metal resistance | (110) |
| Tn*2354* | *Escherichia coli* | Not specified | 7.8 | - | - | (111) |
| Tn*2401* | *Pseudomonas aeruginosa* | Composite transposon | 7.1 | - | AMR | (112) |
| Tn*2410* | *Salmonella typhimurium* | Unit transposon | 18.5 | - | AMR, Heavy metal resistance | (113) |
| Tn*2411* | *Salmonella typhimurium* | Unit transposon | 18 | - | AMR, Heavy metal resistance | (113) |
| Tn*2424* | *Escherichia coli* | Unit transposon | 25 | AF047479.2 | AMR, Heavy metal resistance | (114) |
| Tn*2425* | *Escherichia coli* | Unit transposon | - | - | AMR, Heavy metal resistance | (115) |
| Tn*2426* | *Shigella sonnei* | Unit transposon | 3.5 | M86913.1 | AMR, Heavy metal resistance | (116) |
| Tn*2440* | *Escherichia coli* | Composite transposon | 4 | - | AMR | (117) |
| Tn*2501* | *Yersinia sp.* | Unit transposon | 6.3 | M15197.1 | - | (118) |
| Tn*2502* | *Yersinia enterocolitica* | Unit transposon | 4.6 | U58366.1 | Heavy metal resistance | (119) |
| Tn*2503* | *Yersinia pestis* | Unit transposon | 6.8 | CP002179.1 | Heavy metal resistance | (120) |
| Tn*2505* | *Escherichia coli* | Unit transposon | 8.2 | - | - | (121) |
| Tn*2506* | *Escherichia coli* | Unit transposon | 7.3 | - | AMR | (121) |
| Tn*2507* | *Yersinia enterocolitica* | Unit transposon | 9.1 | - | AMR | (122) |
| Tn*2515* | *Escherichia coli* | Unit transposon | - | - | AMR | (121) |
| Tn*2516* | *Escherichia coli* | Unit transposon | - |  | AMR | (121) |
| Tn*2521* | *Pseudomonas aeruginosa* | Not specified | 7 | AF313471.1 | AMR | (66) |
| Tn*2523* | *Pseudomonas aeruginosa* | Not specified | - | - | AMR | (123) |
| Tn*2601* | *Escherichia coli* | Unit transposon | 4.7 | - | AMR | (124) |
| Tn*2602* | *Escherichia coli* | Unit transposon | 4.7 | - | AMR | (124) |
| Tn*2603* | *Escherichia coli* | Unit transposon | 20 | - | AMR, Heavy metal resistance | (125) |
| Tn*2607* | *Escherichia coli* | Unit transposon | 24 |  | AMR | (126) |
| Tn*2608* | *Klebsiella pneumoniae* | Unit transposon | 13.5 |  | AMR, Heavy metal resistance | (126) |
| Tn*2610* | *Escherichia coli* | Unit transposon | 23.9 | AB207867.1 | AMR | (127) |
| Tn*2613* | *Proteus mirabilis* | Unit transposon | 7.2 | - | Heavy metal resistance | (126) |
| Tn*2653* | *Escherichia coli* | Composite transposon | 5.9 | - | AMR | (128) |
| Tn*2657* | *Escherichia coli* | Unit transposon | - | - | AMR | (129) |
| Tn*2660* | *Escherichia coli* | Unit transposon | 4.8 | - | AMR | (130) |
| Tn*2670* | *Shigella flexneri* | Composite transposon and Unit transposon | 19.6 | AP000342.1 | AMR, Heavy metal resistance | (131) |
| Tn*2671* | *Shigella flexneri* | Composite transposon | 23 | AP000342.1 | AMR, Heavy metal resistance | (132) |
| Tn*2672* | *Escherichia coli* | Composite transposon and Unit transposon | 28 | - | AMR, Heavy metal resistance | (133) |
| Tn*2680* | *Proteus vulgaris* | Composite transposon | 5 | NC_003905.1 | AMR | (134) |
| Tn*2681* | *Escherichia coli* | Composite transposon | - | - | AMR | (134) |
| Tn*2700* | *Salmonella typhimurium* | Composite transposon | - | - | AMR | (135) |
| Tn*2901* | *Escherichia coli* | Composite transposon | 11 | - | - | (136) |
| Tn*2921* | *Serratia marcescens* | Composite transposon | 12.4 | FJ829469.1 | AMR | (137) |
| Tn*2922* | *Escherichia coli* | Composite transposon | 27 | - | AMR | (138) |
| Tn*3000* | *Enterobacter hormaechei* | Composite transposon | 11.8 | KR822246.1 | AMR | (139) |
| Tn*3351* | *Salmonella naestved* | Not specified | - | - | AMR | (140) |
| Tn*3352* | *Salmonella naestved* | Not specified | - | - | AMR | (140) |
| Tn*3401* | *Pseudomonas fluorescens* | Not specified | - | - | Heavy metal resistance | (141) |
| Tn*3402* | *Citrobacter sp.* | Not specified | - | - | Heavy metal resistance | (141) |
| Tn*3403* | *Klebsiella sp.* | Not specified | - | - | Heavy metal resistance | (141) |
| Tn*3411* | *Escherichia coli* | Composite transposon | 7.4 | M19532.1, M22041.1 | Metabolism | (142) |
| Tn*3434* | *Paracoccus pantotrophus* | Unit transposon | 3.7 | AY232820.1 | - | (143) |
| Tn*3611* | *Pseudomonas aeruginosa* | Unit transposon | 9 | - | Heavy metal resistance | (144) |
| Tn*3613* | *Pseudomonas aeruginosa* | Unit transposon | 24 | - | AMR, Heavy metal resistance | (144) |
| Tn*3614* | *Pseudomonas putida* | Unit transposon | 7.2 | - | - | (145) |
| Tn*3651* | *Escherichia coli* | Unit transposon | - | - | AMR | (146) |
| Tn*3701* | *Streptococcus pyogenes* | Conjugative transposon | 50 | - | AMR | (147) |
| Tn*3702* | *Enterococcus faecalis* | Conjugative transposon | 18.5 | - | AMR | (148) |
| Tn*3703* | *Streptococcus pyogenes* | Conjugative transposon | 19.7 | - | AMR | (147) |
| Tn*3704* | *Streptococcus anginosus* | Conjugative transposon | 20.3 | - | AMR | (149) |
| Tn*3705* | *Streptococcus anginosus* | Conjugative transposon | 52 | - | AMR | (149) |
| Tn*3706* | *Streptococcus agalactiae* | Composite transposon | - | - | AMR | (150) |
| Tn*3707* | *Streptococcus agalactiae* | Conjugative transposon | - | - | AMR | (150) |
| Tn*3851* | *Staphylococcus aureus* | Not specified | 5.2 | - | AMR | (151) |
| Tn*3853* | *Staphylococcus aureus* | Unit transposon | - | - | AMR | (152) |
| Tn*3854* | *Staphylococcus aureus* | Not specified | 4.5 | - | AMR | (153) |
| Tn*3871* | *Streptococcus faecalis* | Unit transposon | 5.1 | - | AMR | (154) |
| Tn*3872* | *Streptococcus pneumoniae* | Conjugative transposon | 23.3 | - | AMR | (155) |
| Tn*3926* | *Yersinia enterocolitica* | Unit transposon | 7.8 | X78059.1 | Heavy metal resistance | (156) |
| Tn*3951* | *Streptococcus pneumoniae* | Conjugative transposon | 65 | - | AMR | (157) |
| Tn*4000* | *Klebsiella pneumoniae* | Unit transposon | - | X12618.1 | AMR, Heavy metal resistance | (158) |
| Tn*4001* | *Staphylococcus aureus* | Composite transposon | 4.7 | GU565967.1 | AMR | (159) |
| Tn*4002* | *Staphylococcus aureus* | Not specified | 6.7 | X16471.1 | AMR | (160) |
| Tn*4003* | *Staphylococcus aureus* | Composite transposon | 4.7 | GU565967.1 | AMR | (161) |
| Tn*4031* | *Staphylococcus epidermidis* | Composite transposon | 5 | - | AMR | (162) |
| Tn*4132* | *Escherichia coli* | Unit transposon | 16 | - | AMR | (163) |
| Tn*4176* | *Alcaligenes faecalis* | Composite transposon | - | - | AMR | (164) |
| Tn*4201* | *Staphylococcus aureus* | Not specified | 6.7 | - | AMR | (165) |
| Tn*4291* | *Staphylococcus aureus* | Not specified | - | - | AMR | (166) |
| Tn*4321* | *Enterobacter aerogenases* | Composite transposon | 4.7 | U60777.1 | - | (167) |
| Tn*4351* | *Bacteroides fragilis* | Composite transposon | 5 | M17124.1 | AMR | (168) |
| Tn*4352* | *Salmonella typhimurium* | Composite transposon | 2.68 | M20306.1 | AMR | (169) |
| Tn*4371* | *Ralstonia eutropha* | Conjugative transposon | 55 | Y10831.1, Y10832.1 | Metabolism | (170) |
| Tn*4378* | *Ralstonia metallidurans* | Unit transposon | 8.2 | X90708.2 | Heavy metal resistance | (171) |
| Tn*4380* | *Ralstonia metallidurans* | Unit transposon |  | X71400.2 | Heavy metal resistance | (171) |
| Tn*4399* | *Bacteroides fragilis* | Conjugative transposon | 9.6 | L20975.1 | - | (172) |
| Tn*4400* | *Bacteroides fragilis* | Composite transposon | - | - | AMR | (173) |
| Tn*4401* | *Klebsiella pneumoniae* | Unit transposon | 10 | KT378596.1 | AMR | (174) |
| Tn*4411* | *Acinetobacter calcoaceticus* | Not specified | - | - | AMR | (175) |
| Tn*4430* | *Bacillus thuringiensis* | Unit transposon | 4.2 | X07651.1 | - | (176) |
| Tn*4431* | *Escherichia coli* | Unit transposon | - | - | AMR | (177) |
| Tn*4451* | *Clostridium perfringens* | Unit transposon | 6.3 | U15027.1 | AMR | (178) |
| Tn*4452* | *Clostridium perfringens* | Unit transposon | 6.2 | - | AMR | (179) |
| Tn*4521* | *Escherichia coli* | Composite transposon | - | M35123.1, AH000905.2 | Virulence determinants | (180) |
| Tn*4527* | *Salmonella typhimurium* | Composite transposon | 19.6 | - | AMR | (181) |
| Tn*4551* | *Bacteroides fragilis* | Composite transposon | 7.6 | M17808.1 | AMR | (182) |
| Tn*4555* | *Bacteroides vulgatus* | Unit transposon | 12.1 | U75371.3 | AMR | (183) |
| Tn*4556* | *Streptomyces fradiae* | Unit transposon | 6.8 | LC417441.1 | - | (184) |
| Tn*4560* | *Streptomyces fradiae* | Unit transposon | 8.6 | - | AMR | (185) |
| Tn*4563* | *Streptomyces coelicolor* | Unit transposon | 8.6 | - | AMR | (186) |
| Tn*4651* | *Pseudomonas putida* | Unit transposon | 56 | NC_003350.1 | Metabolism | (187) |
| Tn*4652* | *Pseudomonas putida* | Unit transposon | 17 | AF151431.1 | Metabolism | (188) |
| Tn*4653* | *Pseudomonas putida* | Unit transposon | 70 | NC_003350.1 | Metabolism | (188) |
| Tn*4654* | *-* | Unit transposon | - | - | - | (188) |
| Tn*4655* | *Pseudomonas putida* | Unit transposon | 39 | AB237655.1 | Metabolism | (189) |
| Tn*4656* | *Pseudomonas putida* | Unit transposon | 39 | AB062597.1 | Metabolism | (190) |
| Tn*4657* | *Pseudomonas putida* | Unit transposon | 86 | AB238971.1 | Metabolism | (191) |
| Tn*4658* | *Pseudomonas putida* | Unit transposon | 5.7 | AB238971.1 | - | (191) |
| Tn*4659* | *Pseudomonas putida* | Unit transposon | 3.7 | AB238971.1 | - | (191) |
| Tn*4660* | *Pseudomonas putida* | Unit transposon | 62 | AB238971.1 | Metabolism | (191) |
| Tn*4661* | *Pseudomonas aeruginosa* | Unit transposon | 13 | AB375440.1 | - | (192) |
| Tn*4671* | *Delftia acidovorans* | Unit transposon | 8.4 | AB063332.1 | Heavy metal resistance | (193) |
| Tn*4672* | *Delftia acidovorans* | Unit transposon | 11.9 | AB063332.1 | Heavy metal resistance | (193) |
| Tn*4676* | *Pseudomonas resinovorans* | Unit transposon | 73 | AB088420.3 | Metabolism | (194) |
| Tn*4731* | *Escherichia coli* | Composite transposon | - | - | AMR | (195) |
| Tn*4811* | *Streptomyces lividans* | Unit transposon | 5.4 | Z11519.1 | - | (196) |
| Tn*5030* | *Bacteroides sp.* | Conjugative transposon | 45 | - | AMR | (197) |
| Tn*5031* | *Enterococcus faecium* | Conjugative transposon | - | - | AMR | (198) |
| Tn*5032* | *Enterococcus faecium* | Conjugative transposon | - | - | AMR | (198) |
| Tn*5033* | *Enterococcus faecium* | Conjugative transposon | - | - | AMR | (198) |
| Tn*5036* | *Enterobacter cloacae* | Unit transposon | 8 | Y09025.1 | Heavy metal resistance | (199) |
| Tn*5037* | *Thiobacillus ferrooxidans* | Unit transposon | 6.6 | AJ251743.1 | Heavy metal resistance | (200) |
| Tn*5041* | *Pseudomonas sp.* | Unit transposon | 14.9 | X98999.3 | Heavy metal resistance | (201) |
| Tn*5042* | *Pseudomonas fluorescens* | Unit transposon | 7 | AJ563380.2 | Heavy metal resistance | (202) |
| Tn*5044* | *Xanthomonas campestris* | Unit transposon | 10 | Y17691.1 | Heavy metal resistance | (203) |
| Tn*5045* | *Pseudomonas sp.* | Unit transposon | 22 | FN821089.1 | AMR, Heavy metal resistance | (204) |
| Tn*5046* | *Pseudomonas sp.* | Unit transposon | 10 | Y18360.1 | Heavy metal resistance | (205) |
| Tn*5053* | *Xanthomonas sp.* | Unit transposon | 8.4 | L40585.1 | Heavy metal resistance | (206) |
| Tn*5056* | *Escherichia coli* | Unit transposon | - | AJ302763.1, AJ302764.1, AJ302765.1 | Heavy metal resistance | (202) |
| Tn*5058* | *Pseudomonas sp.* | Unit transposon | 12 | Y17897.1 | Heavy metal resistance | (207) |
| Tn*5060* | *Pseudomonas sp.* | Unit transposon | 8.7 | AJ551280.1 | Heavy metal resistance | (208) |
| Tn*5070* | *Pseudomonas sp.* | Unit transposon | 7 | Y17830.1 | Heavy metal resistance | (205) |
| Tn*5073* | *Klebsiella pneumoniae* | Unit transposon | 4.3 | AF461013.1 | Heavy metal resistance | (209) |
| Tn*5074* | *Morganella morganii* | Unit transposon | - | AF461012.1 | Heavy metal resistance | (209) |
| Tn*5075* | *Escherichia coli* | Composite transposon | 11.2 | AF457211.1 | Heavy metal resistance | (209) |
| Tn*5083* | *Bacillus megaterium* | Unit transposon | 5.5 | Y18009.2, Y09907.1 | Heavy metal resistance | (210) |
| Tn*5084* | *Bacillus cereus* | Unit transposon | 11.5 | AB066362.1 | Heavy metal resistance | (210) |
| Tn*5085* | *Exiguobacterium sp.* | Unit transposon | 11.8 | Y17750.1, Y17751.1, Y17752.1 | Heavy metal resistance | (210) |
| Tn*5086* | *Escherichia coli* | Unit transposon | 15.3 | X58425.1 | AMR, Heavy metal resistance | (211) |
| Tn*5090* | *Klebsiella aerogenes* | Unit transposon | 7.5 | X72585.1 | AMR, Antiseptic resistance | (212) |
| Tn*5096* | *Streptomyces spp.* | Unit transposon | 3 | - | AMR | (213) |
| Tn*5097* | *Streptomyces spp.* | Unit transposon | 3 | - | AMR | (214) |
| Tn*5098* | *Streptomyces fradiae* | Unit transposon | 5.3 | - | AMR, Metabolism | (215) |
| Tn*5099* | *Streptomyces spp.* | Unit transposon | 4.4 | - | AMR | (216) |
| Tn*5251* | *Streptococcus pneumoniae* | Conjugative transposon | 18 | FJ711160.1 | AMR | (217) |
| Tn*5252* | *Streptococcus pneumoniae* | Conjugative transposon | 47.5 | - | AMR | (218) |
| Tn*5253* | *Streptococcus pneumoniae* | Conjugative transposon | 66 | EU351020.1 | AMR | (219) |
| Tn*5271* | *Alcaligenes sp.* | Composite transposon | 17 | - | Metabolism | (220) |
| Tn*5276* | *Lactococcus lactis* | Conjugative transposon | 70 | CP001834.1 | Virulence determinants, Metabolism | (221) |
| Tn*5301* | *Lactococcus lactis* | Conjugative transposon | 70 | - | Virulence determinants, Metabolism | (222) |
| Tn*5306* | *Lactococcus lactis* | Conjugative transposon | - | - | Virulence determinants, Metabolism | (223) |
| Tn*5381* | *Enterococcus faecalis* | Conjugative transposon | 19 | - | AMR | (224) |
| Tn*5382* | *Enterococcus faecium* | Conjugative transposon | 27 | AF063010.1, AF063900.1 | AMR | (225) |
| Tn*5383* | *Enterococcus faecalis* | Conjugative transposon | 19 | - | AMR | (224) |
| Tn*5384* | *Enterococcus faecalis* | Composite transposon | 26 | - | AMR | (226) |
| Tn*5385* | *Enterococcus faecalis* | Composite transposon | 65 | - | AMR, Heavy metal resistance | (227) |
| Tn*5386* | *Enterococcus faecium* | Conjugative transposon | 29 | DQ321786.1 | AMR | (228) |
| Tn*5393* | *Erwinia amylovora* | Unit transposon | 6.7 | M96392.1 | AMR | (229) |
| Tn*5394* | *Erwinia pyrifoliae* | Unit transposon | 6.3 | AY123045.1 | - | (230) |
| Tn*5396* | *Escherichia coli* | Unit transposon | - | - | - | (231) |
| Tn*5397* | *Clostridium difficile* | Conjugative transposon | 21 | AF333235.1 | AMR | (232) |
| Tn*5401* | *Bacillus thuringiensis* | Unit transposon | 4.8 | U03554.1 | - | (233) |
| Tn*5403* | *Klebsiella pneumoniae* | Unit transposon | 3.6 | X75779.1 | - | (234) |
| Tn*5404* | *Staphylococcus aureus* | Unit transposon | 16 | L43098.1 | AMR | (235) |
| Tn*5405* | *Staphylococcus aureus* | Composite transposon | 12 | U73025.1, U73026.1, U73027.1 | AMR | (236) |
| Tn*5406* | *Staphylococcus aureus* | Unit transposon | 5.5 | JQ319423.1 | AMR | (237) |
| Tn*5422* | *Listeria monocytogenes* | Unit transposon | 6.5 | L28104.1 | Heavy metal resistance | (238) |
| Tn*5431* | *Escherichia coli* | Unit transposon | 16 | X87813.1, X87814.1 | AMR | (239) |
| Tn*5432* | *Cutibacterium acnes* | Composite transposon | 4.5 | AF411029.1 | AMR | (240) |
| Tn*5466* | *Enterococcus hirae* | Composite transposon | 8.8 | X81655.1 | AMR | (241) |
| Tn*5467* | *Thiobacillus ferrooxidans* | Unit transposon | 3.5 | L37364.1 | - | (242) |
| Tn*5468* | *Thiobacillus ferrooxidans* | Unit transposon | 8 | AF032884.1 | - | (243) |
| Tn*5469* | *Fremyella diplosiphon* | Unit transposon | 4.9 | U33002.1 | - | (244) |
| Tn*5481* | *Lactococcus lactis* | Unit transposon | 5 | AJ000993.1 | - | (245) |
| Tn*5491* | *Streptomyces lividans* | Unit transposon | - | - | AMR | (246) |
| Tn*5492* | *Streptomyces lividans* | Unit transposon | - | - | AMR | (247) |
| Tn*5493* | *Streptomyces lividans* | Unit transposon | - | - | AMR | (248) |
| Tn*5495* | *Pseudomonas veronii* | Unit transposon | - | - | AMR | (249) |
| Tn*5501* | *Pseudomonas putida* | Unit transposon | 5.6 | Y09450.1 | - | (250) |
| Tn*5502* | *Pseudomonas putida* | Unit transposon | 9 | Y09450.1, AF052749.1 | - | (250) |
| Tn*5506* | *Enterococcus faecium* | Composite transposon | 39 | L40841.1 | AMR | (251) |
| Tn*5520* | *Bacteroides fragilis* | Unit transposon | 4.7 | AF038866.2 | - | (252) |
| Tn*5530* | *Burkholderia cepacia* | Composite transposon | 36 | AF029344.2 | Metabolism, Efflux functions | (253) |
| Tn*5531* | *Corynebacterium glutamicum* | Composite transposon | 4.5 | U53587.1 | AMR | (254) |
| Tn*5541* | *Fremyella diplosiphon* | Unit transposon | 4.7 | AF072896.1 | - | (255) |
| Tn*5542* | *Pseudomonas putida* | Composite transposon | 11.9 | AF148496.1 | Metabolism | (256) |
| Tn*5561* | *Rhodococcus erythropolis* | Unit transposon | - | AF002247.1 | - | (257) |
| Tn*5563* | *Pseudomonas alcaligenes* | Unit transposon | 6.3 | NC_005909.1 | Efflux function | (258) |
| Tn*5564* | *Corynebacterium glutamicum* | Unit transposon | 4.2 | AF024666.2 | AMR | (259) |
| Tn*5565* | *Clostridium perfringens* | Composite transposon | 6.3 | - | Virulence determinants | (260) |
| Tn*5613* | *Acinetobacter sp.* | Composite transposon | 3.5 | AF091240.1 | - | (261) |
| Tn*5706* | *Pasteurella multocida* | Composite transposon | 4.4 | Y15510.1 | AMR | (262) |
| Tn*5707* | *Cupriavidus necator* | Composite transposon | 15 | D64144.1, AB019032.1 | Metabolism | (263) |
| Tn*5718* | Uncultured bacterium | Unit transposon | 10 | AJ304453.1 | Heavy metal resistance | (264) |
| Tn*5719* | Uncultured bacterium | Unit transposon | 8.4 | AJ431260.1 | Heavy metal resistance | (265) |
| Tn*5721* | *Lactococcus lactis* | Composite transposon | 11.5 | U91581.1 | Virulence determinants | (266) |
| Tn*5801* | *Staphylococcus aureus* | Conjugative transposon | 25.8 | BA000017.4 | AMR | (267) |
| Tn*6000* | *Enterococcus casseliflavus* | Conjugative transposon | 33.3 | FN555436.1 | AMR | (268) |
| Tn*6001* | *Pseudomonas aeruginosa* | Unit transposon | 12.1 | EF138817.1 | AMR | (269) |
| Tn*6002* | *Streptococcus cristatus* | Conjugative transposon | 20.9 | AY898750.1 | AMR | (270) |
| Tn*6003* | *Streptococcus pneumoniae* | Conjugative transposon | 25.1 | AM410044.5 | AMR | (271) |
| Tn*6004* | *Acinetobacter baumannii* | Unit transposon | - | - | AMR | (272) |
| Tn*6005* | *Enterobacter cloacae* | Unit transposon | 23.7 | EU591509.1 | Antiseptic resistance, Heavy metal resistance | (273) |
| Tn*6006* | *Enterobacter cloacae* | Unit transposon | 15.7 | EU591509.1 | Antiseptic resistance | (273) |
| Tn*6007* | *Enterobacter cloacae* | Unit transposon | 7.2 | EU591509.1 | Antiseptic resistance | (273) |
| Tn*6008* | *Enterobacter cloacae* | Unit transposon | 6 | EU591509.1 | - | (273) |
| Tn*6009* | *Klebsiella pneumoniae* | Conjugative transposon | 19.7 | EU239355.1, EU399632.1 | AMR, Heavy metal resistance | (274) |
| Tn*6010* | *Escherichia coli* | Composite transposon | 5.1 | EU370913.1 | AMR | (275) |
| Tn*6011* | *Escherichia coli* | Composite transposon | 5.6 | EU370913.1 | Virulence determinants | (275) |
| Tn*6012* | *Staphylococcus aureus* | Conjugative transposon | 13.3 | AB435014.1 | - | (276) |
| Tn*6013* | *Staphylococcus aureus* | Conjugative transposon | 13.4 | FJ231270.1 | AMR | (277) |
| Tn*6014* | *Staphylococcus aureus* | Conjugative transposon | - | EU918655.2 | AMR | (278) |
| Tn*6016* | *Pseudomonas aeruginosa* | Unit transposon | 8.8 | KC543497.1 | AMR, Heavy metal resistance | (279) |
| Tn*6017* | *Citrobacter freundii* | Unit transposon | 9 | CP016763.1 | AMR, Heavy metal resistance | (280) |
| Tn*6018* | *Acinetobacter baumannii* | Unit transposon | 3.4 | FJ172370.5 | Heavy metal resistance | (281) |
| Tn*6019* | *Acinetobacter baumannii* | Unit transposon | 59 | FJ172370.5 | Heavy metal resistance | (282) |
| Tn*6020* | *Acinetobacter baumannii* | Composite transposon | 3 | FJ172370.5 | AMR | (281) |
| Tn*6021* | *Acinetobacter baumannii* | Unit transposon | 13.1 | CP000521.1 | - | (282) |
| Tn*6022* | *Acinetobacter baumannii* | Unit transposon | 12 | JN107991.2 | - | (283) |
| Tn*6023* | *Salmonella enterica* | Composite transposon | 2.6 | GU562437.2 | AMR | (284) |
| Tn*6024* | *Serratia marcescens* | Unit transposon | 32.4 | BX664015.1 | Heavy metal resistance | - |
| Tn*6025* | *Salmonella enterica* | Unit transposon | 13.3 | GU562437.2 | AMR, Heavy metal resistance | (285) |
| Tn*6026* | *Salmonella enterica* | Composite transposon and Unit transposon | 24.3 | GQ150541.1 | AMR, Heavy metal resistance | (285) |
| Tn*6027* | *Salmonella enterica* | Composite transposon | 28 | HQ840942.1 | AMR | (286) |
| Tn*6028* | *-* | Unit transposon | - | - | AMR | - |
| Tn*6029* | *Salmonella enterica* | Composite transposon | 8.8 | GQ150541.1 | AMR | (285) |
| Tn*6030* | *Hydrogenobacter thermophilus* | Unit transposon | 30.9 | AP011112.1 | - | (287) |
| Tn*6031* | *Sphingobacterium sp.* | Unit transposon | 13 | EU864422.1 | AMR | (288) |
| Tn*6032* | Uncultured bacterium | Unit transposon | 3.3 | AB266126.2 | - | (289) |
| Tn*6033* | *Ralstonia pickettii* | Conjugative transposon | 54.1 | CP001068.1 | - | (290) |
| Tn*6034* | *Shewanella sp.* | Conjugative transposon | 45.2 | NC_008577.1 | - | (290) |
| Tn*6035* | *Congregibacter litoralis* | Conjugative transposon | 50.7 | NZ_AAOA01000008.1 | - | (290) |
| Tn*6036* | *Acidovorax avenae* | Conjugative transposon | 60 | NC_008752.1 | - | (290) |
| Tn*6037* | *Delftia acidovorans* | Conjugative transposon | 57.9 | NC_010002.1 | - | (290) |
| Tn*6038* | *Comamonas testosteroni* | Conjugative transposon | 52.5 | - | - | (290) |
| Tn*6039* | *Acidovorax sp.* | Conjugative transposon | 53.5 | NC_008782.1 | - | (290) |
| Tn*6040* | *Bordetella petrii* | Conjugative transposon | 47.1 | NC_010170.1 | - | (290) |
| Tn*6041* | *Pseudomonas aeruginosa* | Conjugative transposon | 48.5 | NZ_AAKW01000024.1 | - | (290) |
| Tn*6042* | *Pseudomonas aeruginosa* | Conjugative transposon | 55.3 | NC_009656.1 | - | (290) |
| Tn*6045* | *Macrococcus caseolyticus* | Unit transposon | 6.7 | AB498756.1 | AMR | (291) |
| Tn*6048* | *Cupriavidus metallidurans* | Unit transposon | 10.4 | CP000353.2 | - | (292) |
| Tn*6049* | *Cupriavidus metallidurans* | Unit transposon | 3.5 | CP000353.2 | - | (292) |
| Tn*6050* | *Cupriavidus metallidurans* | Unit transposon | 6.8 | CP000353.2 | - | (292) |
| Tn*6051* | *Delftia acidovorans* | Unit transposon | - | - | - | (292) |
| Tn*6052* | *Burkholderia xenovorans* | Unit transposon | - | - | - | (292) |
| Tn*6058* | *Streptococcus pneumoniae* | Conjugative transposon | 67.8 | FM201786.3 | AMR | (293) |
| Tn*6060* | *Pseudomonas aeruginosa* | Composite transposon | 25.4 | GQ161847.1 | AMR | (294) |
| Tn*6061* | *Pseudomonas aeruginosa* | Unit transposon | 26.6 | GU475047.1, GU475048.1, GU475049.1, GU475053.1 | AMR | (295) |
| Tn*6062* | *Salmonella enterica* | Composite transposon | 4.9 | AM412236.1 | Efflux function | (296) |
| Tn*6063* | Delftia acidovorans | Composite transposon | 22.4 | JF274990.2 | Metabolism | (297) |
| Tn*6072* | *Staphylococcus aureus* | Composite transposon | 29.4 | GU235985.1 | AMR | (298) |
| Tn*6073* | *Clostridium difficile* | Conjugative transposon | 29.1 | BK008006.1 | Efflux function | (299) |
| Tn*6074* | *Salmonella bongori* | Conjugative transposon | 109 | FN298494.1 | AMR, Efflux function | (300) |
| Tn*6075* | *Salmonella enterica* | Conjugative transposon | 104 | FN298495.1 | - | (300) |
| Tn*6076* | *Salmonella Senftenberg* | Conjugative transposon | 114 | FN298496.1 | Metabolism | (300) |
| Tn*6077* | *Yersinia enterocolitica* | Conjugative transposon | 120 | FN298493.1 | Efflux function | (300) |
| Tn*6079* | Uncultured bacterium | Conjugative transposon | 28.4 | GU951538.1 | AMR | (301) |
| Tn*6080* | *Acinetobacter baumannii* | Unit transposon | 3.2 | GQ352402.1 | AMR | (302) |
| Tn*6082* | *Salmonella enterica* | Unit transposon | 3.6 | AP005147.1 | AMR | (303) |
| Tn*6083* | *Salmonella bongori* | Conjugative transposon | 84 | FN669609.1 | AMR | (300) |
| Tn*6084* | *Enterococcus faecium* | Conjugative transposon | 22.3 | HM243622.1 | AMR | (304) |
| Tn*6085* | *Enterococcus faecium* | Conjugative transposon | 20.8 | HM243621.1, HM243623.1 | AMR | (304) |
| Tn*6086* | *Enterococcus faecium* | Conjugative transposon | 24.5 | HM636636.1 | AMR | - |
| Tn*6087* | *Streptococcus oralis* | Conjugative transposon | 21.1 | HQ663849.2 | AMR, Antiseptic resistance | (305) |
| Tn*6088* | *Salmonella enterica* | Composite transposon and Unit transposon | 43.3 | AY509004.1 | AMR, Antiseptic resistance, Efflux functions,  Heavy metal resistance | (306) |
| Tn*6089* | *Salmonella enterica* | Composite transposon | 3 | AY509004.1 | AMR | (306) |
| Tn*6090* | *Salmonella enterica* | Composite transposon | 6 | AY509004.1 | - | (306) |
| Tn*6091* | *Salmonella enterica* | Composite transposon | 6.5 | AY509004.1 | - | (306) |
| Tn*6092* | *Salmonella enterica* | Unit transposon | 4.1 | AY509004.1 | AMR, Efflux function | (306) |
| Tn*6093* | *Proteus mirabilis* | Unit transposon | 6.3 | FM995219.1 | AMR, Antiseptic resistance, Efflux functions | (307) |
| Tn*6097* | *Paracoccus ferrooxidans* | Composite transposon | 17.8 | JN122276.1 | AMR, Metabolism | (308) |
| Tn*6098* | *Lactococcus lactis* | Conjugative transposon | 51 | CP001834.1 | Efflux function, Metabolism, Virulence determinants | (309) |
| Tn*6099* | *Prevotella nigrescens* | Unit transposon | 7 | HM561907.1 | AMR | (310) |
| Tn*6100* | *Prevotella nigrescens* | Unit transposon | 6.3 | HM561908.1 | AMR | (310) |
| Tn*6101* | *Salmonella enterica* | Conjugative transposon | 84 | FR686852.1 | - | (300) |
| Tn*6103* | *Clostridium difficile* | Conjugative transposon | 84.9 | BK008007.1 | Efflux function | (299) |
| Tn*6104* | *Clostridium difficile* | Conjugative transposon | 15.6 | FN545816.1 | Efflux function | (299) |
| Tn*6105* | *Clostridium difficile* | Conjugative transposon | 15.8 | FN545816.1 | - | (299) |
| Tn*6106* | *Clostridium difficile* | Conjugative transposon | 11.3 | FN545816.1 | - | (299) |
| Tn*6107* | *Clostridium difficile* | Conjugative transposon | 50.5 | BK008008.1 | Efflux function | (299) |
| Tn*6108* | *Salmonella enterica* | Composite transposon | 31 | JF274992.1 | AMR, Heavy metal resistance | - |
| Tn*6109* | *Salmonella enterica* | Composite transposon | 23 | JF274992.1 | - | - |
| Tn*6110* | *Clostridium difficile* | Conjugative transposon | 58 | BK008009.1 | - | (299) |
| Tn*6111* | *Clostridium difficile* | Conjugative transposon | 53.4 | JF422669.1 | - | (299) |
| Tn*6112* | *Pseudomonas sp.* | Unit transposon | 7.3 | HQ423158.1 | - | (311) |
| Tn*6113* | *Citrobacter freundii* | Unit transposon | - | FR716828.1 | AMR, Efflux function | (312) |
| Tn*6114* | *Klebsiella pneumoniae* | Unit transposon | 4 | EU331426.1 | AMR, Efflux function | (312) |
| Tn*6115* | *Clostridium difficile* | Unit transposon | 13.6 | - | Efflux function | (299) |
| Tn*6122* | *Paracoccus halophilus* | Unit transposon | 3.8 | JN127372.1 | - | (308) |
| Tn*6125* | *Delftia acidovorans* | Composite transposon | 32.8 | HQ891317.1 | Metabolism | - |
| Tn*6126* | *Delftia acidovorans* | Composite transposon | 35.9 | JF274988.1 | Metabolism | (313) |
| Tn*6127* | *Comamonas testosteroni* | Composite transposon | 37.8 | JF274987.1 | Metabolism | (297) |
| Tn*6130* | *Comamonas testosteroni* | Unit transposon | 15.6 | JF274989.1 | Heavy metal resistance | (313) |
| Tn*6133* | *Staphylococcus aureus* | Unit transposon | 11.5 | FR772051.1 | AMR | (314) |
| Tn*6134* | *Sphingobium japonicum* | Unit transposon | 4.6 | AB610645.1 | Metabolism | (315) |
| Tn*6135* | *Sphingobium japonicum* | Unit transposon | 4.3 | AB610646.1 | - | (315) |
| Tn*6136* | *Sphingobium japonicum* | Unit transposon | 3.8 | AB610647.1 | - | (315) |
| Tn*6137* | *Sphingobium japonicum* | Unit transposon | 3.8 | AB610648.1 | - | (315) |
| Tn*6138* | *Sphingobium japonicum* | Unit transposon | 4.6 | AB610649.1 | Metabolism | (315) |
| Tn*6161* | *Porphyromonas gingivalis* | Conjugative transposon | 44.3 | AP009380.1 | Efflux functions | (316) |
| Tn*6162* | *Pseudomonas aeruginosa* | Composite transposon and Unit transposon | 26 | JF826498.1 | AMR, Heavy metal resistance | (317) |
| Tn*6163* | *Pseudomonas aeruginosa* | Unit transposon | 21.2 | JF826499.1 | AMR, Heavy metal resistance | (317) |
| Tn*6164* | *Clostridium difficile* | Conjugative transposon | 106 | FN665653.1 | AMR | (318) |
| Tn*6166* | *Acinetobacter baumannii* | Unit transposon | 17.6 | JN247441.4 | AMR | (319) |
| Tn*6167* | *Acinetobacter baumannii* | Composite transposon and Unit transposon | 33 | JN968483.3 | AMR | (320) |
| Tn*6168* | *Acinetobacter baumannii* | Composite transposon | 5.5 | KC118540.6 | AMR | (321) |
| Tn*6170* | *Escherichia coli* | Unit transposon | 18.8 | AP012208.1 | - | (322) |
| Tn*6171* | *Acinetobacter baumannii* | Unit transposon | 49.9 | CP012952.1 | Metabolism | (323) |
| Tn*6172* | *Acinetobacter baumannii* | Unit transposon | 11.7 | KU744946.1, KT779035.1 | AMR | (324) |
| Tn*6176* | *Acinetobacter baumannii* | Unit transposon | 3.4 | KU549175.1 | Heavy metal resistance, Virulence determinants | - |
| Tn*6177* | *Acinetobacter baumannii* | Unit transposon | 35.6 | MG954377.1 | Heavy metal resistance | - |
| Tn*6178* | *Acinetobacter baumannii* | Composite transposon | 4.6 | KP054476.2 | AMR, Efflux function | - |
| Tn*6179* | *Acinetobacter baumannii* | Composite transposon | 2.7 | KX011025.2 | AMR | (325) |
| Tn*6180* | *Acinetobacter baumannii* | Composite transposon | 19 | KX011025.2 | AMR | (325) |
| Tn*6181* | *Escherichia coli* | Unit transposon | 19.4 | KX242350.1 | AMR | - |
| Tn*6182* | *Pseudomonas aeruginosa* | Unit transposon | 11.5 | KX709966.1 | Heavy metal resistance | (326) |
| Tn*6183* | *Acinetobacter baumannii* | Composite transposon and Unit transposon | 25.8 | MF399199.1 | AMR | - |
| Tn*6186* | *Bacteroides fragilis* | Composite transposon | 8.5 | AB646744.1 | AMR, Efflux function | (327) |
| Tn*6187* | *Klebsiella pneumoniae* | Composite transposon and Unit transposon | 32.6 | JQ010984.1 | AMR, Heavy metal resistance | (328) |
| Tn*6188* | *Listeria monocytogenes* | Unit transposon | 5.1 | HG329628.1 | Antiseptic resistance | (329) |
| Tn*6190* | *Clostridium difficile* | Conjugative transposon | 17.9 | FN665653.1 | AMR | (318) |
| Tn*6191* | *Staphylococcus haemolyticus* | Composite transposon | 6.3 | JQ764731.1 | AMR | (330) |
| Tn*6192* | *Clostridium difficile* | Conjugative transposon | 37.5 | FN545816.1 | Efflux function | (331) |
| Tn*6194* | *Clostridium difficile* | Conjugative transposon | 28 | HG475346.1 | AMR | (332) |
| Tn*6196* | *Escherichia coli* | Unit transposon | 3.6 | KC999035.4 | - | (333) |
| Tn*6198* | *Listeria monocytogenes* | Conjugative transposon | 21.3 | JX120102.1 | AMR | (334) |
| Tn*6202* | *Enterococcus faecalis* | Conjugative transposon | 43.7 | FJ872411.1 | AMR | (335) |
| Tn*6203* | *Achromobacter xylosoxidans* | Unit transposon | 17.5 | JX448550.1 | AMR, Heavy metal resistance | (336) |
| Tn*6204* | *Mycobacterium abscessus* | Unit transposon | 11.5 | CP003505.2 | AMR | - |
| Tn*6205* | *Mycobacterium abscessus* | Unit transposon | 2.8 | CP003505.2 | AMR | - |
| Tn*6206* | *Acinetobacter baumannii* | Composite transposon | 7.8 | CP003849.1 | AMR | (337) |
| Tn*6207* | *Acinetobacter baumannii* | Unit transposon | 6.8 | CP001937.2 | AMR | (337) |
| Tn*6208* | *Acinetobacter baumannii* | Composite transposon | 9.2 | CP003846.1 | AMR | (337) |
| Tn*6209* | *Acinetobacter baumannii* | Unit transposon | 8.4 | CP003849.1 | AMR | (337) |
| Tn*6210* | *Acinetobacter baumannii* | Composite transposon | - | - | AMR | (337) |
| Tn*6211* | *Pseudomonas syringae* | Unit transposon | 4.9 | KX009065.1 | - | (338) |
| Tn*6212* | *Pseudomonas syringae* | Not specified | 16.3 | - | Heavy metal resistance, Efflux function | (338) |
| Tn*6213* | *Pseudomonas syringae* | Unit transposon | 4.8 | - | - | (338) |
| Tn*6214* | *Escherichia coli* | Unit transposon | 29 | KJ541071.1 | AMR, Heavy metal resistance | (339) |
| Tn*6215* | *Clostridioides difficile* | Conjugative transposon | 13 | KC166248.1 | AMR | (340) |
| Tn*6216* | *Enterobacter cloacae* | Unit transposon | 14 | KC511628.1 | AMR | (341) |
| Tn*6217* | *Pseudomonas aeruginosa* | Unit transposon | 6.9 | KC543497.1 | AMR | (279) |
| Tn*6218* | *Clostridium difficile* | Unit transposon | 14.2 | HG002387.1 | AMR | (342) |
| Tn*6222* | *Escherichia coli* | Composite transposon | 7.8 | CP003297.1 | AMR | (343) |
| Tn*6223* | *Escherichia coli* | Composite transposon | 1.5 | CP003289.1 | - | (343) |
| Tn*6224* | *Lactobacillus salivarius* | Conjugative transposon | 17 | CP007650.1 | AMR | (344) |
| Tn*6227* | *Streptococcus mitis* | Unit transposon | 7.9 | KJ690251.1 | AMR | - |
| Tn*6228* | *Sphingomonas sp.* | Composite transposon | 32.5 | KF494257.1 | Metabolism | (345) |
| Tn*6229* | *Klebsiella pneumoniae* | Unit transposon | 16.8 | KF295829.1 | AMR | (346) |
| Tn*6230* | *Salmonella enterica* | Unit transposon | 37 | - | Heavy metal resistance | (347) |
| Tn*6231* | *Pseudomonas moraviensis* | Unit transposon | - | - | - | (348) |
| Tn*6233* | *Burkholderia sp.* | Unit transposon | 8.8 | AB853026.1 | Heavy metal resistance | (349) |
| Tn*6234* | *Klebsiella pneumoniae* | Unit transposon | 29 | HG934082.1 | AMR, Heavy metal resistance | (350) |
| Tn*6235* | *Clostridium difficile* | Not specified | 40 | - | AMR | (351) |
| Tn*6237* | *Escherichia coli* | Composite transposon | 21.9 | - | AMR | (352) |
| Tn*6238* | *Klebsiella pneumoniae* | Unit transposon | 8 | KJ511462.1 | AMR | (353) |
| Tn*6240* | *Klebsiella pneumoniae* | Unit transposon | 9.6 | HG934082.1 | AMR | (350) |
| Tn*6242* | *Escherichia coli* | Composite transposon | 19.7 | - | AMR | (354) |
| Tn*6246* | *Enterococcus faecium* | Composite transposon | 5.1 | KP834591.1 | AMR | - |
| Tn*6247* | *Enterococcus faecium* | Conjugative transposon | 23.7 | KP345886.1 | AMR | - |
| Tn*6248* | *Enterococcus faecium* | Conjugative transposon | 26 | KP834592.1 | AMR | (355) |
| Tn*6249* | *Pseudomonas aeruginosa* | Composite transposon and Unit transposon | 26 | LK054503.1 | AMR | (356) |
| Tn*6250* | *Acinetobacter baumannii* | Composite transposon | 12.8 | CP007712.1 | AMR | (357) |
| Tn*6251* | *Acinetobacter baumannii* | Composite transposon | 5.2 | CP007712.1 | - | (357) |
| Tn*6252* | *Acinetobacter baumannii* | Composite transposon | 3.2 | CP007712.1 | AMR | (357) |
| Tn*6253* | *Streptococcus pyogenes* | Conjugative transposon | 20.2 | CP007024.1 | AMR | (358) |
| Tn*6254* | *Lactobacillus hokkaidonensis* | Conjugative transposon | 52 | AP014680.1 | Heavy metal resistance, Metabolism | (359) |
| Tn*6255* | *Citrobacter freundii* | Unit transposon | 4 | KP851978.1 | AMR | (360) |
| Tn*6256* | *Citrobacter freundii* | Composite transposon | 12.9 | KP851978.1 | AMR | (360) |
| Tn*6260* | *Enterococcus faecalis* | Unit transposon | 4.5 | KX470419.1 | AMR | (361) |
| Tn*6261* | *Enterococcus faecalis* | Unit transposon | 8.9 | KU354267.1 | AMR | (362) |
| Tn*6263* | *Streptococcus gallolyticus* | Conjugative transposon | 46 | JYKU01000013.1 | AMR | (363) |
| Tn*6264* | *Vibrio parahaemolyticus* | Composite transposon | 5.4 | KM067908.1 | Virulence determinants | (364) |
| Tn*6268* | *Sphingobium sp.* | Unit transposon | 4.1 | LC102249.1 | - | (365) |
| Tn*6269* | *Sphingobium sp.* | Unit transposon | 4.1 | LC102250.1 | - | (365) |
| Tn*6270* | *Sphingobium sp.* | Unit transposon | 4.2 | LC102251.1 | - | (365) |
| Tn*6271* | *Sphingobium sp.* | Unit transposon | 3.7 | LC102252.1 | - | (365) |
| Tn*6272* | *Sphingobium sp.* | Unit transposon | 3.8 | LC102253.1 | - | (365) |
| Tn*6273* | *Sphingobium sp.* | Unit transposon | 2.4 | LC102254.1 | - | (365) |
| Tn*6274* | *Sphingobium sp.* | Unit transposon | 4.6 | LC102255.1 | - | (365) |
| Tn*6275* | *Sphingobium sp.* | Unit transposon | 3.8 | LC102256.1 | - | (365) |
| Tn*6276* | *Sphingobium sp.* | Unit transposon | 4.6 | LC102257.1 | - | (365) |
| Tn*6277* | *Sphingobium sp.* | Unit transposon | 5.3 | LC102258.1 | - | (365) |
| Tn*6278* | *Sphingobium sp.* | Unit transposon | 3.9 | LC102259.1 | - | (365) |
| Tn*6279* | *Acinetobacter baumannii* | Composite transposon | 21 | KT317075.1 | AMR | (366) |
| Tn*6283* | *Vibrio sp.* | Unit transposon | 13.8 | LC081338.1 | Virulence determinants | (367) |
| Tn*6284* | *Pseudomonas aeruginosa* | Unit transposon | 22.6 | KU254577.1 | AMR, Heavy metal resistance | (368) |
| Tn*6285* | *Shigella boydii* | Composite transposon and Unit transposon | 25.8 | KX646543.1 | AMR, Antiseptic resistance, Heavy metal resistance | (368) |
| Tn*6286* | *Pseudomonas putida* | Unit transposon | 23.7 | KU130294.1 | AMR | (369) |
| Tn*6290* | *Pseudomonas putida* | Not specified | 42 | BK010246.1 | - | (370) |
| Tn*6291* | *Pseudomonas fluorescens* | Not specified | 22.3 | BK010245.1 | - | (370) |
| Tn*6292* | *Pseudomonas aeruginosa* | Unit transposon | - | - | AMR | (371) |
| Tn*6293* | *Clostridium difficile* | Conjugative transposon | 104 | ERS1242839 | AMR | (372) |
| Tn*6294* | *Paenibacillus sp.* | Unit transposon | 8.6 | LC015492.1 | Heavy metal resistance | (373) |
| Tn*6295* | *Shigella boydii* | Composite transposon | 6.1 | KX646543.1 | AMR | (374) |
| Tn*6296* | *Klebsiella pneumoniae* | Unit transposon | 14.3 | FJ628167.2 | AMR | (375) |
| Tn*6297* | *Shewanella xiamenensis* | Composite transposon and Unit transposon | 78.2 | CP013115.1 | AMR, Antiseptic resistance,  Heavy metal resistance | (376) |
| Tn*6298* | Uncultured bacterium | Unit transposon | 3 | KU736866.1 | AMR | (377) |
| Tn*6299* | Uncultured bacterium | Unit transposon | 21.4 | KU736875.1 | AMR | (377) |
| Tn*6300* | Uncultured bacterium | Unit transposon | 7.2 | KU736876.1 | AMR | (377) |
| Tn*6301* | Uncultured bacterium | Composite transposon and Unit transposon | 12.1 | KU736877.1 | AMR | (377) |
| Tn*6302* | Uncultured bacterium | Composite transposon | 11 | KU736878.1 | AMR | (377) |
| Tn*6303* | Uncultured bacterium | Unit transposon | 4.6 | KU736874.1 | AMR | (377) |
| Tn*6306* | *Raoultella ornithinolytica* | Composite transposon | 13.8 | KT225520.1 | AMR | (378) |
| Tn*6308* | *Leclercia adecarboxylata* | Composite transposon and Unit transposon | 30.6 | KX710094.1 | AMR, Heavy metal resistance | (379) |
| Tn*6309* | *Leclercia adecarboxylata* | Composite transposon | 5.4 | KX710094.1 | AMR | (379) |
| Tn*6310* | *Vibrio cholerae* | Unit transposon | 8.2 | AXDR01000001.1 | Heavy metal resistance | (380) |
| Tn*6317* | *Leclercia adecarboxylata* | Composite transposon | 21 | KX710093.1 | Heavy metal resistance | (379) |
| Tn*6320* | *Enterobacter cloacae* | Composite transposon and Unit transposon | 8.4 | MF062700.1 | AMR | - |
| Tn*6321* | *Enterobacter cloacae* | Unit transposon | 21.3 | KY270852.1 | AMR, Antiseptic resistance, Heavy metal resistance | - |
| Tn*6322* | *Leclercia adecarboxylata* | Unit transposon | 9.3 | KX710093.1 | Heavy metal resistance | (379) |
| Tn*6325* | *Enterobacter hormaechei* | Unit transposon | 23.8 | CP010378.1 | AMR, Heavy metal resistance | (381) |
| Tn*6326* | *Actinotignum schaalii* | Composite transposon | 4 | KT897470.1 | AMR | (382) |
| Tn*6329* | *Klebsiella pneumoniae* | Composite transposon | 18 | LT576116.1 | AMR | (383) |
| Tn*6330* | *Escherichia coli* | Composite transposon | 4.7 | NZ_CP029493.1 | AMR | (384) |
| Tn*6331* | *Streptococcus gallolyticus* | Conjugative transposon | - | - | AMR | (385) |
| Tn*6332* | *Bacillus sp.* | Unit transposon | 11.5 | LC155216.1 | Heavy metal resistance | - |
| Tn*6333* | *Escherichia coli* | Unit transposon | 11.5 | KX156773.1 | Heavy metal resistance | (386) |
| Tn*6338* | *Raoultella ornithinolytica* | Unit transposon | 33.2 | KY270853.1 | AMR, Antiseptic resistance, Efflux function,  Heavy metal resistance | (387) |
| Tn*6339* | *Klebsiella pneumoniae* | Unit transposon | 8.5 | MF344565.1 | AMR | (387) |
| Tn*6340* | *Klebsiella pneumoniae* | Unit transposon | 13.3 | MF344565.1 | AMR, Heavy metal resistance | - |
| Tn*6344* | *Klebsiella pneumoniae* | Composite transposon | 7.4 | MF344567.1 | - | (387) |
| Tn*6345* | *Pseudomonas aeruginosa* | Unit transposon | 6 | KU961660.2 | AMR | - |
| Tn*6346* | *Achromobacter sp.* | Unit transposon | 8.2 | EU696790.1 | Heavy metal resistance | (388) |
| Tn*6347* | *Klebsiella pneumoniae* | Unit transposon | 3 | MF344562.1 | Efflux function | (387) |
| Tn*6350* | *Pseudomonas aeruginosa* | Unit transposon | 7.4 | KY347015.1 | Virulence determinants | (389) |
| Tn*6352* | *Pseudomonas aeruginosa* | Unit transposon | 11 | KX889311.1 | AMR | (390) |
| Tn*6356* | *Pseudomonas aeruginosa* | Unit transposon | 15.6 | KY494864.1 | AMR, Efflux function | (391) |
| Tn*6360* | *Escherichia coli* | Composite transposon | 17 | KF534788.2 | AMR | (392) |
| Tn*6361* | *Morganella morganii* | Composite transposon | 16.3 | KM660724.1 | AMR | (392) |
| Tn*6362* | *Cronobacter sakazakii* | Unit transposon | 10.7 | KY978628.1 | Efflux function, Heavy metal resistance | (393) |
| Tn*6363* | *Cronobacter sakazakii* | Composite transposon | 6.1 | KY978630.1 | AMR | (393) |
| Tn*6367* | *Klebsiella pneumoniae* | Composite transposon | 5.7 | MF168404.1 | AMR | (394) |
| Tn*6368* | *Providencia rettgeri* | Unit transposon | 2.8 | KX832928.1 | - | (395) |
| Tn*6369* | *Providencia rettgeri* | Unit transposon | 9.2 | CP017672.1 | Efflux functions | (395) |
| Tn*6374* | *Shewanella oneidensis* | Unit transposon | - | - | - | (396) |
| Tn*6375* | *Klebsiella pneumoniae* | Unit transposon | 18.8 | KY978631.1 | AMR | (397) |
| Tn*6376* | *Citrobacter freundii* | Unit transposon | 4.9 | MF072963.1 | - | (398) |
| Tn*6377* | *Klebsiella pneumoniae* | Unit transposon | 4 | MF133495.1 | AMR | - |
| Tn*6378* | *Enterobacter cloacae* | Unit transposon | - | CP021851.1 | AMR | (397) |
| Tn*6381* | *Klebsiella pneumoniae* | Unit transposon | 22.8 | MF344566.1 | Heavy metal resistance | (387) |
| Tn*6382* | *Klebsiella pneumoniae* | Unit transposon | 20.4 | MF344566.1 | AMR, Antiseptic resistance, Heavy metal resistance | (387) |
| Tn*6383* | *Klebsiella pneumoniae* | Composite transposon and Unit transposon | 48.7 | MF344562.1 | AMR, Antiseptic resistance, Heavy metal resistance | (387) |
| Tn*6384* | *Klebsiella pneumoniae* | Composite transposon and Unit transposon | 26 | MF344563.1 | AMR, Antiseptic resistance, Heavy metal resistance | (387) |
| Tn*6390* | *Shigella flexneri* | Composite transposon | 4.5 | KY784668.1 | AMR | (399) |
| Tn*6391* | *Pseudomonas aeruginosa* | Unit transposon | 17.4 | MF168946.1 | AMR, Heavy metal resistance | - |
| Tn*6392* | *Pseudomonas aeruginosa* | Unit transposon | 14.5 | MF144194.1 | AMR, Heavy metal resistance | (400) |
| Tn*6393* | *Pseudomonas aeruginosa* | Composite transposon and Unit transposon | 47.1 | MF144194.1 | AMR | (400) |
| Tn*6394* | *Pseudomonas aeruginosa* | Unit transposon | 7.2 | MF344578.1 | AMR | (397) |
| Tn*6397* | *Enterobacter cloacae* | Conjugative transposon | 114 | CP021851.1 | AMR, Metabolism, Heavy metal resistance | (397) |
| Tn*6399* | *Klebsiella pneumoniae* | Unit transposon | 8.3 | MF344566.1 | - | - |
| Tn*6400* | *Klebsiella pneumoniae* | Unit transposon | 23.8 | KU318421.1 | AMR, Heavy metal resistance | (387) |
| Tn*6401* | *Raoultella planticola* | Composite transposon and Unit transposon | 39.3 | JX515588.1 | AMR | (387) |
| Tn*6402* | *Klebsiella pneumoniae* | Composite transposon | 9.4 | MF344563.1 | AMR | (387) |
| Tn*6403* | *Pseudomonas aeruginosa* | Composite transposon and Unit transposon | 30 | CP030075.1 | AMR, Heavy metal resistance | (400) |
| Tn*6404* | *Klebsiella pneumoniae* | Composite transposon | - | KX839207.1 | AMR, Antiseptic resistance, Heavy metal resistance | (401) |
| Tn*6411* | *Pseudomonas aeruginosa* | Composite transposon and Unit transposon | 37.6 | CP024477.1 | AMR | (397) |
| Tn*6413* | *Pseudomonas aeruginosa* | Conjugative transposon | 114.1 | CP030075.1 | AMR, Heavy metal resistance | (400) |
| Tn*6414* | *Escherichia coli* | Composite transposon and Unit transposon | 22.6 | KC340960.1 | AMR, Heavy metal resistance | (402) |
| Tn*6417* | *Pseudomonas aeruginosa* | Conjugative transposon | 108.2 | CP013993.1 | AMR, Heavy metal resistance | (400) |
| Tn*6431* | *Aeromonas caviae* | Composite transposon | 12.6 | CP025777.1 | AMR | (403) |
| Tn*6432* | *Aeromonas caviae* | Unit transposon | 9.5 | CP025777.1 | AMR | (404) |
| Tn*6435* | *Enterobacter hormaechei* | Composite transposon | 3.7 | KJ488943.1 | AMR | (404) |
| Tn*6450* | *Proteus mirabilis* | Composite transposon and Unit transposon | 65.8 | MF805806.1 | AMR | (405) |
| Tn*6451* | *Morganella morganii* | Composite transposon and Unit transposon | 111.2 | MG832661.1 | AMR | (406) |
| Tn*6452* | *Salmonella enterica* | Unit transposon | - | MK360094 | AMR | (407) |
| Tn*6456* | *Prevotella bivia* | Unit transposon | 7.8 | MG827401.1 | AMR, Efflux function | (408) |
| Tn*6497* | *Klebsiella pneumoniae* | Unit transposon | - | CP026021.1 | Virulence determinants | (409) |
| Tn*6498* | *Klebsiella pneumoniae* | Unit transposon | - | CP026021.1 | Virulence determinants | (409) |
| Tn*6499* | *Klebsiella pneumoniae* | Unit transposon | - | CP026021.1 | Efflux function | (409) |
| Tn*6501* | *Klebsiella pneumoniae* | Composite transposon | 6.9 | CP026022.1 | AMR | (409) |
| Tn*6505* | *Leclercia adecarboxylata* | Unit transposon | 16.9 | MK036884.1 | AMR, Heavy metal resistance | - |
| Tn*6516* | *Arthrobacter sp.* | Unit transposon | 14.9 | MH067970.1 | Efflux function, Heavy metal resistance | (410) |
| Tn*6517* | *Arthrobacter sp.* | Unit transposon | 7 | MH067971.1 | - | (410) |
| Tn*6518* | *Aeromonas veronii* | Not specified | - | MH481281 | AMR | - |
| Tn*6530* | *Pseudomonas aeruginosa* | Unit transposon | 35.1 | KX196168.1 | AMR, Heavy metal resistance | (400) |
| Tn*6531* | *Pseudomonas aeruginosa* | Unit transposon | 24.8 | AP014651.1 | AMR, Heavy metal resistance | (400) |
| Tn*6532* | *Pseudomonas aeruginosa* | Unit transposon | 21.2 | CP013993.1 | AMR, Heavy metal resistance | (400) |
| Tn*6533* | *Pseudomonas aeruginosa* | Conjugative transposon | 109 | AP014651.1 | AMR, Heavy metal resistance | (400) |
| Tn*6534* | *Pseudomonas aeruginosa* | Conjugative transposon | 118.7 | KX196168.1 | AMR, Heavy metal resistance | (400) |
| Tn*6535* | *Hafnia alvei* | Unit transposon | 14.3 | CP009706.1 | Heavy metal resistance | (387) |
| Tn*6539* | *Escherichia coli* | Composite transposon | 7.3 | KJ772290.2 | AMR | (411) |
| Tn*6540* | *Enterobacter cloacae* | Unit transposon | 16.1 | LT991958.1 | AMR | (412) |
| Tn*6628* | *Enterococcus faecium* | Composite transposon | 4.9 | MF580438.1 | AMR | (413) |
| Tn*6644* | *Streptococcus suis* | Composite transposon | 5.1 | KC844836.1 | AMR | (414) |
| Tn*6645* | *Acinetobacter baumannii* | Composite transposon | 8.4 | CP014234.1 | - | (415) |
| Tn*6647* | *Enterococcus faecalis* | Unit transposon | 32.8 | SAMN10475125 | - | (416) |
| Tn*6648* | *Enterococcus faecalis* | Unit transposon | 53.2 | SAMN10475125 | AMR | (416) |
| Tn*6649* | *Pseudomonas aeruginosa* | Unit transposon | 31.6 | CP033439.1 | AMR, Antiseptic resistance | - |
| Tn*6651* | *Escherichia coli* | Composite transposon | 3.9 | MH674341.1 | AMR | (417) |
| Tn*6652* | *Escherichia coli* | Composite transposon | 10.2 | MH674341.1 | AMR | (417) |
| Tn*6655* | *Acinetobacter baumannii* | Composite transposon | 3.2 | CP035186.1 | AMR | (418) |
| Tn*6656* | *Acinetobacter baumannii* | Composite transposon | 3.4 | CP035186.1 | AMR | (418) |
| Tn*6658* | *Acinetobacter baumannii* | Composite transposon | 3.9 | CP035186.1 | - | (418) |
| Tn*6674* | *Enterococcus faecalis* | Unit transposon | 12.9 | MK737778.1 | AMR | (419) |
| Tn*6677* | *Vibrio cholerae* | Unit transposon | - | - | Adaptive immune system | (420) |

**References**

1. Hedges RW, Jacob AE. Transposition of ampicillin resistance from RP4 to other replicons. Mol Gen Genet. 1974;132(1):31-40.

2. Partridge SR. What's in a Name? IS*Swi1* Corresponds to Transposons Related to Tn*2* and Tn*3*. mBio. 2015;6(5):e01344-15.

3. Chou J, Casadaban MJ, Lemaux PG, Cohen SN. Identification and characterization of a self-regulated repressor of translocation of the Tn*3* element. Proceedings of the National Academy of Sciences of the United States of America. 1979;76(8):4020-4.

4. Hyde DR, Tu CP. Insertion sites and the terminal nucleotide sequences of the Tn*4* transposon. Nucleic acids research. 1982;10(13):3981-93.

5. Auerswald EA, Ludwig G, Schaller H. Structural analysis of Tn*5*. Cold Spring Harbor symposia on quantitative biology. 1981;45 Pt 1:107-13.

6. Berg DE, Davies J, Allet B, Rochaix JD. Transposition of R factor genes to bacteriophage lambda. Proceedings of the National Academy of Sciences of the United States of America. 1975;72(9):3628-32.

7. Barth PT, Datta N, Hedges RW, Grinter NJ. Transposition of a deoxyribonucleic acid sequence encoding trimethoprim and streptomycin resistances from R483 to other replicons. Journal of bacteriology. 1976;125(3):800-10.

8. Alton NK, Vapnek D. Nucleotide sequence analysis of the chloramphenicol resistance transposon Tn*9*. Nature. 1979;282(5741):864-9.

9. Chalmers R, Sewitz S, Lipkow K, Crellin P. Complete Nucleotide Sequence of Tn*10*. Journal of bacteriology. 2000;182(10):2970-2.

10. Liebert CA, Hall RM, Summers AO. Transposon Tn*21*, flagship of the floating genome. Microbiology and molecular biology reviews : MMBR. 1999;63(3):507-22.

11. Shen T, Jia P, Na S, Men D. Determination of nucleotide sequence of *Corynebacterium* plasmid pXZ10145. Chinese journal of biotechnology. 1993;9(3):171-8.

12. Cohen SN, Shapiro JA. Transposable genetic elements. Scientific American. 1980;242(2):40-9.

13. Barth PT, Datta N. Two Naturally Occurring Transposons Indistinguishable from Tn*7*. Microbiology (Reading, England). 1977;102(1):129-34.

14. Barth PT, Ellis K, Bechhofer DH, Figurski DH. Involvement of *kil* and *kor* genes in the phenotype of a host-range mutant of RP4. Molecular and General Genetics MGG. 1984;197(2):236-43.

15. Poirel L, Bonnin RA, Boulanger A, Schrenzel J, Kaase M, Nordmann P. Tn*125*-related acquisition of *bla*_NDM_-like genes in *Acinetobacter baumannii*. Antimicrobial agents and chemotherapy. 2012;56(2):1087-9.

16. Ulrich A, Puhler A. The new class II transposon Tn*163* is plasmid-borne in two unrelated *Rhizobium leguminosarum* biovar *viciae* strains. Mol Gen Genet. 1994;242(5):505-16.

17. Bennett PM, Grinsted J, Choi CL, Richmond MH. Characterisation of Tn*501*, a transposon determining resistance to mercuric ions. Mol Gen Genet. 1978;159(1):101-6.

18. Stanisich VA, Arwas R, Bennett PM, De La Cruz F. Characterization of *Pseudomonas* mercury-resistance transposon Tn*502*, which has a preferred insertion site in RP1. Microbiology (Reading, England). 1989;135(11):2909-15.

19. Petrovski S, Stanisich VA. Embedded elements in the IncPβ plasmids R772 and R906 can be mobilized and can serve as a source of diverse and novel elements. Microbiology (Reading, England). 2011;157(6):1714-25.

20. Petrovski S, Blackmore DW, Jackson KL, Stanisich VA. Mercury(II)-resistance transposons Tn*502* and Tn*512*, from *Pseudomonas* clinical strains, are structurally different members of the Tn5053 family. Plasmid. 2011;65(1):58-64.

21. Tonin PN, Grant RB. Genetic and physical characterization of trimethoprim resistance plasmids from *Shigella sonnei* and *Shigella flexneri*. Canadian Journal of Microbiology. 1987;33(10):905-13.

22. Khan SA, Novick RP. Terminal nucleotide sequences of Tn*551*, a transposon specifying erythromycin resistance in *Staphylococcus aureus*: Homology with Tn*3*. Plasmid. 1980;4(2):148-54.

23. Rowland S-J, Dyke KGH. Tn*552*, a novel transposable element from *Staphylococcus aureus*. Molecular microbiology. 1990;4(6):961-75.

24. Phillips S, Novick RP. Tn*554*—a site-specific represser-controlled transposon in *Staphylococcus aureus*. Nature. 1979;278(5703):476-8.

25. Kehrenberg C, Schwarz S. Florfenicol-chloramphenicol exporter gene *fexA* is part of the novel transposon Tn*558*. Antimicrobial agents and chemotherapy. 2005;49(2):813-5.

26. Kadlec K, Schwarz S. Identification of the novel *dfrK*-carrying transposon Tn*559* in a porcine methicillin-susceptible *Staphylococcus aureus* ST398 strain. Antimicrobial agents and chemotherapy. 2010;54(8):3475-7.

27. Romanova Iu M, Ganelin VL, Denisov AA. Transposition of the kanamycin resistance determinant (Tn*601*) from plasmid 5T to the genome of bacteriophage lambda and the expression of this gene after prophage induction. Genetika. 1980;16(2):199-204.

28. Stibitz S, Davies JE. Tn*602*: A naturally occurring relative of Tn*903* with direct repeats. Plasmid. 1987;17(3):202-9.

29. Martin C, Timm J, Rauzier J, Gomez-Lus R, Davies J, Gicquel B. Transposition of an antibiotic resistance element in mycobacteria. Nature. 1990;345(6277):739-43.

30. Perez E, Gavigan JA, Otal I, Guilhot C, Pelicic V, Giquel B, et al. Tn*611* transposon mutagenesis in *Mycobacterium smegmatis* using a temperature-sensitive delivery system. Methods in molecular biology (Clifton, NJ). 1998;101:187-98.

31. Hopkins JD, Flores A, del Pilar Pla M, Lester S, O'Brien TF. Nosocomial spread of an amikacin resistance gene on both a mobilized, nonconjugative plasmid and a conjugative plasmid. Antimicrobial agents and chemotherapy. 1991;35(8):1605-11.

32. Han CG, Shiga Y, Tobe T, Sasakawa C, Ohtsubo E. Structural and functional characterization of IS*679* and IS*66*-family elements. Journal of bacteriology. 2001;183(14):4296-304.

33. Mindlin SZ, Minakhina SV, Kholodii G, Kopteva AV, Nikiforov VG. Incorporation of Tn*5053* and Tn*402* into various plasmids. Genetika. 1996;32(10):1426-30.

34. Nugent ME, Bone DH, Datta N. A transposon, Tn*732*, encoding gentamicin/tobramycin resistance. Nature. 1979;282:422.

35. Nugent ME, Datta N. Transposable Gentamicin Resistance in IncW Plasmids from Hammersmith Hospital. Microbiology (Reading, England). 1980;121(1):259-62.

36. Smith CA, Thomas CM. Narrow-host-range IncP Plasmid pHH502-1 Lacks a Complete IncP Replication System. Microbiology (Reading, England). 1987;133(8):2247-52.

37. Chouchani C, Ben-Achour N, M'Charek A, Belhadj O. Cloning and sequencing of the class A β-lactamase gene (*bla*_TEM-15_) located on a chromosomal Tn*801* transposon. Diagnostic Microbiology and Infectious Disease. 2007;58(4):459-63.

38. Grinsted J, Bennett PM, Higginson S, Richmond MH. Regional preference of insertion of Tn*501* and Tn*802* into RP1 and its derivatives. Mol Gen Genet. 1978;166(3):313-20.

39. Jorgensen ST, Oliva B, Grinsted J, Bennett PM. New translocation sequence mediating tetracycline resistance found in *Escherichia coli* pathogenic for piglets. Antimicrobial agents and chemotherapy. 1980;18(1):200-5.

40. Diver WP, Grinsted J, Fritzinger DC, Brown NL, Altenbuchner J, Rogowsky P, et al. DNA sequences of and complementation by the *tnpR* genes of Tn*21*, Tn*501* and Tn*1721*. Mol Gen Genet. 1983;191(2):189-93.

41. Heritage J, Hawkey PM, Todd N, Lewis IJ. Transposition of the gene encoding a TEM-12 extended-spectrum β-lactamase. Antimicrobial agents and chemotherapy. 1992;36(9):1981-6.

42. van Embden JDA. Translocation of an ampicillin resistance determinant within an R-factor aggregate in *Salmonella panama*. Antonie van Leeuwenhoek. 1978;44(2):203-18.

43. Iida S, Hiestand-Nauer R, Hänni C, Arber W. Reversion of a truncated gene for ampicillin resistance by genetic rearrangements in *Escherichia coli* K12. Molecular and General Genetics MGG. 1985;201(2):174-7.

44. Oka A, Sugisaki H, Takanami M. Nucleotide sequence of the kanamycin resistance transposon Tn*903*. Journal of molecular biology. 1981;147(2):217-26.

45. McCombie WR, Hansen JB, Zylstra GJ, Maurer B, Olsen RH. *Pseudomonas* streptomycin resistance transposon associated with R-plasmid mobilization. Journal of bacteriology. 1983;155(1):40-8.

46. Browne HP, Anvar SY, Frank J, Lawley TD, Roberts AP, Smits WK. Complete genome sequence of BS49 and draft genome sequence of BS34A, *Bacillus subtilis* strains carrying Tn*916*. FEMS Microbiology Letters. 2015;362(3):1-4.

47. Perkins JB, Youngman PJ. A physical and functional analysis of Tn*917*, a *Streptococcus* transposon in the Tn*3* family that functions in *Bacillus*. Plasmid. 1984;12(2):119-38.

48. Clewell DB, An FY, White BA, Gawron-Burke C. *Streptococcus faecalis* sex pheromone (cAM373) also produced by *Staphylococcus aureus* and identification of a conjugative transposon (Tn*918*). Journal of bacteriology. 1985;162(3):1212-20.

49. Fitzgerald GF, Clewell DB. A conjugative transposon (Tn*919*) in *Streptococcus sanguis*. Infection and immunity. 1985;47(2):415-20.

50. Thal LA, Chow JW, Clewell DB, Zervos MJ. Tn*924*, a chromosome-borne transposon encoding high-level gentamicin resistance in *Enterococcus faecalis*. Antimicrobial agents and chemotherapy. 1994;38(5):1152-6.

51. Christie PJ, Dunny GM. Identification of regions of the *Streptococcus faecalis* plasmid pCF-10 that encode antibiotic resistance and pheromone response functions. Plasmid. 1986;15(3):230-41.

52. Takeuchi K, Tomita H, Fujimoto S, Kudo M, Kuwano H, Ike Y. Drug resistance of *Enterococcus faecium* clinical isolates and the conjugative transfer of gentamicin and erythromycin resistance traits. FEMS Microbiology Letters. 2005;243(2):347-54.

53. Cornelis G, Ghosal D, Saedler H. Tn*951*: a new transposon carrying a lactose operon. Mol Gen Genet. 1978;160(2):215-24.

54. Reif HJ. Genetic evidence for absence of transposition functions from the internal part of Tn*981* a relative of Tn*9*. Mol Gen Genet. 1980;177(4):667-74.

55. Centola MB, Tsai MM, Deonier RC. Transposition of Tn*1000*: activity of single or directly repeated termini. Journal of bacteriology. 1987;169(12):5852-5.

56. Haines AS, Jones K, Batt SM, Kosheleva IA, Thomas CM. Sequence of plasmid pBS228 and reconstruction of the IncP-1α phylogeny. Plasmid. 2007;58(1):76-83.

57. Machida Y, Machida C, Ohtsubo E. A novel type of transposon generated by insertion element IS*102* present in a pSC101 derivative. Cell. 1982;30(1):29-36.

58. Brenciani A, Bacciaglia A, Vecchi M, Vitali LA, Varaldo PE, Giovanetti E. Genetic elements carrying *erm*(B) in *Streptococcus pyogenes* and association with *tet*(M) tetracycline resistance gene. Antimicrobial agents and chemotherapy. 2007;51(4):1209-16.

59. Poirel L, Cabanne L, Vahaboglu H, Nordmann P. Genetic environment and expression of the extended-spectrum β-lactamase *bla*_PER-1_ gene in gram-negative bacteria. Antimicrobial agents and chemotherapy. 2005;49(5):1708-13.

60. Santagati M, Iannelli F, Oggioni MR, Stefani S, Pozzi G. Characterization of a genetic element carrying the macrolide efflux gene *mef*(A) in *Streptococcus pneumoniae*. Antimicrobial agents and chemotherapy. 2000;44(9):2585-7.

61. Alavi MR, Antonic V, Ravizee A, Weina PJ, Izadjoo M, Stojadinovic A. An *Enterobacter* plasmid as a new genetic background for the transposon Tn*1331*. Infection and drug resistance. 2011;4:209-13.

62. Poirel L, Cabanne L, Collet L, Nordmann P. Class II transposon-borne structure harboring metallo-β-lactamase gene *bla*_VIM-2_ in *Pseudomonas putida*. Antimicrobial agents and chemotherapy. 2006;50(8):2889-91.

63. Medeiros AA, Hedges RW, Jacoby GA. Spread of a "*Pseudomonas*-specific" β-lactamase to plasmids of enterobacteria. Journal of bacteriology. 1982;149(2):700-7.

64. Stokes HW, Elbourne LD, Hall RM. Tn*1403*, a multiple-antibiotic resistance transposon made up of three distinct transposons. Antimicrobial agents and chemotherapy. 2007;51(5):1827-9.

65. Schnabel EL, Jones AL. Distribution of tetracycline resistance genes and transposons among phylloplane bacteria in Michigan apple orchards. Applied and environmental microbiology. 1999;65(11):4898-907.

66. Partridge SR, Brown HJ, Hall RM. Characterization and movement of the class 1 integron known as Tn*2521* and Tn*1405*. Antimicrobial agents and chemotherapy. 2002;46(5):1288-94.

67. Briaux-Gerbaud S, Gerbaud G, Jaffe-Brachet A. Transposition of a tetracycline-resistance determinant (Tn*1523*) and cointegration events mediated by the pIP231 plasmid in *Escherichia coli*. Gene. 1981;15(2-3):139-49.

68. Labigne-Roussel A, Briaux-Gerbaud S, Courvalin P. Tn*1525*, a kanamycin R determinant flanked by two direct copies of IS*15*. Molecular and General Genetics MGG. 1983;189(1):90-101.

69. Goldstein F, Gerbaud G, Courvalin P. Transposable resistance to trimethoprim and 0/129 in *Vibrio cholerae*. Journal of Antimicrobial Chemotherapy. 1986;17(5):559-69.

70. Lambert T, Gerbaud G, Courvalin P. Characterization of transposon Tn*1528*, which confers amikacin resistance by synthesis of aminoglycoside 3'-*O*-phosphotransferase type VI. Antimicrobial agents and chemotherapy. 1994;38(4):702-6.

71. Courvalin P, Carlier C. Tn*1545*: A conjugative shuttle transposon. Molecular and General Genetics MGG. 1987;206(2):259-64.

72. Arthur M, Molinas C, Depardieu F, Courvalin P. Characterization of Tn*1546*, a Tn*3*-related transposon conferring glycopeptide resistance by synthesis of depsipeptide peptidoglycan precursors in *Enterococcus faecium* BM4147. Journal of bacteriology. 1993;175(1):117-27.

73. Quintiliani R, Courvalin P. Characterization of Tn*1547*, a composite transposon flanked by the IS*16* and IS*256*-like elements, that confers vancomycin resistance in *Enterococcus faecalis* BM4281. Gene. 1996;172(1):1-8.

74. Galimand M, Sabtcheva S, Courvalin P, Lambert T. Worldwide disseminated *armA* aminoglycoside resistance methylase gene is borne by composite transposon Tn*1548*. Antimicrobial agents and chemotherapy. 2005;49(7):2949-53.

75. Garnier F, Taourit S, Glaser P, Courvalin P, Galimand M. Characterization of transposon Tn*1549*, conferring VanB-type resistance in *Enterococcus* spp. Microbiology (Reading, England). 2000;146(6):1481-9.

76. So M, McCarthy BJ. Nucleotide sequence of the bacterial transposon Tn*1681* encoding a heat-stable (ST) toxin and its identification in enterotoxigenic *Escherichia coli* strains. Proceedings of the National Academy of Sciences of the United States of America. 1980;77(7):4011-5.

77. Partridge SR, Brown HJ, Stokes HW, Hall RM. Transposons Tn*1696* and Tn*21* and their integrons In4 and In2 have independent origins. Antimicrobial agents and chemotherapy. 2001;45(4):1263-70.

78. Rubens CE, McNeill WF, Farrar WE, Jr. Evolution of multiple-antibiotic-resistance plasmids mediated by transposable plasmid deoxyribonucleic acid sequences. Journal of bacteriology. 1979;140(2):713-9.

79. Yamada Y, Calame KL, Grindley JN, Nakada D. Location of an ampicillin resistance transposon, Tn*1701*, in a group of small, nontransferring plasmids. Journal of bacteriology. 1979;137(2):990-9.

80. Rhodes G, Huys G, Swings J, McGann P, Hiney M, Smith P, et al. Distribution of oxytetracycline resistance plasmids between aeromonads in hospital and aquaculture environments: implication of Tn*1721* in dissemination of the tetracycline resistance determinant Tet A. Applied and environmental microbiology. 2000;66(9):3883-90.

81. Cross MA, Warne SR, Thomas CM. Analysis of the vegetative replication origin of broad-host-range plasmid RK2 by transposon mutagenesis. Plasmid. 1986;15(2):132-46.

82. Cram HK, Cram D, Skurray R. F plasmid pif region: Tn1725 mutagenesis and polypeptide analysis. Gene. 1984;32(1):251-4.

83. Jörg Kunte H, Galinski EA. Transposon mutagenesis in halophilic eubacteria: conjugal transfer and insertion of transposon Tn*5* and Tn*1732* in *Halomonas elongata*. FEMS Microbiology Letters. 1995;128(3):293-9.

84. Dobritsa AP, Dobritsa SV, Popov EI, Fedoseeva VB. Transposition of a DNA fragment flanked by two inverted Tn*1* sequences. Gene. 1981;14(3):217-25.

85. Dobritsa AP, Ivanova ZA, Fedoseeva VB. Transposition of DNA fragments flanked by two inverted Tn*1* sequences: translocation of the plasmid RP4::Tn*1* region harboring the Tc^r^ marker. Gene. 1983;22(2-3):237-43.

86. Schöffl F, Pühler A. Intramolecular amplification of the tetracycline resistance determinant of transposon Tn*1771* in *Escherichia coli*. Genetical Research. 1979;33(3):253-60.

87. Herron PR, Evans MC, Dyson PJ. Low target site specificity of an IS*6100*-based mini-transposon, Tn*1792*, developed for transposon mutagenesis of antibiotic-producing *Streptomyces*. FEMS Microbiology Letters. 1999;171(2):215-21.

88. Camilli R, Del Grosso M, Iannelli F, Pantosti A. New genetic element carrying the erythromycin resistance determinant *erm*(TR) in *Streptococcus pneumoniae*. Antimicrobial agents and chemotherapy. 2008;52(2):619-25.

89. Tietze E, Prager R, Tschäpe H. Characterization of the transposons Tn*1822* (Tc) and Tn*1824* (TpSm) and the light they throw on the natural spread of resistance genes. Plasmid. 1982;8(3):253-60.

90. Tschäpe H, Tietze E, Prager R, Voigt W, Wolter E, Seltmann G. Plasmid-borne streptothricin resistance in gram-negative bacteria. Plasmid. 1984;12(3):189-96.

91. Tietze E, Brevet J, Tschäpe H. Relationships among the streptothricin resistance transposons Tn*1825* and Tn*1826* and the trimethoprim resistance transposon Tn*7*. Plasmid. 1987;18(3):246-9.

92. Hooykaas PJJ, den Dulk-Ras H, Schilperoort RA. Molecular mechanism of Ti plasmid mobilization by R plasmids: Isolation of Ti plasmids with transposon-insertions in *Agrobacterium tumefaciens*. Plasmid. 1980;4(1):64-75.

93. Colonna B, Bernardini M, Micheli G, Maimone F, Nicoletti M, Casalino M. The *Salmonella wien* virulence plasmid pZM3 carries Tn*1935*, a multiresistance transposon containing a composite IS*1936*-kanamycin resistance element. Plasmid. 1988;20(3):221-31.

94. Aubert D, Naas T, Heritier C, Poirel L, Nordmann P. Functional characterization of IS*1999*, an IS*4* family element involved in mobilization and expression of β-lactam resistance genes. Journal of bacteriology. 2006;188(18):6506-14.

95. Naas T, Mikami Y, Imai T, Poirel L, Nordmann P. Characterization of In53, a class 1 plasmid- and composite transposon-located integron of *Escherichia coli* which carries an unusual array of gene cassettes. Journal of bacteriology. 2001;183(1):235-49.

96. Iyobe S, Sagai H, Mitsuhashi S. Tn*2001*, a transposon encoding chloramphenicol resistance in *Pseudomonas aeruginosa*. Journal of bacteriology. 1981;146(1):141-8.

97. Preston KE, Hitchcock SA, Aziz AY, Tine JA. The complete nucleotide sequence of the multi-drug resistance-encoding IncL/M plasmid pACM1. Plasmid. 2014;76:54-65.

98. Corvec S, Poirel L, Naas T, Drugeon H, Nordmann P. Genetics and expression of the carbapenem-hydrolyzing oxacillinase gene *bla*_OXA-23_ in *Acinetobacter baumannii*. Antimicrobial agents and chemotherapy. 2007;51(4):1530-3.

99. Nigro S, Hall RM. Distribution of the *bla*_OXA-23_-containing transposons Tn*2006* and Tn*2008* in Australian carbapenem-resistant *Acinetobacter baumannii* isolates. Journal of Antimicrobial Chemotherapy. 2015;70(8):2409-11.

100. Liu LL, Ji SJ, Ruan Z, Fu Y, Fu YQ, Wang YF, et al. Dissemination of *bla*_OXA-23_ in *Acinetobacter* spp. in China: main roles of conjugative plasmid pAZJ221 and transposon Tn*2009*. Antimicrobial agents and chemotherapy. 2015;59(4):1998-2005.

101. Li Y, Tomita H, Lv Y, Liu J, Xue F, Zheng B, et al. Molecular characterization of *erm*(B)- and *mef*(E)-mediated erythromycin-resistant *Streptococcus pneumoniae* in China and complete DNA sequence of Tn*2010*. Journal of applied microbiology. 2011;110(1):254-65.

102. Nakazawa H, Mitsuhashi S. Tn*2011*, a new transposon encoding oxacillin-hydrolyzing β-lactamase. Antimicrobial agents and chemotherapy. 1983;23(3):407-12.

103. Cattoir V, Nordmann P, Silva-Sanchez J, Espinal P, Poirel L. IS*Ecp1*-mediated transposition of *qnrB*-like gene in *Escherichia coli*. Antimicrobial agents and chemotherapy. 2008;52(8):2929-32.

104. Potron A, Nordmann P, Poirel L. Characterization of OXA-204, a carbapenem-hydrolyzing class D β-lactamase from *Klebsiella pneumoniae*. Antimicrobial agents and chemotherapy. 2013;57(1):633-6.

105. Del Grosso M, Camilli R, Libisch B, Fuzi M, Pantosti A. New composite genetic element of the Tn*916* family with dual macrolide resistance genes in a *Streptococcus pneumoniae* isolate belonging to clonal complex 271. Antimicrobial agents and chemotherapy. 2009;53(3):1293-4.

106. Halling SM, Zuerner RL. Evidence for lateral transfer to *Brucellae*: characterization of a locus with a Tn-like element (Tn*2020*). Biochimica et biophysica acta. 2002;1574(1):109-16.

107. Katsu K, Inoue M, Mitsuhashi S. Transposition of the carbenicillin-hydrolyzing beta-lactamase gene. Journal of bacteriology. 1982;150(2):483-9.

108. Johnson DA, Willetts NS. Tn*2301*, a transposon construct carrying the entire transfer region of the F plasmid. Journal of bacteriology. 1980;143(3):1171-8.

109. Clerget M, Chandler M, Caro L. Isolation of an IS*1* flanked kanamycin resistance transposon from R1*drd*19. Molecular and General Genetics MGG. 1980;180(1):123-7.

110. Dobritsa AP. IS*8*- and Tn*2353*-mediated cointegration of the plasmids R15 and RP4:: Tn*1*. Molecular and General Genetics MGG. 1984;194(1):206-10.

111. Dobritsa AP, Mikhailova TG, Dubovaya VI. Physical and genetic structure of the IncN plasmid R15. Plasmid. 1985;14(2):99-105.

112. Schmidt F, Kratz J, Wiedemann B. Identification of Tn*2401*, a Transposon Encoding Multiresistance to Aminoglycosides. Microbiology (Reading, England). 1983;129(5):1527-36.

113. Kratz J, Schmidt F, Wiedemann B. Characterization of Tn*2411* and Tn*2410*, two transposons derived from R-plasmid R1767 and related to Tn*2603* and Tn*21*. Journal of bacteriology. 1983;155(3):1333-42.

114. Meyer JF, Nies BA, Wiedemann B. Amikacin resistance mediated by multiresistance transposon Tn*2424*. Journal of bacteriology. 1983;155(2):755-60.

115. Meyer JF, Nies BA, Kratz J, Widedmann B. Evolution of Tn*21*-related Transposons: Isolation of Tn*2425*, which Harbours IS*161*. Microbiology (Reading, England). 1985;131(5):1123-30.

116. Zühlsdorf MT, Wiedemann B. Functional and physiological characterization of the Tn*21* cassette for resistance genes in Tn*2426*. Microbiology (Reading, England). 1993;139(5):995-1002.

117. Nies BA, Meyer JF, Wiedemann B. Tn*2440*, a composite tetracycline resistance transposon with direct repeated copies of IS*160* at its flanks. Microbiology (Reading, England). 1985;131(9):2443-7.

118. Michiels T, Cornelis G, Ellis K, Grinsted J. Tn*2501*, a component of the lactose transposon Tn*951*, is an example of a new category of class II transposable elements. Journal of bacteriology. 1987;169(2):624-31.

119. Neyt C, Iriarte M, Thi VH, Cornelis GR. Virulence and arsenic resistance in *Yersiniae*. Journal of bacteriology. 1997;179(3):612-9.

120. Eppinger M, Radnedge L, Andersen G, Vietri N, Severson G, Mou S, et al. Novel plasmids and resistance phenotypes in *Yersinia pestis*: unique plasmid inventory of strain java 9 mediates high levels of arsenic resistance. PloS one. 2012;7(3):e32911.

121. Michiels T, Cornelis G. Tn*951* derivatives designed for high-frequency plasmid-specific transposition and deletion mutagenesis. Gene. 1986;43(3):175-81.

122. Mulder B, Michiels T, Simonet M, Sory MP, Cornelis G. Identification of additional virulence determinants on the pYV plasmid of *Yersinia enterocolitica* W227. Infect Immun. 1989;57(8):2534-41.

123. Krishnapillai V, Pühler A, Lanka E. Molecular cloning into Tn*5* and integration in the *Pseudomonas aeruginosa* chromosome: a tool for heterologous gene expression. Microbiology (Reading, England). 1986;132(3):707-15.

124. Yamamoto T, Katoh R, Shimazu A, Yamagishi S. Gene expression of ampicillin resistance transposons, Tn*2601* and Tn*2602*. Microbiology and immunology. 1980;24(6):479-94.

125. Yamamoto T, Tanaka M, Baba R, Yamagishi S. Physical and functional mapping of Tn*2603*, a transposon encoding ampicillin, streptomycin, sulfonamide, and mercury resistance. Mol Gen Genet. 1981;181(4):464-9.

126. Tanaka M, Yamamoto T, Sawai T. Evolution of complex resistance transposons from an ancestral mercury transposon. Journal of bacteriology. 1983;153(3):1432-8.

127. Takaya A, Watanabe M, Yamamoto T. Organization of Tn*2610* containing two transposition modules. Antimicrobial agents and chemotherapy. 2006;50(4):1143-7.

128. Iida S, Kulka I, Meyer J, Arber W. Amplification of drug resistance genes flanked by inversely repeated IS*1* elements: involvement of IS*1*-promoted DNA rearrangements before amplification. Journal of bacteriology. 1987;169(4):1447-53.

129. Iida S, Meyer J, Arber W. Cointegrates between bacteriophage P1 DNA and plasmid pBR322 derivatives suggest molecular mechanisms for P1-mediated transduction of small plasmids. Mol Gen Genet. 1981;184(1):1-10.

130. Chiang SJ, Clowes RC. Intramolecular transposition and inversion in plasmid R6K. Journal of bacteriology. 1980;142(2):668-82.

131. Partridge SR, Hall RM. Complex multiple antibiotic and mercury resistance region derived from the r-det of NR1 (R100). Antimicrobial agents and chemotherapy. 2004;48(11):4250-5.

132. Iida S, Hänni C, Echarti C, Arber W. Is the IS*1*-flanked r-Determinant of the R Plasmid NR1 a Transposon? Microbiology (Reading, England). 1981;126(2):413-25.

133. Hanni C, Meyer J, Iida S, Arber W. Occurrence and properties of composite transposon Tn*2672*: evolution of multiple drug resistance transposons. Journal of bacteriology. 1982;150(3):1266-73.

134. Mollet B, Clerget M, Meyer J, Iida S. Organization of the Tn*6*-related kanamycin resistance transposon Tn*2680* carrying two copies of IS*26* and an IS*903* variant, IS*903* .B. Journal of bacteriology. 1985;163(1):55-60.

135. Casadesus J, Naas T, Garzon A, Arini A, Torreblanca J, Arber W. Lack of hotspot targets: a constraint for IS*30* transposition in *Salmonella*. Gene. 1999;238(1):231-9.

136. York MK, Stodolsky M. Characterization of P1*arg*F derivatives from *Escherichia coli* K12 transduction. Molecular and General Genetics MGG. 1981;181(2):230-40.

137. Seoane A, Sangari FJ, Lobo JM. Complete nucleotide sequence of the fosfomycin resistance transposon Tn*2921*. International journal of antimicrobial agents. 2010;35(4):413-4.

138. Martin C, Gomez-Lus R, Ortiz JM, Garcia-Lobo JM. Structure and mobilization of an ampicillin and gentamicin resistance determinant. Antimicrobial agents and chemotherapy. 1987;31(8):1266-70.

139. Campos JC, da Silva MJ, dos Santos PR, Barros EM, Pereira Mde O, Seco BM, et al. Characterization of Tn*3000*, a transposon responsible for *bla*_NDM-1_ dissemination among *Enterobacteriaceae* in Brazil, Nepal, Morocco, and India. Antimicrobial agents and chemotherapy. 2015;59(12):7387-95.

140. Terakado N, Sekizaki T, Hashimoto K, Yamagata S, Yamamoto T. Chloramphenicol transposons found in *Salmonella naestved* and *Escherichia coli* of domestic animal origin. Antimicrobial agents and chemotherapy. 1981;20(3):382-6.

141. Radford AJ, Oliver J, Kelly WJ, Reanney DC. Translocatable resistance to mercuric and phenylmercuric ions in soil bacteria. Journal of bacteriology. 1981;147(3):1110-2.

142. Ishiguro N, Sato G. Nucleotide sequence of insertion sequence IS*3411*, which flanks the citrate utilization determinant of transposon Tn*3411*. Journal of bacteriology. 1988;170(4):1902-6.

143. Bartosik D, Sochacka M, Baj J. Identification and characterization of transposable elements of *Paracoccus pantotrophus*. Journal of bacteriology. 2003;185(13):3753-63.

144. Erova TE, Anisimova LA, Kochetkov VV, Boronin AM. Characteristics of Tn*3611* and Tn*3613* transposons discovered in the chromosome of the BS205 strain of *Pseudomonas aeruginosa*. Genetika. 1988;24(5):781-90.

145. Mäe AA, Heinaru AL. Transposon-mediated mobilization of chromosomally located catabolic operons of the CAM plasmid by TOL plasmid transposon Tn*4652* and CAM plasmid transposon Tn*3614*. Microbiology (Reading, England). 1994;140(4):915-21.

146. Bräu B, Piepersberg W. Cointegrational transduction and mobilization of gentamicin resistance plasmid pWP14a is mediated by IS*140*. Molecular and General Genetics MGG. 1983;189(2):298-303.

147. Le Bouguénec C, de Cespédès G, Horaud T. Molecular analysis of a composite chromosomal conjugative element (Tn*3701*) of *Streptococcus pyogenes*. Journal of bacteriology. 1988;170(9):3930-6.

148. Horaud T, Delbos F, de Cespédès G. Tn*3702*, a conjugative transposon in *Enterococcus faecalis*. FEMS Microbiology Letters. 1990;72(1):189-94.

149. Clermont D, Horaud T. Genetic and molecular studies of a composite chromosomal element (Tn*3705*) containing a Tn*916*-modified structure (Tn*3704*) in *Streptococcus anginosus* F22. Plasmid. 1994;31(1):40-8.

150. Horaud T, de Cespedes G, Trieu-Cuot P. Chromosomal gentamicin resistance transposon Tn*3706* in *Streptococcus agalactiae* B128. Antimicrobial agents and chemotherapy. 1996;40(5):1085-90.

151. Townsend DE, Ashdown N, Greed LC, Grubb WB. Transposition of gentamicin resistance to staphylococcal plasmids encoding resistance to cationic agents. Journal of Antimicrobial Chemotherapy. 1984;14(2):115-24.

152. Townsend D, Bolton S, Ashdown N, Annear D, Grubb W. Conjugative, staphylococcal plasmids carrying hitch-hiking transposons similar to Tn*554*: intra-and interspecies dissemination of erythromycin resistance. Australian Journal of Experimental Biology and Medical Science. 1986;64(4):367-79.

153. Udo EE, Grubb WB. Transposition of genes encoding kanamycin, neomycin and streptomycin resistance in *Staphylococcus aureus*. Journal of Antimicrobial Chemotherapy. 1991;27(6):713-20.

154. Banai M, LeBlanc DJ. *Streptococcus faecalis* R plasmid pJH1 contains an erythromycin resistance transposon (Tn*3871*) similar to transposon Tn*917*. Journal of bacteriology. 1984;158(3):1172-4.

155. Poyart C, Quesne G, Acar P, Berche P, Trieu-Cuot P. Characterization of the Tn*916*-like transposon Tn*3872* in a strain of *Abiotrophia defectiva* (*Streptococcus defectivus*) causing sequential episodes of endocarditis in a child. Antimicrobial agents and chemotherapy. 2000;44(3):790-3.

156. Osbourn SEV, Turner AK, Grinsted J. Nucleotide sequence within Tn*3926* confirms this as a Tn*21*-like transposable element and provides evidence for the origin of the *mer* operon carried by plasmid pKLH2. Plasmid. 1995;33(1):65-9.

157. Vijayakumar MN, Priebe SD, Guild WR. Structure of a conjugative element in *Streptococcus pneumoniae*. Journal of bacteriology. 1986;166(3):978-84.

158. Schmidt FRJ, Nücken EJ, Henschke RB. Nucleotide sequence analysis of 2″-aminoglycoside nucleotidyl-transferase ANT(2″) from Tn*4000*: its relationship with AAD(3″) and impact on Tn*21* evolution. Molecular microbiology. 1988;2(6):709-17.

159. Mahairas GG, Lyon BR, Skurray RA, Pattee PA. Genetic analysis of *Staphylococcus aureus* with Tn*4001*. Journal of bacteriology. 1989;171(7):3968-72.

160. Gillespie MT, Skurray RA. Nucleotide sequence of the blaZ gene of the Staphylococcus aureus beta-lactamase transposon Tn4002. Nucleic acids research. 1989;17(21):8854.

161. Rouch DA, Messerotti LJ, Loo LSL, Jackson CA, Skurray RA. Trimethoprim resistance transposon Tn*4003* from *Staphylococcus aureus* encodes genes for a dihydrofolate reductase and thymidylate synthetase flanked by three copies of IS*257*. Molecular microbiology. 1989;3(2):161-75.

162. Thomas WD, Jr., Archer GL. Mobility of gentamicin resistance genes from staphylococci isolated in the United States: identification of Tn*4031*, a gentamicin resistance transposon from *Staphylococcus epidermidis*. Antimicrobial agents and chemotherapy. 1989;33(8):1335-41.

163. Young H-K, Qumsieh MJ, Mclntosh ML. Nucleotide sequence and genetic analysis of the type Ib trimethoprim-resistant, Tn*4132*-encoded dihydrofolate reductase. Journal of Antimicrobial Chemotherapy. 1994;34(5):715-25.

164. Mantengoli E, Rossolini GM. Tn*5393*d, a complex Tn*5393* derivative carrying the PER-1 extended-spectrum beta-lactamase gene and other resistance determinants. Antimicrobial agents and chemotherapy. 2005;49(8):3289-96.

165. Weber DA, Goering RV. Tn*4201*, a β-lactamase transposon in *Staphylococcus aureus*. Antimicrobial agents and chemotherapy. 1988;32(8):1164-9.

166. Trees DL, Iandolo JJ. Identification of a *Staphylococcus aureus* transposon (Tn*4291*) that carries the methicillin resistance gene(s). Journal of bacteriology. 1988;170(1):149-54.

167. Thorsted PB, Macartney DP, Akhtar P, Haines AS, Ali N, Davidson P, et al. Complete sequence of the IncPβ plasmid R751: implications for evolution and organisation of the IncP backbone. Journal of molecular biology. 1998;282(5):969-90.

168. Hoover CI, Abarbarchuk E, Ng CY, Felton JR. Transposition of Tn*4351* in *Porphyromonas gingivalis*. Plasmid. 1992;27(3):246-50.

169. Wrighton CJ, Strike P. A pathway for the evolution of the plasmid NTP16 involving the novel kanamycin resistance transposon Tn*4352*. Plasmid. 1987;17(1):37-45.

170. Merlin C, Springael D, Toussaint A. Tn*4371*: A modular structure encoding a phage-like integrase, a *Pseudomonas*-like catabolic pathway, and RP4/Ti-like transfer functions. Plasmid. 1999;41(1):40-54.

171. Champier L, Duarte V, Michaud-Soret I, Covès J. Characterization of the MerD protein from *Ralstonia metallidurans* CH34: a possible role in bacterial mercury resistance by switching off the induction of the *mer* operon. Molecular microbiology. 2004;52(5):1475-85.

172. Murphy CG, Malamy MH. Characterization of a "mobilization cassette" in transposon Tn*4399* from *Bacteroides fragilis*. Journal of bacteriology. 1993;175(18):5814-23.

173. Robillard NJ, Tally FP, Malamy MH. Tn*4400*, a compound transposon isolated from *Bacteroides fragilis*, functions in *Escherichia coli*. Journal of bacteriology. 1985;164(3):1248-55.

174. Cuzon G, Naas T, Nordmann P. Functional characterization of Tn*4401*, a Tn*3*-based transposon involved in *bla*_KPC_ gene mobilization. Antimicrobial agents and chemotherapy. 2011;55(11):5370-3.

175. Divers M, Craven PL, Vivian A. Molecular analysis of an antibiotic resistance plasmid, pAV5, and its derivative plasmids in *Acinetobacter calcoaceticus*. Microbiology (Reading, England). 1985;131(12):3367-74.

176. Lereclus D, Mahillon J, Menou G, Lecadet M-M. Identification of Tn*4430*, a transposon of *Bacillus thuringiensis* functional in *Escherichia coli*. Molecular and General Genetics MGG. 1986;204(1):52-7.

177. Steinmann D, Wiggerich H-G, Klauke B, Schramm U, Pühler A, Priefer UB. Saturation mutagenesis in *Escherichia coli* of a cloned *Xanthomonas campestris* DNA fragment with the *lux* transposon Tn*4431* using the delivery plasmid pDS1, thermosensitive in replication. Applied microbiology and biotechnology. 1993;40(2):356-60.

178. Bannam TL, Crellin PK, Rood JI. Molecular genetics of the chloramphenicol-resistance transposon Tn*4451* from *Clostridium perfringens*: the TnpX site-specific recombinase excises a circular transposon molecule. Molecular microbiology. 1995;16(3):535-51.

179. Abraham LJ, Rood JI. Identification of Tn*4451* and Tn*4452*, chloramphenicol resistance transposons from *Clostridium perfringens*. Journal of bacteriology. 1987;169(4):1579-84.

180. Hu ST, Lee CH. Characterization of the transposon carrying the STII gene of enterotoxigenic *Escherichia coli*. Molecular and General Genetics MGG. 1988;214(3):490-5.

181. Dumans AT, Carvalho MF, Coelho A. Tn*4527*, a Tp Sp/Sm transposon related to Tn*7* and flanked by IS*1*. Plasmid. 1989;22(3):256-9.

182. Smith CJ, Owen C, Kirby L. Activation of a cryptic streptomycin-resistance gene in the *Bacteroides* *erm* transposon, Tn*4551*. Molecular microbiology. 1992;6(16):2287-97.

183. Bacic MK, Smith CJ. Analysis of chromosomal insertion sites for *Bacteroides* Tn*4555* and the role of TnpA. Gene. 2005;353(1):80-8.

184. Chung ST. Tn*4556*, a 6.8-kilobase-pair transposable element of *Streptomyces fradiae*. Journal of bacteriology. 1987;169(10):4436-41.

185. Bhatt A, Stewart GR, Kieser T. Transposition of Tn*4560* of *Streptomyces fradiae* in *Mycobacterium smegmatis*. FEMS Microbiology Letters. 2002;206(2):241-6.

186. Schauer AT, Nelson AD, Daniel JB. Tn*4563* transposition in *Streptomyces coelicolor* and its application to isolation of new morphological mutants. Journal of bacteriology. 1991;173(16):5060-7.

187. Genka H, Nagata Y, Tsuda M. Site-specific recombination system encoded by toluene catabolic transposon Tn*4651*. Journal of bacteriology. 2002;184(17):4757-66.

188. Tsuda M, Minegishi K, Iino T. Toluene transposons Tn*4651* and Tn*4653* are class II transposons. Journal of bacteriology. 1989;171(3):1386-93.

189. Sota M, Yano H, Ono A, Miyazaki R, Ishii H, Genka H, et al. Genomic and functional analysis of the IncP-9 naphthalene-catabolic plasmid NAH7 and its transposon Tn*4655* suggests catabolic gene spread by a tyrosine recombinase. Journal of bacteriology. 2006;188(11):4057-67.

190. Tsuda M, Genka H. Identification and characterization of Tn*4656*, a novel class II transposon carrying a set of toluene-degrading genes from TOL plasmid pWW53. Journal of bacteriology. 2001;183(21):6215-24.

191. Yano H, Garruto CE, Sota M, Ohtsubo Y, Nagata Y, Zylstra GJ, et al. Complete sequence determination combined with analysis of transposition/site-specific recombination events to explain genetic organization of IncP-7 TOL plasmid pWW53 and related mobile genetic elements. Journal of molecular biology. 2007;369(1):11-26.

192. Yano H, Genka H, Ohtsubo Y, Nagata Y, Top EM, Tsuda M. Cointegrate-resolution of toluene-catabolic transposon Tn*4651*: determination of crossover site and the segment required for full resolution activity. Plasmid. 2013;69(1):24-35.

193. Sota M, Kawasaki H, Tsuda M. Structure of haloacetate-catabolic IncP-1beta plasmid pUO1 and genetic mobility of its residing haloacetate-catabolic transposon. Journal of bacteriology. 2003;185(22):6741-5.

194. Maeda K, Nojiri H, Shintani M, Yoshida T, Habe H, Omori T. Complete nucleotide sequence of carbazole/dioxin-degrading plasmid pCAR1 in *Pseudomonas resinovorans* strain CA10 indicates its mosaicity and the presence of large catabolic transposon Tn*4676*. Journal of molecular biology. 2003;326(1):21-33.

195. Tenzen T, Matsutani S, Ohtsubo E. Site-specific transposition of insertion sequence IS*630*. Journal of bacteriology. 1990;172(7):3830-6.

196. Chen CW, Yu TW, Chung HM, Chou CF. Discovery and characterization of a new transposable element, Tn*4811*, in *Streptomyces lividans* 66. Journal of bacteriology. 1992;174(23):7762-9.

197. Halula M, Macrina FL. Tn*5030*: A conjugative transposon conferring clindamycin resistance in *Bacteroides* species. Clinical Infectious Diseases. 1990;12(Supplement_2):S235-S42.

198. Fletcher HM, Marri L, Daneo-Moore L. Transposon-*916*-Iike elements in clinical isolates of *Enterococcus faecium*. Microbiology (Reading, England). 1989;135(11):3067-77.

199. Yurieva O, Kholodii G, Minakhin L, Gorlenko Z, Kalyaeva E, Mindlin S, et al. Intercontinental spread of promiscuous mercury-resistance transposons in environmental bacteria. Molecular microbiology. 1997;24(2):321-9.

200. Kaliaeva ES, Kholodii G, Bass IA, Gorlenko Zh M, Iur'eva OV, Nikiforov VG. Tn*5037*-a Tn*21*-like mercury transposon, detected in *Thiobacillus ferrooxidans*. Genetika. 2001;37(8):1160-4.

201. Kholodii GY, Yurieva OV, Gorlenko ZM, Mindlin SZ, Bass IA, Lomovskay OL, et al. Tn*5041*: a chimeric mercury resistance transposon closely related to the toluene degradative transposon Tn*4651*. Microbiology (Reading, England). 1997;143(8):2549-56.

202. Mindlin S, Minakhin L, Petrova M, Kholodii G, Minakhina S, Gorlenko Z, et al. Present-day mercury resistance transposons are common in bacteria preserved in permafrost grounds since the Upper Pleistocene. Res Microbiol. 2005;156(10):994-1004.

203. Kholodii G, Yurieva O, Mindlin S, Gorlenko Z, Rybochkin V, Nikiforov V. Tn*5044*, a novel Tn*3* family transposon coding for temperature-sensitive mercury resistance. Res Microbiol. 2000;151(4):291-302.

204. Petrova M, Gorlenko Z, Mindlin S. Tn*5045*, a novel integron-containing antibiotic and chromate resistance transposon isolated from a permafrost bacterium. Res Microbiol. 2011;162(3):337-45.

205. Mindlin S, Kholodii G, Gorlenko Z, Minakhina S, Minakhin L, Kalyaeva E, et al. Mercury resistance transposons of gram-negative environmental bacteria and their classification. Res Microbiol. 2001;152(9):811-22.

206. Kholodii GY, Yurieva OV, Lomovskaya OL, Gorlenko ZM, Mindlin SZ, Nikiforov VG. Tn*5053*, a Mercury Resistance Transposon with Integron's Ends. Journal of molecular biology. 1993;230(4):1103-7.

207. Norberg P, Bergström M, Hermansson M. Complete nucleotide sequence and analysis of two conjugative broad host range plasmids from a marine microbial biofilm. PloS one. 2014;9(3):e92321.

208. Kholodii G, Mindlin S, Petrova M, Minakhina S. Tn*5060* from the Siberian permafrost is most closely related to the ancestor of Tn*21* prior to integron acquisition. FEMS Microbiology Letters. 2003;226(2):251-5.

209. Essa AM, Julian DJ, Kidd SP, Brown NL, Hobman JL. Mercury resistance determinants related to Tn*21*, Tn*1696*, and Tn*5053* in enterobacteria from the preantibiotic era. Antimicrobial agents and chemotherapy. 2003;47(3):1115-9.

210. Bogdanova E, Minakhin L, Bass I, Volodin A, Hobman JL, Nikiforov V. Class II broad-spectrum mercury resistance transposons in Gram-positive bacteria from natural environments. Res Microbiol. 2001;152(5):503-14.

211. Sundstrom L, Swedberg G, Skold O. Characterization of transposon Tn*5086*, carrying the site-specifically inserted gene *dhfrVII* mediating trimethoprim resistance. Journal of bacteriology. 1993;175(6):1796-805.

212. Radstrom P, Skold O, Swedberg G, Flensburg J, Roy PH, Sundstrom L. Transposon Tn*5090* of plasmid R751, which carries an integron, is related to Tn*7*, Mu, and the retroelements. Journal of bacteriology. 1994;176(11):3257-68.

213. Baltz RH, Hahn DR, McHenney MA, Solenberg PJ. Transposition of Tn*5096* and related transposons in *Streptomyces* species. Gene. 1992;115(1):61-5.

214. Hahn DR, Solenberg PJ, McHenney MA, Baltz RH. Transposition and transduction of plasmid DNA in *Streptomyces* spp. Journal of industrial microbiology. 1991;7(4):229-34.

215. Solenberg PJ, Cantwell CA, Tietz AJ, Mc Gilvray D, Queener SW, Baltz RH. Transposition mutagenesis in *Streptomyces fradiae*: identification of a neutral site for the stable insertion of DNA by transposon exchange. Gene. 1996;168(1):67-72.

216. Hahn DR, Solenberg PJ, Baltz RH. Tn*5099*, a *xylE* promoter probe transposon for *Streptomyces* spp. Journal of bacteriology. 1991;173(17):5573-7.

217. Santoro F, Oggioni MR, Pozzi G, Iannelli F. Nucleotide sequence and functional analysis of the *tet*(M)-carrying conjugative transposon Tn*5251* of *Streptococcus pneumoniae*. FEMS Microbiology Letters. 2010;308(2):150-8.

218. Kilic AO, Vijayakumar MN, al-Khaldi SF. Identification and nucleotide sequence analysis of a transfer-related region in the streptococcal conjugative transposon Tn*5252*. Journal of bacteriology. 1994;176(16):5145-50.

219. Iannelli F, Santoro F, Oggioni MR, Pozzi G. Nucleotide sequence analysis of integrative conjugative element Tn*5253* of *Streptococcus pneumoniae*. Antimicrobial agents and chemotherapy. 2014;58(2):1235-9.

220. Nakatsu C, Ng J, Singh R, Straus N, Wyndham C. Chlorobenzoate catabolic transposon Tn*5271* is a composite class I element with flanking class II insertion sequences. Proceedings of the National Academy of Sciences of the United States of America. 1991;88(19):8312-6.

221. Rauch PJ, De Vos WM. Characterization of the novel nisin-sucrose conjugative transposon Tn*5276* and its insertion in *Lactococcus lactis*. Journal of bacteriology. 1992;174(4):1280-7.

222. Horn N, Swindell S, Dodd H, Gasson M. Nisin biosynthesis genes are encoded by a novel conjugative transposon. Molecular and General Genetics MGG. 1991;228(1):129-35.

223. Thompson J, Sackett DL, Donkersloot JA. Purification and properties of fructokinase I from *Lactococcus lactis*. Localization of *scrK* on the sucrose-nisin transposon Tn*5306*. The Journal of biological chemistry. 1991;266(33):22626-33.

224. Rice LB, Marshall SH, Carias LL. Tn*5381*, a conjugative transposon identifiable as a circular form in *Enterococcus faecalis*. Journal of bacteriology. 1992;174(22):7308-15.

225. Carias LL, Rudin SD, Donskey CJ, Rice LB. Genetic linkage and cotransfer of a novel, *vanB*-containing transposon (Tn*5382*) and a low-affinity penicillin-binding protein 5 gene in a clinical vancomycin-resistant *Enterococcus faecium* isolate. Journal of bacteriology. 1998;180(17):4426-34.

226. Rice LB, Carias LL, Marshall SH. Tn*5384*, a composite enterococcal mobile element conferring resistance to erythromycin and gentamicin whose ends are directly repeated copies of IS*256*. Antimicrobial agents and chemotherapy. 1995;39(5):1147-53.

227. Rice LB, Carias LL. Transfer of Tn*5385*, a composite, multiresistance chromosomal element from *Enterococcus faecalis*. Journal of bacteriology. 1998;180(3):714-21.

228. Rice LB, Carias LL, Marshall SH, Hutton-Thomas R, Rudin S. Characterization of Tn*5386*, a Tn*916*-related mobile element. Plasmid. 2007;58(1):61-7.

229. Chiou CS, Jones AL. Nucleotide sequence analysis of a transposon (Tn*5393*) carrying streptomycin resistance genes in *Erwinia amylovora* and other gram-negative bacteria. Journal of bacteriology. 1993;175(3):732-40.

230. McGhee GC, Schnabel EL, Maxson-Stein K, Jones B, Stromberg VK, Lacy GH, et al. Relatedness of chromosomal and plasmid DNAs of *Erwinia pyrifoliae* and *Erwinia amylovora*. Applied and environmental microbiology. 2002;68(12):6182-92.

231. Elhai J, Cai Y, Wolk CP. Conduction of pEC22, a plasmid coding for MR.*Eco*T22I, mediated by a resident Tn*3*-like transposon, Tn*5396*. Journal of bacteriology. 1994;176(16):5059-67.

232. Roberts AP, Johanesen PA, Lyras D, Mullany P, Rood JI. Comparison of Tn*5397* from *Clostridium difficile*, Tn*916* from *Enterococcus faecalis* and the CW459*tet*(M) element from *Clostridium perfringens* shows that they have similar conjugation regions but different insertion and excision modules. Microbiology (Reading, England). 2001;147(5):1243-51.

233. Baum JA. Tn*5401*, a new class II transposable element from *Bacillus thuringiensis*. Journal of bacteriology. 1994;176(10):2835-45.

234. Rinkel M, Hubert J-C, Roux B, Lett M-C. Identification of a new transposon Tn*5403* in a *Klebsiella pneumoniae* strain isolated from a polluted aquatic environment. Current microbiology. 1994;29(5):249-54.

235. Derbise A, Dyke KGH, El Solh N. Rearrangements in the staphylococcal β-lactamase-encoding plasmid, plP1066, including a DNA inversion that generates two alternative transposons. Molecular microbiology. 1995;17(4):769-79.

236. Derbise A, de Cespedes G, Solh NE. Nucleotide sequence of the *Staphylococcus aureus* transposon, Tn*5405*, carrying aminoglycosides resistance genes. Journal of Basic Microbiology. 1997;37(5):379-84.

237. Haroche J, Allignet J, El Solh N. Tn*5406*, a new staphylococcal transposon conferring resistance to streptogramin a and related compounds including dalfopristin. Antimicrobial agents and chemotherapy. 2002;46(8):2337-43.

238. Lebrun M, Audurier A, Cossart P. Plasmid-borne cadmium resistance genes in *Listeria monocytogenes* are present on Tn*5422*, a novel transposon closely related to Tn*917*. Journal of bacteriology. 1994;176(10):3049-61.

239. Žgur-Bertok D, Ambrožič J, Grabnar M. Tn*5431* arose by transposition of Tn*3* into Tn*1721*. Canadian Journal of Microbiology. 1996;42(12):1274-6.

240. Tauch A, Kassing F, Kalinowski J, Pühler A. The *Corynebacterium xerosis* composite transposon Tn*5432* consists of two identical insertion sequences, designated IS*1249*,flanking the erythromycin resistance gene *ermCX*. Plasmid. 1995;34(2):119-31.

241. Raze D, Dardenne O, Hallut S, Martinez-Bueno M, Coyette J, Ghuysen JM. The gene encoding the low-affinity penicillin-binding protein 3r in *Enterococcus hirae* S185R is borne on a plasmid carrying other antibiotic resistance determinants. Antimicrobial agents and chemotherapy. 1998;42(3):534-9.

242. Clennel AM, Johnston B, Rawlings DE. Structure and function of Tn*5467*, a Tn*21*-like transposon located on the *Thiobacillus ferrooxidans* broad-host-range plasmid pTF-FC2. Applied and environmental microbiology. 1995;61(12):4223-9.

243. Oppon JC, Sarnovsky RJ, Craig NL, Rawlings DE. A Tn*7*-like transposon is present in the *glmUS* region of the obligately chemoautolithotrophic bacterium *Thiobacillus ferrooxidans*. Journal of bacteriology. 1998;180(11):3007-12.

244. Kahn K, Schaefer MR. Characterization of transposon Tn*5469* from the cyanobacterium *Fremyella diplosiphon*. Journal of bacteriology. 1995;177(24):7026-32.

245. Immonen T, Wahlström G, Takala T, Saris PEJ. Evidence for a Mosaic Structure of the Tn*5482* in *Lactococcus lactis* N8. DNA Sequence. 1998;9(5-6):245-61.

246. Fischer J, Maier H, Viell P, Altenbuchner J. The use of an improved transposon mutagenesis system for DNA sequencing leads to the characterization of a new insertion sequence of *Streptomyces lividans* 66. Gene. 1996;180(1):81-9.

247. Volff JN, Altenbuchner J. High-frequency transposition of IS*1373*, the insertion sequence delimiting the amplifiable element *AUD2* of *Streptomyces lividans*. Journal of bacteriology. 1997;179(17):5639-42.

248. Volff J-N, Altenbuchner J. High frequency transposition of the Tn*5* derivative Tn*5493* in *Streptomyces lividans*. Gene. 1997;194(1):81-6.

249. Onaca C, Kieninger M, Engesser KH, Altenbuchner J. Degradation of alkyl methyl ketones by *Pseudomonas veronii* MEK700. Journal of bacteriology. 2007;189(10):3759-67.

250. Lauf U, Müller C, Herrmann H. The transposable elements resident on the plasmids of *Pseudomonas putida* strain H, Tn*5501* and Tn*5502*, are cryptic transposons of the Tn*3* family. Molecular and General Genetics MGG. 1998;259(6):674-8.

251. Heaton MP, Discotto LF, Pucci MJ, Handwerger S. Mobilization of vancomycin resistance by transposon-mediated fusion of a VanA plasmid with an *Enterococcus faecium* sex pheromone-response plasmid. Gene. 1996;171(1):9-17.

252. Vedantam G, Knopf S, Hecht DW. *Bacteroides fragilis* mobilizable transposon Tn*5520* requires a 71 base pair origin of transfer sequence and a single mobilization protein for relaxosome formation during conjugation. Molecular microbiology. 2006;59(1):288-300.

253. Poh RPC, Smith ARW, Bruce IJ. Complete characterisation of Tn*5530* from *Burkholderia cepacia* strain 2a (pIJB1) and studies of 2,4-dichlorophenoxyacetate uptake by the organism. Plasmid. 2002;48(1):1-12.

254. Bonamy C, Labarre J, Cazaubon L, Jacob C, Le Bohec F, Reyes O, et al. The mobile element IS*1207* of *Brevibacterium lactofermentum* ATCC21086: isolation and use in the construction of Tn*5531*, a versatile transposon for insertional mutagenesis of *Corynebacterium glutamicum*. Journal of biotechnology. 2003;104(1-3):301-9.

255. Schaefer MR, Kahn K. Cyanobacterial transposons Tn*5469* and Tn*5541* represent a novel noncomposite transposon family. Journal of bacteriology. 1998;180(22):6059-63.

256. Fong KPY, Goh CBH, Tan H-M. The genes for benzene catabolism in *Pseudomonas putida* ML2 are flanked by two copies of the insertion element IS*1489*, forming a class-I-type catabolic transposon, Tn*5542*. Plasmid. 2000;43(2):103-10.

257. Nagy I, Schoofs G, Vanderleyden J, De Mot R. Transposition of the IS*21*-related element IS*1415* in *Rhodococcus erythropolis*. Journal of bacteriology. 1997;179(14):4635-8.

258. Yeo CC, Tham JM, Kwong SM, Yiin S, Poh CL. Tn*5563*, a transposon encoding putative mercuric ion transport proteins located on plasmid pRA2 of *Pseudomonas alcaligenes*. FEMS Microbiology Letters. 1998;165(2):253-60.

259. Tauch A, Zheng Z, Pühler A, Kalinowski J. *Corynebacterium striatum* chloramphenicol resistance transposon Tn*5564*: genetic organization and transposition in *Corynebacterium glutamicum*. Plasmid. 1998;40(2):126-39.

260. Brynestad S, Granum PE. Evidence that Tn*5565*, which includes the enterotoxin gene in *Clostridium perfringens*, can have a circular form which may be a transposition intermediate. FEMS Microbiology Letters. 1999;170(1):281-6.

261. Segura A, Bunz PV, D'Argenio DA, Ornston LN. Genetic analysis of a chromosomal region containing *vanA* and *vanB*, genes required for conversion of either ferulate or vanillate to protocatechuate in *Acinetobacter*. Journal of bacteriology. 1999;181(11):3494-504.

262. Kehrenberg C, Werckenthin C, Schwarz S. Tn*5706*, a transposon-like element from *Pasteurella multocida* mediating tetracycline resistance. Antimicrobial agents and chemotherapy. 1998;42(8):2116-8.

263. Ogawa N, Miyashita K. The chlorocatechol-catabolic transposon Tn*5707* of *Alcaligenes eutrophus* NH9, carrying a gene cluster highly homologous to that in the 1,2,4-trichlorobenzene-degrading bacterium *Pseudomonas sp.* strain P51, confers the ability to grow on 3-chlorobenzoate. Applied and environmental microbiology. 1999;65(2):724-31.

264. Schneiker S, Keller M, Dröge M, Lanka E, Pühler A, Selbitschka W. The genetic organization and evolution of the broad host range mercury resistance plasmid pSB102 isolated from a microbial population residing in the rhizosphere of alfalfa. Nucleic acids research. 2001;29(24):5169-81.

265. Tauch A, Schlüter A, Bischoff N, Goesmann A, Meyer F, Pühler A. The 79,370-bp conjugative plasmid pB4 consists of an IncP-1β backbone loaded with a chromate resistance transposon, the *strA*-*strB* streptomycin resistance gene pair, the oxacillinase gene *bla*_NPS-1_, and a tripartite antibiotic efflux system of the resistance-nodulation-division family. Molecular Genetics and Genomics. 2003;268(5):570-84.

266. Dufour A, Rince A, Uguen P, Le Pennec JP. IS*1675*, a novel lactococcal insertion element, forms a transposon-like structure including the lacticin 481 lantibiotic operon. Journal of bacteriology. 2000;182(19):5600-5.

267. de Vries LE, Hasman H, Jurado Rabadán S, Agersø Y. Sequence-based characterization of Tn*5801*-like genomic islands in tetracycline-resistant *Staphylococcus pseudintermedius* and other gram-positive bacteria from humans and animals. Frontiers in Microbiology. 2016;7(576).

268. Roberts AP, Davis IJ, Seville L, Villedieu A, Mullany P. Characterization of the ends and target site of a novel tetracycline resistance-encoding conjugative transposon from *Enterococcus faecium* 664.1H1. Journal of bacteriology. 2006;188(12):4356-61.

269. Tseng SP, Hsueh PR, Tsai JC, Teng LJ. Tn*6001*, a transposon-like element containing the *bla*_VIM-3_-harboring integron In450. Antimicrobial agents and chemotherapy. 2007;51(11):4187-90.

270. Warburton PJ, Palmer RM, Munson MA, Wade WG. Demonstration of in vivo transfer of doxycycline resistance mediated by a novel transposon. Journal of Antimicrobial Chemotherapy. 2007;60(5):973-80.

271. Cochetti I, Tili E, Vecchi M, Manzin A, Mingoia M, Varaldo PE, et al. New Tn*916*-related elements causing *erm*(B)-mediated erythromycin resistance in tetracycline-susceptible pneumococci. Journal of Antimicrobial Chemotherapy. 2007;60(1):127-31.

272. Endimiani A, Luzzaro F, Migliavacca R, Mantengoli E, Hujer AM, Hujer KM, et al. Spread in an Italian hospital of a clonal *Acinetobacter baumannii* strain producing the TEM-92 extended-spectrum β-lactamase. Antimicrobial agents and chemotherapy. 2007;51(6):2211-4.

273. Labbate M, Roy Chowdhury P, Stokes HW. A class 1 integron present in a human commensal has a hybrid transposition module compared to Tn*402*: evidence of interaction with mobile DNA from natural environments. Journal of bacteriology. 2008;190(15):5318-27.

274. Soge OO, Beck NK, White TM, No DB, Roberts MC. A novel transposon, Tn*6009*, composed of a Tn*916* element linked with a *Staphylococcus aureus mer* operon. Journal of Antimicrobial Chemotherapy. 2008;62(4):674-80.

275. Norman A, Hansen LH, She Q, Sorensen SJ. Nucleotide sequence of pOLA52: a conjugative IncX1 plasmid from *Escherichia coli* which enables biofilm formation and multidrug efflux. Plasmid. 2008;60(1):59-74.

276. Han X, Ito T, Takeuchi F, Ma XX, Takasu M, Uehara Y, et al. Identification of a novel variant of staphylococcal cassette chromosome *mec*, type II.5, and Its truncated form by insertion of putative conjugative transposon Tn*6012*. Antimicrobial agents and chemotherapy. 2009;53(6):2616-9.

277. Smyth DS, Robinson DA. Integrative and sequence characteristics of a novel genetic element, ICE*6013*, in *Staphylococcus aureus*. Journal of bacteriology. 2009;191(19):5964-75.

278. de Vries LE, Christensen H, Skov RL, Aarestrup FM, Agersø Y. Diversity of the tetracycline resistance gene *tet*(M) and identification of Tn*916*- and Tn*5801*-like (Tn*6014*) transposons in *Staphylococcus aureus* from humans and animals. Journal of Antimicrobial Chemotherapy. 2009;64(3):490-500.

279. Xiong J, Alexander DC, Ma JH, Deraspe M, Low DE, Jamieson FB, et al. Complete sequence of pOZ176, a 500-kilobase IncP-2 plasmid encoding IMP-9-mediated carbapenem resistance, from outbreak isolate *Pseudomonas aeruginosa* 96. Antimicrobial agents and chemotherapy. 2013;57(8):3775-82.

280. Xiong J, Deraspe M, Iqbal N, Ma J, Jamieson FB, Wasserscheid J, et al. Genome and plasmid analysis of *bla*_IMP-4_-carrying *Citrobacter freundii* B38. Antimicrobial agents and chemotherapy. 2016;60(11):6719-25.

281. Post V, Hall RM. AbaR5, a large multiple-antibiotic resistance region found in *Acinetobacter baumannii*. Antimicrobial agents and chemotherapy. 2009;53(6):2667-71.

282. Post V, White PA, Hall RM. Evolution of AbaR-type genomic resistance islands in multiply antibiotic-resistant *Acinetobacter baumannii*. Journal of Antimicrobial Chemotherapy. 2010;65(6):1162-70.

283. Hamidian M, Hall RM. AbaR4 replaces AbaR3 in a carbapenem-resistant *Acinetobacter baumannii* isolate belonging to global clone 1 from an Australian hospital. Journal of Antimicrobial Chemotherapy. 2011;66(11):2484-91.

284. Cain AK, Hall RM. Transposon Tn*5393*e carrying the *aphA1*-containing transposon Tn*6023* Upstream of *strAB* does not confer resistance to streptomycin. Microbial Drug Resistance. 2011;17(3):389-94.

285. Cain AK, Liu X, Djordjevic SP, Hall RM. Transposons Related to Tn*1696* in IncHI2 Plasmids in Multiply Antibiotic Resistant *Salmonella enterica* Serovar Typhimurium from Australian Animals. Microbial Drug Resistance. 2010;16(3):197-202.

286. Cain AK, Hall RM. Evolution of a multiple antibiotic resistance region in IncHI1 plasmids: reshaping resistance regions *in situ*. Journal of Antimicrobial Chemotherapy. 2012;67(12):2848-53.

287. Arai H, Kanbe H, Ishii M, Igarashi Y. Complete genome sequence of the thermophilic, obligately chemolithoautotrophic hydrogen-oxidizing bacterium *Hydrogenobacter thermophilus* TK-6. Journal of bacteriology. 2010;192(10):2651-2.

288. Ghosh S, Sadowsky MJ, Roberts MC, Gralnick JA, LaPara TM. *Sphingobacterium sp.* strain PM2-P1-29 harbours a functional *tet*(X) gene encoding for the degradation of tetracycline. Journal of applied microbiology. 2009;106(4):1336-42.

289. Suenaga H, Koyama Y, Miyakoshi M, Miyazaki R, Yano H, Sota M, et al. Novel organization of aromatic degradation pathway genes in a microbial community as revealed by metagenomic analysis. The ISME journal. 2009;3:1335.

290. Ryan MP, Pembroke JT, Adley CC. Novel Tn*4371*-ICE like element in *Ralstonia pickettiiand* Genome mining for comparative elements. BMC Microbiology. 2009;9(1):242.

291. Tsubakishita S, Kuwahara-Arai K, Baba T, Hiramatsu K. Staphylococcal cassette chromosome *mec*-like element in *Macrococcus caseolyticus*. Antimicrobial agents and chemotherapy. 2010;54(4):1469-75.

292. Van Houdt R, Monchy S, Leys N, Mergeay M. New mobile genetic elements in *Cupriavidus metallidurans* CH34, their possible roles and occurrence in other bacteria. Antonie van Leeuwenhoek. 2009;96(2):205-26.

293. Mingoia M, Tili E, Manso E, Varaldo PE, Montanari MP. Heterogeneity of Tn*5253*-like composite elements in clinical *Streptococcus pneumoniae* isolates. Antimicrobial agents and chemotherapy. 2011;55(4):1453-9.

294. Roy Chowdhury P, Merlino J, Labbate M, Cheong EY, Gottlieb T, Stokes HW. Tn*6060*, a transposon from a genomic island in a *Pseudomonas aeruginosa* clinical isolate that includes two class 1 integrons. Antimicrobial agents and chemotherapy. 2009;53(12):5294-6.

295. Coyne S, Courvalin P, Galimand M. Acquisition of multidrug resistance transposon Tn*6061* and IS*6100*-mediated large chromosomal inversions in *Pseudomonas aeruginosa* clinical isolates. Microbiology (Reading, England). 2010;156(5):1448-58.

296. Holt KE, Phan MD, Baker S, Duy PT, Nga TVT, Nair S, et al. Emergence of a Globally Dominant IncHI1 Plasmid Type Associated with Multiple Drug Resistant Typhoid. PLOS Neglected Tropical Diseases. 2011;5(7):e1245.

297. Krol JE, Penrod JT, McCaslin H, Rogers LM, Yano H, Stancik AD, et al. Role of IncP-1β plasmids pWDL7::*rfp* and pNB8c in chloroaniline catabolism as determined by genomic and functional analyses. Applied and environmental microbiology. 2012;78(3):828-38.

298. Chen L, Mediavilla JR, Smyth DS, Chavda KD, Ionescu R, Roberts RB, et al. Identification of a novel transposon (Tn*6072*) and a truncated staphylococcal cassette chromosome *mec* element in methicillin-resistant *Staphylococcus aureus* ST239. Antimicrobial agents and chemotherapy. 2010;54(8):3347-54.

299. Brouwer MSM, Warburton PJ, Roberts AP, Mullany P, Allan E. Genetic organisation, mobility and predicted functions of genes on integrated, mobile genetic elements in sequenced strains of *Clostridium difficile*. PloS one. 2011;6(8):e23014.

300. Seth-Smith HM, Fookes MC, Okoro CK, Baker S, Harris SR, Scott P, et al. Structure, diversity, and mobility of the *Salmonella* pathogenicity island 7 family of integrative and conjugative elements within *Enterobacteriaceae*. Journal of bacteriology. 2012;194(6):1494-504.

301. de Vries LE, Vallès Y, Agersø Y, Vaishampayan PA, García-Montaner A, Kuehl JV, et al. The gut as reservoir of antibiotic resistance: microbial diversity of tetracycline resistance in mother and infant. PloS one. 2011;6(6):e21644.

302. Chen TL, Lee YT, Kuo SC, Hsueh PR, Chang FY, Siu LK, et al. Emergence and Distribution of Plasmids Bearing the *bla*_OXA-51_-like gene with an upstream IS*Aba1* in carbapenem-resistant *Acinetobacter baumannii* isolates in Taiwan. Antimicrobial agents and chemotherapy. 2010;54(11):4575-81.

303. Sampei G-i, Furuya N, Tachibana K, Saitou Y, Suzuki T, Mizobuchi K, et al. Complete genome sequence of the incompatibility group I1 plasmid R64. Plasmid. 2010;64(2):92-103.

304. Rice LB, Carias LL, Rudin S, Hutton RA, Marshall S. Multiple copies of functional, Tet(M)-encoding Tn*916*-like elements in a clinical *Enterococcus faecium* isolate. Plasmid. 2010;64(3):150-5.

305. Ciric L, Mullany P, Roberts AP. Antibiotic and antiseptic resistance genes are linked on a novel mobile genetic element: Tn*6087*. Journal of Antimicrobial Chemotherapy. 2011;66(10):2235-9.

306. Ye J, Su L-H, Chen C-L, Hu S, Wang J, Yu J, et al. Analysis of pSC138, the multidrug resistance plasmid of *Salmonella enterica* serotype Choleraesuis SC-B67. Plasmid. 2011;65(2):132-40.

307. D'Andrea MM, Nucleo E, Luzzaro F, Giani T, Migliavacca R, Vailati F, et al. CMY-16, a novel acquired AmpC-type β-lactamase of the CMY/LAT lineage in multifocal monophyletic isolates of *Proteus mirabilis* from northern Italy. Antimicrobial agents and chemotherapy. 2006;50(2):618-24.

308. Dziewit L, Baj J, Szuplewska M, Maj A, Tabin M, Czyzkowska A, et al. Insights into the Transposable Mobilome of *Paracoccus* spp. (*Alphaproteobacteria*). PloS one. 2012;7(2):e32277.

309. Machielsen R, Siezen RJ, van Hijum SA, van Hylckama Vlieg JE. Molecular description and industrial potential of Tn*6098* conjugative transfer conferring alpha-galactoside metabolism in *Lactococcus lactis*. Applied and environmental microbiology. 2011;77(2):555-63.

310. Tribble GD, Garza JJ, Yeung VL, Rigney TW, Dao D-HV, Rodrigues PH, et al. Genetic analysis of mobile *tetQ* elements in oral *Prevotella* species. Anaerobe. 2010;16(6):604-9.

311. Sajjad A, Holley MP, Labbate M, Stokes HW, Gillings MR. Preclinical class 1 integron with a complete Tn*402*-like transposition module. Applied and environmental microbiology. 2011;77(1):335-7.

312. D'Andrea MM, Literacka E, Zioga A, Giani T, Baraniak A, Fiett J, et al. Evolution and spread of a multidrug-resistant *Proteus mirabilis* clone with chromosomal AmpC-type cephalosporinases in Europe. Antimicrobial agents and chemotherapy. 2011;55(6):2735-42.

313. Boon N, Goris J, De Vos P, Verstraete W, Top EM. Genetic diversity among 3-chloroaniline- and aniline-degrading strains of the *Comamonadaceae*. Applied and environmental microbiology. 2001;67(3):1107-15.

314. Schwendener S, Perreten V. New transposon Tn*6133* in methicillin-resistant *Staphylococcus aureus* ST398 contains *vga*(E), a novel streptogramin A, pleuromutilin, and lincosamide resistance gene. Antimicrobial agents and chemotherapy. 2011;55(10):4900-4.

315. Nagata Y, Natsui S, Endo R, Ohtsubo Y, Ichikawa N, Ankai A, et al. Genomic organization and genomic structural rearrangements of *Sphingobium japonicum* UT26, an archetypal gamma-hexachlorocyclohexane-degrading bacterium. Enzyme Microb Technol. 2011;49(6-7):499-508.

316. Naito M, Hirakawa H, Yamashita A, Ohara N, Shoji M, Yukitake H, et al. Determination of the genome sequence of *Porphyromonas gingivalis* strain ATCC 33277 and genomic comparison with strain W83 revealed extensive genome rearrangements in *P. gingivalis*. DNA Research. 2008;15(4):215-25.

317. Martinez E, Marquez C, Ingold A, Merlino J, Djordjevic SP, Stokes HW, et al. Diverse mobilized class 1 integrons are common in the chromosomes of pathogenic *Pseudomonas aeruginosa* clinical isolates. Antimicrobial agents and chemotherapy. 2012;56(4):2169-72.

318. Corver J, Bakker D, Brouwer MSM, Harmanus C, Hensgens MP, Roberts AP, et al. Analysis of a *Clostridium difficile* PCR ribotype 078 100 kilobase island reveals the presence of a novel transposon, Tn*6164*. BMC Microbiology. 2012;12(1):130.

319. Nigro SJ, Hall RM. Antibiotic resistance islands in A320 (RUH134), the reference strain for *Acinetobacter baumannii* global clone 2. Journal of Antimicrobial Chemotherapy. 2011;67(2):335-8.

320. Nigro SJ, Hall RM. Tn*6167*, an antibiotic resistance island in an Australian carbapenem-resistant *Acinetobacter baumannii* GC2, ST92 isolate. Journal of Antimicrobial Chemotherapy. 2012;67(6):1342-6.

321. Hamidian M, Hall RM. Tn*6168*, a transposon carrying an IS*Aba1*-activated *ampC* gene and conferring cephalosporin resistance in *Acinetobacter baumannii*. Journal of Antimicrobial Chemotherapy. 2013;69(1):77-80.

322. J. HC, M. HR. pRMH760, a precursor of A/C2 plasmids carrying *bla*_CMY_ and *bla*_NDM_ genes. Microbial Drug Resistance. 2014;20(5):416-23.

323. Hamidian M, Hawkey J, Holt KE, Hall RM. Genome sequence of *Acinetobacter baumannii* strain D36, an antibiotic-resistant isolate from lineage 2 of global clone 1. Genome announcements. 2015;3(6).

324. Hamidian M, Ambrose SJ, Hall RM. A large conjugative *Acinetobacter baumannii* plasmid carrying the *sul2* sulphonamide and *strAB* streptomycin resistance genes. Plasmid. 2016;87-88:43-50.

325. Blackwell GA, Holt KE, Bentley SD, Hsu LY, Hall RM. Variants of AbGRI3 carrying the *armA* gene in extensively antibiotic-resistant *Acinetobacter baumannii* from Singapore. Journal of Antimicrobial Chemotherapy. 2017;72(4):1031-9.

326. Harmer CJ, Hamidian M, Hall RM. pIP40a, a type 1 IncC plasmid from 1969 carries the integrative element GI*sul2* and a novel class II mercury resistance transposon. Plasmid. 2017;92:17-25.

327. Goto T, Tanaka K, Minh Tran C, Watanabe K. Complete sequence of pBFUK1, a carbapenemase-harboring mobilizable plasmid from *Bacteroides fragilis*, and distribution of pBFUK1-like plasmids among carbapenem-resistant *B. fragilis* clinical isolates. The Journal of antibiotics. 2012;66:239.

328. Doublet B, Boyd D, Douard G, Praud K, Cloeckaert A, Mulvey MR. Complete nucleotide sequence of the multidrug resistance IncA/C plasmid pR55 from *Klebsiella pneumoniae* isolated in 1969. Journal of Antimicrobial Chemotherapy. 2012;67(10):2354-60.

329. Müller A, Rychli K, Muhterem-Uyar M, Zaiser A, Stessl B, Guinane CM, et al. Tn*6188* - A novel transposon in *Listeria monocytogenes* responsible for tolerance to benzalkonium chloride. PloS one. 2013;8(10):e76835.

330. Zong Z. Characterization of a complex context containing *mecA* but lacking genes encoding cassette chromosome recombinases in *Staphylococcus haemolyticus*. BMC Microbiology. 2013;13(1):64.

331. He M, Miyajima F, Roberts P, Ellison L, Pickard DJ, Martin MJ, et al. Emergence and global spread of epidemic healthcare-associated *Clostridium difficile*. Nature Genetics. 2012;45:109.

332. He M, Sebaihia M, Lawley TD, Stabler RA, Dawson LF, Martin MJ, et al. Evolutionary dynamics of *Clostridium difficile* over short and long time scales. Proceedings of the National Academy of Sciences of the United States of America. 2010;107(16):7527-32.

333. Wailan AM, Sidjabat HE, Yam WK, Alikhan NF, Petty NK, Sartor AL, et al. Mechanisms involved in acquisition of *bla*_NDM_ genes by IncA/C_2_ and IncFII_Y_ plasmids. Antimicrobial agents and chemotherapy. 2016;60(7):4082-8.

334. Bertsch D, Uruty A, Anderegg J, Lacroix C, Perreten V, Meile L. Tn*6198*, a novel transposon containing the trimethoprim resistance gene *dfrG* embedded into a Tn*916* element in *Listeria monocytogenes*. Journal of Antimicrobial Chemotherapy. 2013;68(5):986-91.

335. Boyd DA, Mulvey MR. The VanE operon in *Enterococcus faecalis* N00-410 is found on a putative integrative and conjugative element, Tn*6202*. Journal of Antimicrobial Chemotherapy. 2012;68(2):294-9.

336. Hu Y, Zhu Y, Ma Y, Liu F, Lu N, Yang X, et al. Genomic insights into intrinsic and acquired drug resistance mechanisms in *Achromobacter xylosoxidans*. Antimicrobial agents and chemotherapy. 2015;59(2):1152-61.

337. Zhu L, Yan Z, Zhang Z, Zhou Q, Zhou J, Wakeland EK, et al. Complete genome analysis of three *Acinetobacter baumannii* clinical isolates in China for insight into the diversification of drug resistance elements. PloS one. 2013;8(6):e66584.

338. Butler MI, Stockwell PA, Black MA, Day RC, Lamont IL, Poulter RTM. *Pseudomonas syringae* pv. *actinidiae* from recent outbreaks of kiwifruit bacterial canker belong to different clones that originated in China. PloS one. 2013;8(2):e57464.

339. David B, Geoffrey T, Jeff F, Elizabeth B, Joanne E, Denise G, et al. Complete sequence of four multidrug-resistant MOBQ1 plasmids harboring *bla*_GES-5_ isolated from *Escherichia coli* and *Serratia marcescens* persisting in a hospital in Canada. Microbial Drug Resistance. 2015;21(3):253-60.

340. Goh S, Hussain H, Chang BJ, Emmett W, Riley TV, Mullany P. Phage ϕC2 mediates transduction of Tn*6215*, encoding erythromycin resistance, between *Clostridium difficile* strains. mBio. 2013;4(6):e00840-13.

341. Wendel AF, Kaase M, Autenrieth IB, Peter S, Oberhettinger P, Rieber H, et al. Protracted regional dissemination of GIM-1-producing *Serratia marcescens* in Western Germany. Antimicrobial agents and chemotherapy. 2017;61(3).

342. Dingle KE, Elliott B, Robinson E, Griffiths D, Eyre DW, Stoesser N, et al. Evolutionary history of the *Clostridium difficile* pathogenicity locus. Genome Biology and Evolution. 2013;6(1):36-52.

343. Chowdhury PR, Charles IG, Djordjevic SP. A role for Tn6029 in the evolution of the complex antibiotic resistance gene loci in genomic island 3 in enteroaggregative hemorrhagic *Escherichia coli* O104:H4. PloS one. 2015;10(2):e0115781.

344. Raftis EJ, Forde BM, Claesson MJ, O’Toole PW. Unusual genome complexity in *Lactobacillus salivarius* JCM1046. BMC Genomics. 2014;15(1):771.

345. Nielsen TK, Xu Z, Gözdereliler E, Aamand J, Hansen LH, Sørensen SR. Novel insight into the genetic context of the *cadAB* genes from a 4-chloro-2-methylphenoxyacetic acid-degrading *Sphingomonas*. PloS one. 2014;8(12):e83346.

346. Mataseje LF, Boyd DA, Lefebvre B, Bryce E, Embree J, Gravel D, et al. Complete sequences of a novel *bla*_NDM-1_-harbouring plasmid from *Providencia rettgeri* and an FII-type plasmid from *Klebsiella pneumoniae* identified in Canada. Journal of Antimicrobial Chemotherapy. 2013;69(3):637-42.

347. Peters JE, Fricker AD, Kapili BJ, Petassi MT. Heteromeric transposase elements: generators of genomic islands across diverse bacteria. Molecular microbiology. 2014;93(6):1084-92.

348. Loftie-Eaton W, Yano H, Burleigh S, Simmons RS, Hughes JM, Rogers LM, et al. Evolutionary Paths That Expand Plasmid Host-Range: Implications for Spread of Antibiotic Resistance. Molecular Biology and Evolution. 2015;33(4):885-97.

349. Sakai Y, Ogawa N, Shimomura Y, Fujii T. A 2,4-dichlorophenoxyacetic acid degradation plasmid pM7012 discloses distribution of an unclassified megaplasmid group across bacterial species. Microbiology (Reading, England). 2014;160(3):525-36.

350. Di Pilato V, Arena F, Giani T, Conte V, Cresti S, Rossolini GM. Characterization of pFOX-7a, a conjugative IncL/M plasmid encoding the FOX-7 AmpC-type β-lactamase, involved in a large outbreak in a neonatal intensive care unit. Journal of Antimicrobial Chemotherapy. 2014;69(10):2620-4.

351. Knetsch CW, Connor TR, Mutreja A, van Dorp SM, Sanders IM, Browne HP, et al. Whole genome sequencing reveals potential spread of *Clostridium difficile* between humans and farm animals in the Netherlands, 2002 to 2011. Eurosurveillance. 2014;19(45):20954.

352. Beyrouthy R, Robin F, Delmas J, Gibold L, Dalmasso G, Dabboussi F, et al. IS*1R*-mediated plasticity of IncL/M plasmids leads to the insertion of *bla*_OXA-48_ into the *Escherichia coli* Chromosome. Antimicrobial agents and chemotherapy. 2014;58(7):3785-90.

353. Quiroga MP, Orman B, Errecalde L, Kaufman S, Centron D. Characterization of Tn*6238* with a new allele of *aac(6')-Ib-cr*. Antimicrobial agents and chemotherapy. 2015;59(5):2893-7.

354. Roy Chowdhury P, McKinnon J, Liu M, Djordjevic SP. Multidrug resistant uropathogenic *Escherichia coli* ST405 with a novel, composite IS*26* transposon in a unique chromosomal location. Frontiers in Microbiology. 2019;9(3212).

355. Gawryszewska I, Zabicka D, Hryniewicz W, Sadowy E. Linezolid-resistant enterococci in Polish hospitals: species, clonality and determinants of linezolid resistance. European journal of clinical microbiology & infectious diseases : official publication of the European Society of Clinical Microbiology. 2017;36(7):1279-86.

356. Di Pilato V, Pollini S, Rossolini GM. Tn*6249*, a new Tn*6162* transposon derivative carrying a double-integron platform and involved with acquisition of the *bla*_VIM-1_ metallo-β-lactamase gene in *Pseudomonas aeruginosa*. Antimicrobial agents and chemotherapy. 2015;59(3):1583-7.

357. Ou H-Y, Kuang SN, He X, Molgora BM, Ewing PJ, Deng Z, et al. Complete genome sequence of hypervirulent and outbreak-associated *Acinetobacter baumannii* strain LAC-4: epidemiology, resistance genetic determinants and potential virulence factors. Scientific Reports. 2015;5:8643.

358. Soriano N, Vincent P, Piau C, Moullec S, Gautier P, Lagente V, et al. Complete genome sequence of *Streptococcus pyogenes* M/emm44 strain STAB901, isolated in a clonal outbreak in French Brittany. Genome announcements. 2014;2(6).

359. Tanizawa Y, Tohno M, Kaminuma E, Nakamura Y, Arita M. Complete genome sequence and analysis of *Lactobacillus hokkaidonensis* LOOC260T, a psychrotrophic lactic acid bacterium isolated from silage. BMC Genomics. 2015;16(1):240.

360. Antonelli A, D'Andrea MM, Vaggelli G, Docquier J-D, Rossolini GM. OXA-372, a novel carbapenem-hydrolysing class D β-lactamase from a *Citrobacter freundii* isolated from a hospital wastewater plant. Journal of Antimicrobial Chemotherapy. 2015;70(10):2749-56.

361. Zhu X-Q, Wang X-M, Li H, Shang Y-H, Pan Y-S, Wu C-M, et al. Novel *lnu*(G) gene conferring resistance to lincomycin by nucleotidylation, located on Tn*6260* from *Enterococcus faecalis* E531. Journal of Antimicrobial Chemotherapy. 2016;72(4):993-7.

362. Sun C, Zhang P, Ji X, Fan R, Chen B, Wang Y, et al. Presence and molecular characteristics of oxazolidinone resistance in staphylococci from household animals in rural China. Journal of Antimicrobial Chemotherapy. 2018;73(5):1194-200.

363. Kambarev S, Cate C, Corvec S, Pecorari F. Draft Genome Sequence of Erythromycin-Resistant *Streptococcus gallolyticus* subsp. *gallolyticus* NTS 31106099 Isolated from a Patient with Infective Endocarditis and Colorectal Cancer. Genome announcements. 2015;3(2).

364. Phiwsaiya K, Charoensapsri W, Taengphu S, Dong HT, Sangsuriya P, Nguyen GTT, et al. A natural *Vibrio parahaemolyticus* Δ*pirA*^Vp^ *pirB*^Vp+^ mutant kills shrimp but produces neither Pir^Vp^ toxins nor acute hepatopancreatic necrosis disease lesions. Applied and environmental microbiology. 2017;83(16).

365. Tabata M, Ohhata S, Nikawadori Y, Kishida K, Sato T, Kawasumi T, et al. Comparison of the complete genome sequences of four γ-hexachlorocyclohexane-degrading bacterial strains: insights into the evolution of bacteria able to degrade a recalcitrant man-made pesticide. DNA Research. 2016;23(6):581-99.

366. Karah N, Dwibedi CK, Sjostrom K, Edquist P, Johansson A, Wai SN, et al. Novel aminoglycoside resistance transposons and transposon-derived circular forms detected in carbapenem-resistant *Acinetobacter baumannii* clinical isolates. Antimicrobial agents and chemotherapy. 2016;60(3):1801-18.

367. Nonaka L, Yamamoto T, Maruyama F, Hirose Y, Onishi Y, Kobayashi T, et al. Interplay of a non-conjugative integrative element and a conjugative plasmid in the spread of antibiotic resistance via suicidal plasmid transfer from an aquaculture *Vibrio* isolate. PloS one. 2018;13(6):e0198613.

368. Sun F, Zhou D, Wang Q, Feng J, Feng W, Luo W, et al. The first report of detecting the *bla*_SIM-2_ gene and determining the complete sequence of the SIM-encoding plasmid. Clinical Microbiology and Infection. 2016;22(4):347-51.

369. Sun F, Zhou D, Wang Q, Feng J, Feng W, Luo W, et al. Genetic characterization of a novel *bla*_DIM-2_-carrying megaplasmid p12969-DIM from clinical *Pseudomonas putida*. Journal of Antimicrobial Chemotherapy. 2015;71(4):909-12.

370. Hall JPJ, Williams D, Paterson S, Harrison E, Brockhurst MA. Positive selection inhibits gene mobilization and transfer in soil bacterial communities. Nature Ecology & Evolution. 2017;1(9):1348-53.

371. Feng W, Zhou D, Wang Q, Luo W, Zhang D, Sun Q, et al. Dissemination of IMP-4-encoding pIMP-HZ1-related plasmids among *Klebsiella pneumoniae* and *Pseudomonas aeruginosa* in a Chinese teaching hospital. Scientific Reports. 2016;6:33419.

372. Hargreaves KR, Thanki AM, Jose BR, Oggioni MR, Clokie MRJ. Use of single molecule sequencing for comparative genomics of an environmental and a clinical isolate of *Clostridium difficile* ribotype 078. BMC Genomics. 2016;17(1):1020.

373. Matsui K, Yoshinami S, Narita M, Chien M-F, Phung LT, Silver S, et al. Mercury resistance transposons in *Bacilli* strains from different geographical regions. FEMS Microbiology Letters. 2016;363(5).

374. Wang L, Liu L, Liu D, Yin Z, Feng J, Zhang D, et al. The first report of a fully sequenced resistance plasmid from *Shigella boydii*. Frontiers in Microbiology. 2016;7(1579).

375. Wang D, Zhu J, Zhou K, Chen J, Yin Z, Feng J, et al. Genetic characterization of novel class 1 Integrons In0, In1069 and In1287 to In1290, and the inference of In1069-associated integron evolution in *Enterobacteriaceae*. Antimicrobial Resistance & Infection Control. 2017;6(1):84.

376. Khadidja Y, Abdelaziz T, Brigitte L, Éric F, Jean-Charles C, Hafid S, et al. A novel plasmid, pSx1, harboring a new Tn*1696* derivative from extensively drug-resistant *Shewanella xiamenensis* encoding OXA-416. Microbial Drug Resistance. 2017;23(4):429-36.

377. Leclercq SO, Wang C, Zhu Y, Wu H, Du X, Liu Z, et al. Diversity of the tetracycline mobilome within a Chinese pig manure sample. Applied and environmental microbiology. 2016;82(21):6454-62.

378. Zhang F, Wang X, Xie L, Zheng Q, Guo X, Han L, et al. A novel transposon, Tn*6306*, mediates the spread of bla_IMI_ in Enterobacteriaceae in hospitals. International journal of infectious diseases : IJID : official publication of the International Society for Infectious Diseases. 2017;65:22-6.

379. Sun F, Zhou D, Sun Q, Luo W, Tong Y, Zhang D, et al. Genetic characterization of two fully sequenced multi-drug resistant plasmids pP10164-2 and pP10164-3 from *Leclercia adecarboxylata*. Scientific Reports. 2016;6:33982.

380. Carraro N, Rivard N, Ceccarelli D, Colwell RR, Burrus V. IncA/C conjugative plasmids mobilize a new family of multidrug resistance islands in clinical *Vibrio cholerae* non-O1/non-O139 isolates from Haiti. mBio. 2016;7(4).

381. Jiang X, Yin Z, Yin X, Fang H, Sun Q, Tong Y, et al. Sequencing of *bla*_IMP_-carrying IncN2 plasmids, and comparative genomics of IncN2 plasmids harboring class 1 integrons. Frontiers in Cellular and Infection Microbiology. 2017;7(102).

382. Barraud O, Isnard C, Lienhard R, Guérin F, Couvé-Deacon E, Martin C, et al. Sulphonamide resistance associated with integron derivative Tn*6326* in *Actinotignum schaalii*. Journal of Antimicrobial Chemotherapy. 2016;71(9):2670-1.

383. Beyrouthy R, Robin F, Hamze M, Bonnet R. IncFII_k_ plasmid harbouring an amplification of 16S rRNA methyltransferase-encoding gene *rmtH* associated with mobile element IS*CR2*. Journal of Antimicrobial Chemotherapy. 2016;72(2):402-6.

384. Snesrud E, McGann P, Chandler M. The birth and demise of the IS*Apl1*-*mcr-1*-IS*Apl1* composite transposon: the vehicle for transferable colistin resistance. mBio. 2018;9(1).

385. Kambarev S, Pecorari F, Corvec S. Draft genome sequences of two highly erythromycin-resistant *Streptococcus gallolyticus* subsp. *gallolyticus* isolates containing a novel Tn*916*-like element, Tn*6331*. Genome announcements. 2017;5(16).

386. Szabo M, Nagy T, Wilk T, Farkas T, Hegyi A, Olasz F, et al. Characterization of two multidrug-resistant IncA/C plasmids from the 1960s by using the MinION sequencer device. Antimicrobial agents and chemotherapy. 2016;60(11):6780-6.

387. Liang Q, Jiang X, Hu L, Yin Z, Gao B, Zhao Y, et al. Sequencing and genomic diversity analysis of IncHI5 plasmids. Frontiers in Microbiology. 2019;9(3318).

388. Ng SP, Davis B, Palombo EA, Bhave M. A Tn*5051*-like mer-containing transposon identified in a heavy metal tolerant strain *Achromobacter sp.* AO22. BMC Research Notes. 2009;2(1):38.

389. Turano H, Gomes F, Barros-Carvalho GA, Lopes R, Cerdeira L, Netto LES, et al. Tn*6350*, a novel transposon carrying pyocin S8 genes encoding a bacteriocin with activity against carbapenemase-producing *Pseudomonas aeruginosa*. Antimicrobial agents and chemotherapy. 2017;61(5).

390. Botelho J, Grosso F, Peixe L. Characterization of the pJB12 plasmid from *Pseudomonas aeruginosa* reveals Tn*6352*, a novel putative transposon associated with mobilization of the *bla*_VIM-2_-Harboring In58 Integron. Antimicrobial agents and chemotherapy. 2017;61(5).

391. Botelho J, Grosso F, Quinteira S, Mabrouk A, Peixe L. The complete nucleotide sequence of an IncP-2 megaplasmid unveils a mosaic architecture comprising a putative novel *bla*_VIM-2_-harbouring transposon in *Pseudomonas aeruginosa*. Journal of Antimicrobial Chemotherapy. 2017;72(8):2225-9.

392. Zhao Y, Wang L, Zhang Z, Feng J, Kang H, Fang L, et al. Structural genomics of pNDM-BTR harboring In191 and Tn*6360*, and other *bla*_NDM_-carrying IncN1 plasmids. Future Microbiology. 2017;12(14):1271-81.

393. Shi L, Liang Q, Zhan Z, Feng J, Zhao Y, Chen Y, et al. Co-occurrence of 3 different resistance plasmids in a multi-drug resistant *Cronobacter sakazakii* isolate causing neonatal infections. Virulence. 2018;9(1):110-20.

394. Shi L, Feng J, Zhan Z, Zhao Y, Zhou H, Mao H, et al. Comparative analysis of *bla*_KPC-2_- and *rmtB*-carrying IncFII-family pKPC-LK30/pHN7A8 hybrid plasmids from *Klebsiella pneumoniae* CG258 strains disseminated among multiple Chinese hospitals. Infection and drug resistance. 2018;11:1783-93.

395. Marquez-Ortiz RA, Haggerty L, Olarte N, Duarte C, Garza-Ramos U, Silva-Sanchez J, et al. Genomic epidemiology of NDM-1-encoding plasmids in Latin American clinical isolates reveals insights into the evolution of multidrug resistance. Genome Biology and Evolution. 2017;9(6):1725-41.

396. Stalder T, Rogers LM, Renfrow C, Yano H, Smith Z, Top EM. Emerging patterns of plasmid-host coevolution that stabilize antibiotic resistance. Scientific Reports. 2017;7(1):4853.

397. Zhan Z, Hu L, Jiang X, Zeng L, Feng J, Wu W, et al. Plasmid and chromosomal integration of four novel *bla*_IMP_-carrying transposons from *Pseudomonas aeruginosa*, *Klebsiella pneumoniae* and an *Enterobacter* sp. Journal of Antimicrobial Chemotherapy. 2018;73(11):3005-15.

398. Li B, Feng J, Zhan Z, Yin Z, Jiang Q, Wei P, et al. Dissemination of KPC-2-encoding IncX6 plasmids among multiple *Enterobacteriaceae* species in a single Chinese hospital. Frontiers in Microbiology. 2018;9(478).

399. Liang B, Roberts AP, Xu X, Yang C, Yang X, Wang J, et al. Transferable plasmid-borne *mcr-1* in a colistin-resistant *Shigella flexneri* isolate. Applied and environmental microbiology. 2018;84(8).

400. Zeng L, Zhan Z, Hu L, Jiang X, Zhang Y, Feng J, et al. Genetic characterization of a bla_VIM–24_-carrying IncP-7β plasmid p1160-VIM and a *bla*_VIM–4_-Harboring Integrative and Conjugative Element Tn*6413* From Clinical *Pseudomonas aeruginosa*. Frontiers in Microbiology. 2019;10(213).

401. Zhou K, Yu W, Shen P, Lu H, Wang B, Rossen JWA, et al. A novel Tn*1696*-like composite transposon (Tn*6404*) harboring *bla_I_*_MP-4_ in a *Klebsiella pneumoniae* isolate carrying a rare ESBL gene *bla*_SFO-1_. Scientific Reports. 2017;7(1):17321.

402. Zhang D, Zhao Y, Feng J, Hu L, Jiang X, Zhan Z, et al. Replicon-based typing of IncI-complex plasmids, and comparative genomics analysis of IncIγ/K1 plasmids. Frontiers in Microbiology. 2019;10(48).

403. Shi Y, Tian Z, Leclercq SO, Zhang H, Yang M, Zhang Y. Genetic characterization and potential molecular dissemination mechanism of *tet*(31) gene in *Aeromonas caviae* from an oxytetracycline wastewater treatment system. Journal of environmental sciences (China). 2019;76:259-66.

404. Magagnin CM, Campos JC, da Rocha DA, Sampaio SCF, Rozales FP, Barth AL, et al. Dissemination of *bla*_OXA-370_ is mediated by IncX plasmids and the Tn*6435* transposon. European journal of clinical microbiology & infectious diseases : official publication of the European Society of Clinical Microbiology. 2018;37(11):2165-9.

405. Chen YP, Lei CW, Kong LH, Zeng JX, Zhang XZ, Liu BH, et al. Tn*6450*, a novel multidrug resistance transposon characterized in a *Proteus mirabilis* isolate from chicken in China. Antimicrobial agents and chemotherapy. 2018;62(4).

406. Chen Y, Lei C, Zuo L, Kong L, Kang Z, Zeng J, et al. A novel *cfr*-carrying Tn*7* transposon derivative characterized in *Morganella morganii* of swine origin in China. Journal of Antimicrobial Chemotherapy. 2018;74(3):603-6.

407. Borowiak M, Hammerl JA, Deneke C, Fischer J, Szabo I, Malorny B. Characterization of *mcr-5*-Harboring *Salmonella enterica* subsp. *enterica* Serovar Typhimurium Isolates from Animal and Food Origin in Germany. Antimicrobial agents and chemotherapy. 2019;63(6).

408. Veloo ACM, Chlebowicz M, Winter HLJ, Bathoorn D, Rossen JWA. Three metronidazole-resistant *Prevotella bivia* strains harbour a mobile element, encoding a novel *nim* gene, *nimK*, and an efflux small MDR transporter. Journal of Antimicrobial Chemotherapy. 2018;73(10):2687-90.

409. Shen D, Ma G, Li C, Jia X, Qin C, Yang T, et al. Emergence of a multidrug-resistant hypervirulent *Klebsiella pneumoniae* sequence type 23 strain with a rare *bla*_CTX-M-24_-harboring virulence plasmid. Antimicrobial agents and chemotherapy. 2019;63(3).

410. Romaniuk K, Golec P, Dziewit L. Insight into the diversity and possible role of plasmids in the adaptation of psychrotolerant and metalotolerant *Arthrobacter* spp. to extreme antarctic environments. Frontiers in Microbiology. 2018;9(3144).

411. Sun Y-w, Liu Y-y, Wu H, Wang L-f, Liu J-h, Yuan L, et al. IS*26*-flanked composite transposon Tn*6539* carrying the *tet*(M) gene in IncHI2-type conjugative plasmids from *Escherichia coli* isolated from ducks in China. Frontiers in Microbiology. 2019;9(3168).

412. Beyrouthy R, Barets M, Marion E, Dananché C, Dauwalder O, Robin F, et al. Novel *Enterobacter* lineage as leading cause of nosocomial outbreak involving carbapenemase-producing strains. Emerging infectious diseases. 2018;24(8):1505-15.

413. Morroni G, Brenciani A, Antonelli A, Maria D’Andrea M, Di Pilato V, Fioriti S, et al. Characterization of a multiresistance plasmid carrying the *optrA* and *cfr* resistance genes from an *Enterococcus faecium* clinical isolate. Frontiers in Microbiology. 2018;9(2189).

414. Wang Y, Li D, Song L, Liu Y, He T, Liu H, et al. First report of the multiresistance gene *cfr* in *Streptococcus suis*. Antimicrobial agents and chemotherapy. 2013;57(8):4061-3.

415. Adams FG, Brown MH. MITE_Aba12_ , a novel mobile miniature inverted-repeat transposable element identified in *Acinetobacter baumannii* ATCC 17978 and its prevalence across the *Moraxellaceae* Family. mSphere. 2019;4(1).

416. Leon-Sampedro R, Fernandez-de-Bobadilla MD, San Millan A, Baquero F, Coque TM. Transfer dynamics of Tn*6648*, a composite integrative conjugative element generated by tandem accretion of Tn*5801* and Tn*6647* in *Enterococcus faecalis*. The Journal of antimicrobial chemotherapy. 2019.

417. Di Pilato V, Papa-Ezdra R, Chiarelli A, García- Fulgueiras V, Pallecchi L, Vignoli R. Characterization of the first *bla*_CTX-M-14_/*ermB*-carrying IncI1 plasmid from Latin America. Plasmid. 2019;102:1-5.

418. Boyd DA, Mataseje LF, Pelude L, Mitchell R, Bryce E, Roscoe D, et al. Results from the Canadian Nosocomial Infection Surveillance Program for detection of carbapenemase-producing *Acinetobacter* spp. in Canadian hospitals, 2010–16. Journal of Antimicrobial Chemotherapy. 2018;74(2):315-20.

419. Li D, Li XY, Schwarz S, Yang M, Zhang SM, Hao W, et al. Tn*6674*, a novel enterococcal *optrA*-carrying multiresistance transposon of the Tn*554* family. Antimicrobial agents and chemotherapy. 2019.

420. Klompe SE, Vo PLH, Halpin-Healy TS, Sternberg SH. Transposon-encoded CRISPR–Cas systems direct RNA-guided DNA integration. Nature. 2019.
