# Supplementary material for: The Transposon Registry
Source: Mob DNA. 2019 Oct 9;10:40. doi: 10.1186/s13100-019-0182-3 (PMC6785933; doi:10.1186/s13100-019-0182-3)
Supplement: Supplementary file 2 — Additional file 2: Table S2. List of transposons associated with antibiotic resistance genes (DOCX 24 kb) [file 13100_2019_182_MOESM2_ESM.docx]

**Additional file 2: Table S2 List of transposons associated with antibiotic resistance genes**

| Antibiotic classes | Tn records |
| --- | --- |
| Aminoglycoside | Tn*4*, Tn*5*, Tn*6*, Tn*7*, Tn*21*, Tn*55*, Tn*71*, Tn*72*, Tn*76*, Tn*510*, Tn*511*, Tn*536*, Tn*554*, Tn*601*, Tn*602*, Tn*611*, Tn*654*, Tn*679*, Tn*732*, Tn*733*, Tn*734*, Tn*800*, Tn*813*, Tn*903*, Tn*904*, Tn*924*, Tn*1331*, Tn*1332*, Tn*1401*, Tn*1402*, Tn*1403*, Tn*1404*, Tn*1405*, Tn*1525*, Tn*1527*, Tn*1528*, Tn*1545*, Tn*1548*, Tn*1696*, Tn*1699*, Tn*1700*, Tn*1723*, Tn*1732*, Tn*1755*, Tn*1792*, Tn*1824*, Tn*1825*, Tn*1831*, Tn*1935*, Tn*2000*, Tn*2011*, Tn*2101*, Tn*2350*, Tn*2353*, Tn*2401*, Tn*2411*, Tn*2424*, Tn*2425*, Tn*2426*, Tn*2507*, Tn*2515*, Tn*2521*, Tn*2523*, Tn*2603*, Tn*2607*, Tn*2608*, Tn*2610*, Tn*2670*, Tn*2671*, Tn*2672*, Tn*2680*, Tn*2681*, Tn*2922*, Tn*3613*, Tn*3706*, Tn*3851*, Tn*3853*, Tn*3854*, Tn*4000*, Tn*4001*, Tn*4031*, Tn*4352*, Tn*4411*, Tn*4527*, Tn*4551*, Tn*5045*, Tn*5062*, Tn*5096*, Tn*5097*, Tn*5098*, Tn*5099*, Tn*5384*, Tn*5385*, Tn*5393*, Tn*5404*, Tn*5405*, Tn*5466*, Tn*5495*, Tn*5531*, Tn*6001*, Tn*6003*, Tn*6004*, Tn*6016*, Tn*6017*, Tn*6020*, Tn*6023*, Tn*6026*, Tn*6027*, Tn*6028*, Tn*6029*, Tn*6031*, Tn*6060*, Tn*6061*, Tn*6072*, Tn*6082*, Tn*6088*, Tn*6108*, Tn*6133*, Tn*6162*, Tn*6163*, Tn*6164*, Tn*6166*, Tn*6167*, Tn*6172*, Tn*6178*, Tn*6179*, Tn*6180*, Tn*6183*, Tn*6187*, Tn*6203*, Tn*6204*, Tn*6205*, Tn*6207*, Tn*6210*, Tn*6214*, Tn*6216*, Tn*6217*, Tn*6218*, Tn*6229*, Tn*6234*, Tn*6235*, Tn*6238*, Tn*6242*, Tn*6249*, Tn*6250*, Tn*6263*, Tn*6279*, Tn*6284*, Tn*6285*, Tn*6286*, Tn*6293*, Tn*6297*, Tn*6299*, Tn*6301*, Tn*6308*, Tn*6321*, Tn*6331*, Tn*6338*, Tn*6340*, Tn*6345*, Tn*6352*, Tn*6356*, Tn*6363*, Tn*6375*, Tn*6378*, Tn*6382*, Tn*6383*, Tn*6384*, Tn*6392*, Tn*6393*, Tn*6394*, Tn*6397*, Tn*6400*, Tn*6401*, Tn*6402*, Tn*6403*, Tn*6404*, Tn*6411*, Tn*6413*, Tn*6414*, Tn*6417*, Tn*6431*, Tn*6450*, Tn*6451*, Tn*6505*, Tn*6530*, Tn*6531*, Tn*6532*, Tn*6533*, Tn*6534*, Tn*6540*, Tn*6649*, Tn*6674* |
| Amphenicol | Tn*45*, Tn*9*, Tn*558*, Tn*981*, Tn*1403*, Tn*1696*, Tn*1725*, Tn*2000*, Tn*2001*, Tn*2424*, Tn*2426*, Tn*2506*, Tn*2507*, Tn*2516*, Tn*2653*, Tn*2670*, Tn*2671*, Tn*2672*, Tn*2700*, Tn*3351*, Tn*3352*, Tn*3951*, Tn*4451*, Tn*4452*, Tn*5252*, Tn*5253*, Tn*5564*, Tn*6027*, Tn*6058*, Tn*6060*, Tn*6061*, Tn*6088*, Tn*6089*, Tn*6180*, Tn*6187*, Tn*6214*, Tn*6217*, Tn*6218*, Tn*6229*, Tn*6246*, Tn*6248*, Tn*6249*, Tn*6261*, Tn*6279*, Tn*6284*, Tn*6285*, Tn*6286*, Tn*6297*, Tn*6299*, Tn*6301*, Tn*6308*, Tn*6383*, Tn*6384*, Tn*6393*, Tn*6400*, Tn*6401*, Tn*6410*, Tn*6431*, Tn*6450*, Tn*6451*, Tn*6530*, Tn*6531*, Tn*6533*, Tn*6534*, Tn*6644*, Tn*6674* |
| Anthracycline | Tn*6097* |
| β-lactam | Tn*1*, Tn*2*, Tn*3*, Tn*125*, Tn*552*, Tn*654*, Tn*801*, Tn*802*, Tn*841*, Tn*901*, Tn*902*, Tn*1213,* Tn*1331*, Tn*1332*, Tn*1401*, Tn*1402*, Tn*1405*, Tn*1699*, Tn*1700*, Tn*1701*, Tn*1755*, Tn*1756*, Tn*1935*, Tn*1999*, Tn*2000*, Tn*2003*, Tn*2006*, Tn*2007*, Tn*2008*, Tn*2011*, Tn*2016*, Tn*2101*, Tn*2301*, Tn*2410*, Tn*2521*, Tn*2601*, Tn*2602*, Tn*2603*, Tn*2607*, Tn*2610*, Tn*2660*, Tn*2922*, Tn*3000*, Tn*3651*, Tn*4002*, Tn*4176*, Tn*4201*, Tn*4291*, Tn*4401*, Tn*4555*, Tn*5385*, Tn*5386*, Tn*5431*, Tn*6001*, Tn*6004*, Tn*6013*, Tn*6016*, Tn*6017*, Tn*6026*, Tn*6027*, Tn*6029*, Tn*6045*, Tn*6060*, Tn*6061*, Tn*6080*, Tn*6088*, Tn*6092*, Tn*6093*, Tn*6108*, Tn*6113*, Tn*6114*, Tn*6163*, Tn*6167*, Tn*6168*, Tn*6181*, Tn*6186*, Tn*6187*, Tn*6191*, Tn*6203*, Tn*6206*, Tn*6214*, Tn*6216*, Tn*6217*, Tn*6222*, Tn*6234*, Tn*6237*, Tn*6238*, Tn*6240*, Tn*6242*, Tn*6249*, Tn*6252*, Tn*6255*, Tn*6256*, Tn*6284*, Tn*6296*, Tn*6297*, Tn*6306*, Tn*6308*, Tn*6320*, Tn*6329*, Tn*6338*, Tn*6339*, Tn*6345*, Tn*6352*, Tn*6356*, Tn*6360*, Tn*6361*, Tn*6367*, Tn*6375*, Tn*6377*, Tn*6378*, Tn*6382*, Tn*6383*, Tn*6384*, Tn*6391*, Tn*6392*, Tn*6394*, Tn*6397*, Tn*6400*, Tn*6401*, Tn*6403*, Tn*6404*, Tn*6411*, Tn*6413*, Tn*6414*, Tn*6435*, Tn*6450*, Tn*6451*, Tn*6501*, Tn*6505*, Tn*6530*, Tn*6531*, Tn*6533*, Tn*6534*, Tn*6540*, Tn*6649*, Tn*6652*, Tn*6655*, Tn*6656* |
| Colistin | Tn*6330*, Tn*6390*, Tn*6452*, Tn*6518* |
| Fosfomycin | Tn*2921*, Tn*6001* |
| Glycopeptide | Tn*5*, Tn*1546*, Tn*1547*, Tn*1549*, Tn*5382*, Tn*6074*, Tn*6083*, Tn*6202* |
| Lincomycin | Tn*917*, Tn*1545*, Tn*3853*, Tn*4551*, Tn*5030*, Tn*5432*, Tn*6133*, Tn*6260*, Tn*6450*, Tn*6451*, Tn*6644* |
| Macrolide | Tn*551*, Tn*554*, Tn*917*, Tn*950*, Tn*1116*, Tn*1207.1*, Tn*1545*, Tn*1806*, Tn*2009*, Tn*2010*, Tn*2017*, Tn*3701*, Tn*3703*, Tn*3704*, Tn*3705*, Tn*3853*, Tn*3871*, Tn*3872*, Tn*3951*, Tn*4351*, Tn*4400*, Tn*4551*, Tn*5384*, Tn*5385*, Tn*5432*, Tn*5466*, Tn*5506*, Tn*6002*, Tn*6003*, Tn*6058*, Tn*6079*, Tn*6164*, Tn*6183*, Tn*6194*, Tn*6215*, Tn*6218*, Tn*6242*, Tn*6261*, Tn*6263*, Tn*6279*, Tn*6284*, Tn*6285*, Tn*6295*, Tn*6297*, Tn*6308*, Tn*6331*, Tn*6378*, Tn*6383*, Tn*6393*, Tn*6397*, Tn*6403*, Tn*6413*, Tn*6450*, Tn*6644*, Tn*6651* |
| Nitroimidazole | Tn*6456* |
| Nucleoside antibiotic | Tn*6383*, Tn*6411* |
| Oxazolidinone | Tn*6261*, Tn*6628*, Tn*6644*, Tn*6674* |
| Pleuromutilin | Tn*6133*, Tn*6644* |
| Quinolone | Tn*2012*, Tn*6010*, Tn*6074*, Tn*6083*, Tn*6218*, Tn*6238*, Tn*6286*, Tn*6292*, Tn*6297*, Tn*6308*, Tn*6325*, Tn*6338*, Tn*6340*, Tn*6360*, Tn*6361*, Tn*6384*, Tn*6393*, Tn*6400*, Tn*6450* |
| Rifamycins | Tn*6061*, Tn*6229*, Tn*6284*, Tn*6308*, Tn*6338*, Tn*6340*, Tn*6384*, Tn*6400*, Tn*6401*, Tn*6450*, Tn*6451* |
| Streptogramin | Tn*1545*, Tn*5406*, Tn*5432*, Tn*6133*, Tn*6218*, Tn*6644* |
| Streptothricin | Tn*1825*, Tn*1826*, Tn*2425*, Tn*6003*, Tn*6031*, Tn*6181*, Tn*6451* |
| Sulphonamide | Tn*4*, Tn*21*, Tn*536*, Tn*610*, Tn*800*, Tn*813*, Tn*1401*, Tn*1402*, Tn*1404*, Tn*1405*, Tn*1548*, Tn*1696*, Tn*1935*, Tn*2000*, Tn*2011*, Tn*2101*, Tn*2353*, Tn*2410*, Tn*2411*, Tn*2425*, Tn*2426*, Tn*2521*, Tn*2603*, Tn*2607*, Tn*2608*, Tn*2610*, Tn*2670*, Tn*2671*, Tn*2672*, Tn*3613*, Tn*4000*, Tn*5045*, Tn*5086*, Tn*6001*, Tn*6025*, Tn*6026*, Tn*6027*, Tn*6029*, Tn*6060*, Tn*6061*, Tn*6088*, Tn*6108*, Tn*6167*, Tn*6172*, Tn*6180*, Tn*6187*, Tn*6203*, Tn*6208*, Tn*6209*, Tn*6214*, Tn*6216*, Tn*6217*, Tn*6234*, Tn*6242*, Tn*6249*, Tn*6250*, Tn*6279*, Tn*6284*, Tn*6286*, Tn*6297*, Tn*6299*, Tn*6300*, Tn*6301*, Tn*6302*, Tn*6321*, Tn*6325*, Tn*6326*, Tn*6338*, Tn*6383*, Tn*6384*, Tn*6391*, Tn*6393*, Tn*6400*, Tn*6401*, Tn*6403*, Tn*6413*, Tn*6414*, Tn*6417*, Tn*6431*, Tn*6432*, Tn*6450*, Tn*6530*, Tn*6531*, Tn*6532*, Tn*6533*, Tn*6534*, Tn*6649* |
| Tetracycline | Tn*10*, Tn*701*, Tn*804*, Tn*916*, Tn*918*, Tn*919*, Tn*925*, Tn*1021*, Tn*1022*, Tn*1404*, Tn*1523*, Tn*1545*, Tn*1720*, Tn*1721*, Tn*1756*, Tn*1771*, Tn*1822*, Tn*2009*, Tn*2010*, Tn*2017*, Tn*2440*, Tn*2515*, Tn*2516*, Tn*2653*, Tn*2657*, Tn*3701*, Tn*3702*, Tn*3703*, Tn*3704*, Tn*3705*, Tn*3707*, Tn*3872*, Tn*3951*, Tn*4351*, Tn*4400*, Tn*4431*, Tn*4731*, Tn*5030*, Tn*5031*, Tn*5032*, Tn*5033*, Tn*5251*, Tn*5253*, Tn*5381*, Tn*5383*, Tn*5385*, Tn*5397*, Tn*5431*, Tn*5491*, Tn*5706*, Tn*5801*, Tn*6000*, Tn*6002*, Tn*6003*, Tn*6009*, Tn*6014*, Tn*6031*, Tn*6058*, Tn*6061*, Tn*6079*, Tn*6084*, Tn*6085*, Tn*6086*, Tn*6087*, Tn*6088*, Tn*6099*, Tn*6100*, Tn*6108*, Tn*6164*, Tn*6166*, Tn*6167*, Tn*6183*, Tn*6190*, Tn*6194*, Tn*6198*, Tn*6207*, Tn*6224*, Tn*6227*, Tn*6247*, Tn*6248*, Tn*6253*, Tn*6297*, Tn*6298*, Tn*6299*, Tn*6300*, Tn*6301*, Tn*6302*, Tn*6303*, Tn*6308*, Tn*6309*, Tn*6432*, Tn*6530*, Tn*6531*, Tn*6533*, Tn*6534*, Tn*6539*, Tn*6648*, Tn*6649* |
| Thiopeptide | Tn*5492*, Tn*5493* |
| Trimethoprim | Tn*7*, Tn*71*, Tn*72*, Tn*735*, Tn*76*, Tn*813*, Tn*1527*, Tn*1548*, Tn*1824*, Tn*4003*, Tn*4132*, Tn*4527*, Tn*5086*, Tn*5090*, Tn*536*, Tn*559*, Tn*6025*, Tn*6026*, Tn*6027*, Tn*6028*, Tn*6088*, Tn*6181*, Tn*6198*, Tn*6222*, Tn*6242*, Tn*6286*, Tn*6297*, Tn*6325*, Tn*6338*, Tn*6402*, Tn*6414*, Tn*6450*, Tn*6451*, Tn*6540*, Tn*6649* |
| Tuberactinomycin | Tn*4560*, Tn*4563* |
